# Short-term temperature fluctuations increase disease in a *Daphnia*-parasite infectious disease system

**DOI:** 10.1371/journal.pbio.3002260

**Published:** 2023-09-08

**Authors:** Leila Krichel, Devin Kirk, Clara Pencer, Madison Hönig, Kiran Wadhawan, Martin Krkošek

**Affiliations:** 1 Department of Ecology and Evolutionary Biology, University of Toronto, Toronto, Canada; 2 Department of Biology, Stanford University, Stanford, California, United States of America; 3 Department of Anthropology, Washington State University, Pullman, Washington, United States of America; 4 School of Biological Sciences, University of Edinburgh, Edinburgh, United Kingdom; The Pennsylvania State University, UNITED STATES

## Abstract

Climate change has profound effects on infectious disease dynamics, yet the impacts of increased short-term temperature fluctuations on disease spread remain poorly understood. We empirically tested the theoretical prediction that short-term thermal fluctuations suppress endemic infection prevalence at the pathogen’s thermal optimum. This prediction follows from a mechanistic disease transmission model analyzed using stochastic simulations of the model parameterized with thermal performance curves (TPCs) from metabolic scaling theory and using nonlinear averaging, which predicts ecological outcomes consistent with Jensen’s inequality (i.e., reduced performance around concave-down portions of a thermal response curve). Experimental observations of replicated epidemics of the microparasite *Ordospora colligata* in *Daphnia magna* populations indicate that temperature variability had the opposite effect of our theoretical predictions and instead increase endemic infection prevalence. This positive effect of temperature variability is qualitatively consistent with a published hypothesis that parasites may acclimate more rapidly to fluctuating temperatures than their hosts; however, incorporating hypothetical effects of delayed host acclimation into the mechanistic transmission model did not fully account for the observed pattern. The experimental data indicate that shifts in the distribution of infection burden underlie the positive effect of temperature fluctuations on endemic prevalence. The increase in disease risk associated with climate fluctuations may therefore result from disease processes interacting across scales, particularly within-host dynamics, that are not captured by combining standard transmission models with metabolic scaling theory.

## Abstract 2

Experiments using a Daphnia-microparasite system reveal that disease transmission models that only model transmission dynamics at the among-host scale incorrectly predict the direction of effect of short-term thermal variability on endemic infection prevalence.

## Introduction

Climate change and infectious diseases threaten the integrity of many human and ecological systems, with profound effects on biodiversity [1–3], habitat ranges [4–6], human health [7,8], and food security [9]. Additionally, the link between climate change and accelerated rates of emerging infectious diseases has prompted research aimed at identifying which diseases will pose the greatest risk under future conditions [8,10]. Here, temperature shifts have received notable attention as this mediates the outcome of many host–parasite interactions [11–14]. However, while temperature shifts are characterized by changes to the mean and variance, the latter has often been overlooked. Plastic responses [2,3,11,15,16], energetic and metabolic demands [11,17], nonlinearities in thermal performance curves (TPCs) [14,18–21], and the patterns and timescales of variability [2,22–24] all mediate species responses’ to fluctuating temperatures in nuanced and sometimes unintuitive ways. This adds a source of complexity to ecological processes and challenges our ability to quantify the thermal conditions where infectious diseases are expected to be favored.

Different patterns of temperature change may confront ecological systems and present a challenge for ecologists in identifying the most relevant timescales on which they occur. Seasonal environmental changes occur on long timescales and can modify components of disease transmission, including the abundance of vectors or susceptible hosts, host behavior, host immunity, and parasite survivorship in the environment [24]. Less studied are the effects of short-term thermal fluctuations. Rohr and Raffel’s [3] temperature variability hypothesis posits that short-term thermal variability (e.g., daily, weekly) may elevate infection risk in hosts due to differences in host and parasite thermal acclimation rates. Here, parasites are hypothesized to acclimate more rapidly to short-term thermal fluctuations due to being smaller than their hosts, thereby increasing host susceptibility to infection [2,3,25,26]. Further, Raffel and colleagues [2] show that relative to predictable short-term temperature fluctuations (e.g., diurnal fluctuations), hosts in unpredictable thermal environments should be less resistant to infection due to the combination of being unable to anticipate novel temperatures and the parasite’s ability to more quickly acclimate to them. Short-term temperature fluctuations introduce complex and unique host–parasite responses that are dependent on the pattern of thermal variation being imposed on the system [23]. Understanding the nature of these responses is imperative in the face of climate models projecting increased environmental variability and extreme events [27,28].

**Fig 1:**
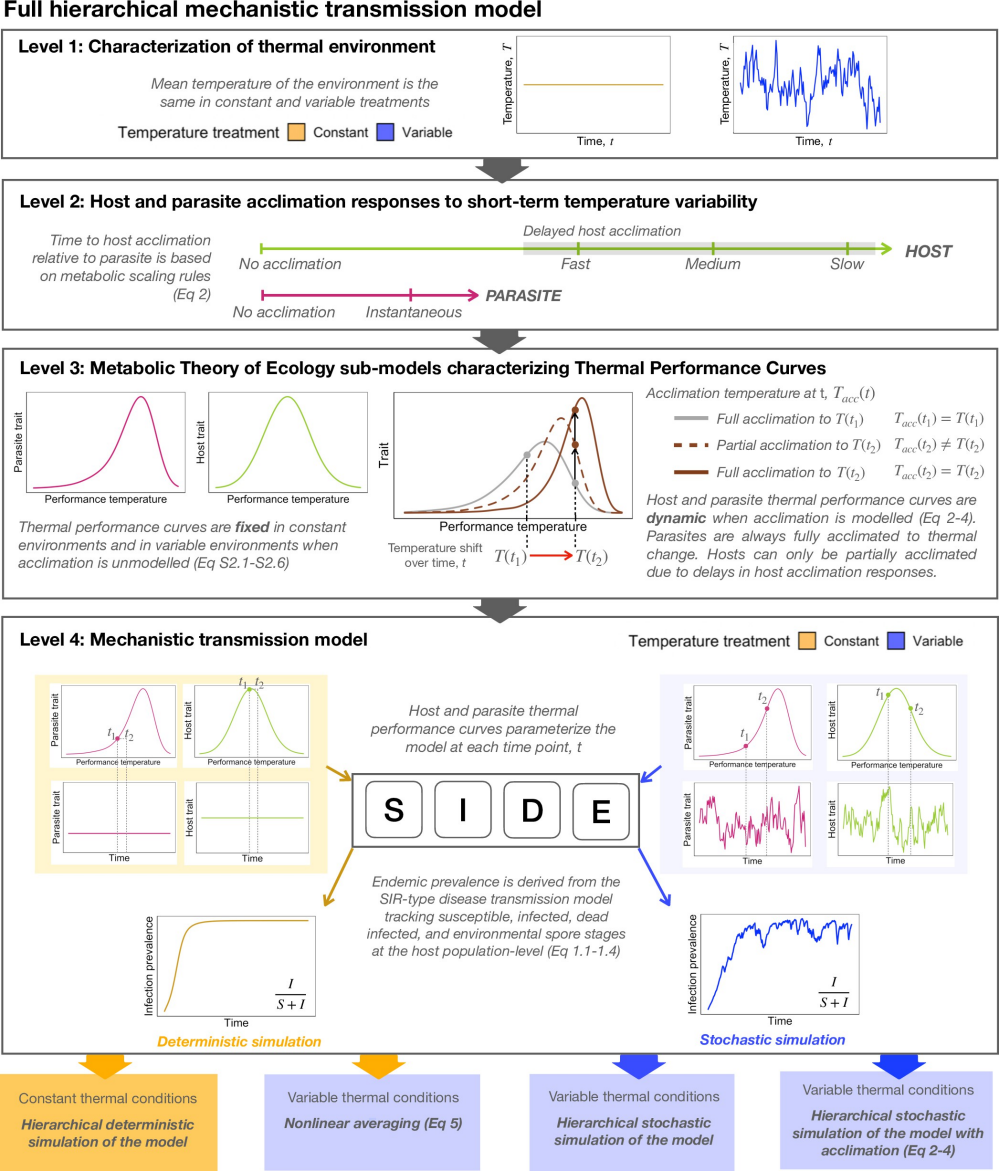
Flow diagram of full hierarchical mechanistic transmission model. The mechanistic transmission model is a hierarchical model that allows the effects of temperature to directly affect disease transmission dynamics and can be used to derive endemic infection prevalence. It is a model comprised of 4 distinct levels. Level 1 characterizes the temperature of the environment as thermally constant or thermally variable over time. Level 2 models host–parasite acclimation responses to temperature variability. No acclimation refers to the scenario where acclimation responses of the host and the parasite are not modeled. Instantaneous acclimation refers to the scenario where acclimation occurs immediately following a temperature shift. This scenario is reserved for parasites, such that the acclimation temperature of the parasite is always equal to the environmental temperature at time *t*. Delays in host acclimation were considered under fast, medium, and slow time to host acclimation scenarios (Eq 2-4). Since the acclimation temperature of the host is always lagging behind the environmental temperature at each time point, hosts may only be partially acclimated to a given temperature. Level 3 characterizes TPCs for host and parasite traits using MTE sub-models (S2.1–S2.6). When acclimation is not modeled, the shape of host and parasite TPCs is fixed over time. When acclimation is modeled, the shape of host and parasite TPCs is dynamic (note that in this figure, the depicted change in the TPC in response to a temperature shift is only meant to be illustrative). Since hosts exhibit delayed acclimation responses, they will always be partially acclimated to a temperature shift. Level 4 uses TPCs to parameterize the mechanistic transmission model at each time step, thus enabling a comparison of transmission dynamics in constant and variable environments. We used this model to obtain predictions of endemic prevalence in 4 different ways. First, hierarchical deterministic simulations of the model were used to numerically derive endemic prevalence in constant thermal environments as the proportion of infected hosts in the total population under equilibrium conditions. Second, nonlinear averaging was applied to the deterministic prediction of endemic prevalence across mean temperatures to obtain endemic prevalence in variable environments. Third, hierarchical stochastic simulations of the model were used to numerically derive endemic prevalence in variable environments—this model does not account for acclimation effects so that TPCs are fixed over time. Finally, hierarchical stochastic simulations of the model accounting for acclimation were used to numerically derive endemic prevalence in variable environments. MTE, metabolic theory of ecology; TPC, thermal performance curve.

Mechanistic disease transmission models are a powerful tool for clarifying how temperature influences disease transmission dynamics among hosts as they provide a mechanistic link between climate change effects and disease [13,29]. Such models may be described as hierarchical models as they let population-level transmission dynamics depend on the nonlinear thermal dependence of host and parasite traits (Fig 1). These models can be used to generate predictions for net disease outcomes (e.g., R0 or endemic prevalence) at different constant temperatures [6,30–33]. They can also be used to simulate net disease outcomes in thermally fluctuating environments by allowing the expression of trait TPCs parameterizing the model to vary with time. However, this form of parameterization can be arduous, time-consuming, and intractable, as it requires empirically quantifying host and parasite TPCs for multiple parameters in a mechanistic transmission model. This can be especially problematic for data-poor systems, thus limiting the utility of such models to well-studied diseases [34].

However, the metabolic theory of ecology (MTE) provides a possible solution to these data limitations [34,35]. MTE posits that metabolism predictably scales with body size and temperature and that these relationships are relatively conserved across taxa and levels of ecological organization [35]. Through metabolic scaling rules, this partially bypasses the need to empirically parameterize TPCs separately for each system, while offering testable approximations of disease net dynamics under different thermal conditions [30,33]. TPCs derived from metabolic scaling theory have been successful in quantifying individual-level host and parasite traits [36,37] in constant thermal environments, as well as population-level disease responses in slow warming environments [33], but have yet to be tested in response to short-term temperature fluctuations.

Jensen’s inequality, a consequence of nonlinear averaging, is another framework ecologists have used to predict the effects of variability on nonlinear processes [18,38–41]. Like many ecological processes and outcomes, disease risk tends to vary nonlinearly with temperature [12,42]. When there is temperature variability, nonlinearities can produce unintuitive species’ responses to fluctuating temperatures and can affect their responses independently of a changing mean [18]. Previous research investigating the effect of nonlinear averaging on nonlinear ecological traits have done so by integrating trait TPCs over a frequency distribution of environmental temperatures [18,38]. Applying nonlinear averaging to TPCs yields predictable effects for how organisms or systems should respond to thermal variability as this can be determined by a TPC’s local curvature. Here, averaging over the concave-up (accelerating) portion of a TPC should increase performance in response to thermal variability, whereas averaging over the concave-down (decelerating) portion of the TPC (e.g., the thermal optimum) should decrease performance. Though empirical examples are limited for species interactions, examples consistent with Jensen’s inequality include impacts of temperature variability on population growth [19,43,44], development [45–47], survival [45,46], and parasite replication [45].

Thus, mechanistic transmission models and nonlinear averaging are promising frameworks that both use TPCs to make predictions about ecological and epidemiological responses in variable environments. However, short-term temperature variation can complicate their use because of the effects of thermal acclimation. Thermal acclimation refers to changes in host and parasite TPCs that result from short-term, reversible physiological responses to changing thermal conditions [11,16,48,49]. The ability of an organism to acclimate, and the influence this has on performance, is impacted by a myriad of factors, including the organism’s thermal history [15,17,49], the frequency and magnitude of fluctuations [2,23], and the presence of species interactions [49]. On short timescales, these factors may change key characteristics of TPCs [15,48]. As a result, conventional TPCs, typically quantified under constant environmental conditions, may not capture complex responses to fluctuating temperatures. This poses problems for their utility in predictive models [14,16]. In host–parasite systems, differential acclimation responses may also alter theoretical predictions. Metabolic scaling rules suggest that parasites should gain an advantage over their hosts when there are unpredictable temperature changes, due to their smaller body size and fewer processes adjusting to thermal changes [2,3,11,35]. Acclimation to temperature variability may therefore alter the net outcome of host–parasite interactions by providing parasites with the upper hand (i.e., temperature variability hypothesis, [2,3,11,26]).

Using a *Daphnia magna-Ordospora colligata* host–parasite system, the goal of this study is to theoretically and empirically compare long-term disease dynamics, in the form of endemic infection prevalence, in constant and variable thermal environments. Endemic infection prevalence is defined here as the stationary distribution characterizing equilibrium conditions of the disease and represents the phase of the epidemic where the dynamics of the host–parasite system have stabilized. The *Daphnia*-parasite system is a naturally occurring environmentally transmitted disease system whose temperature-dependencies are well characterized [23,33,36,37]. Disease transmission occurs when infectious spores are passively ingested by grazing *Daphnia*, where they can infect gut epithelial cells and replicate into a cluster of spores. Infected epithelial cells eventually burst, freeing spores from a cluster to infect nearby gut cells in the same host or to be released into the water column to infect new hosts [50].

**Fig 2:**
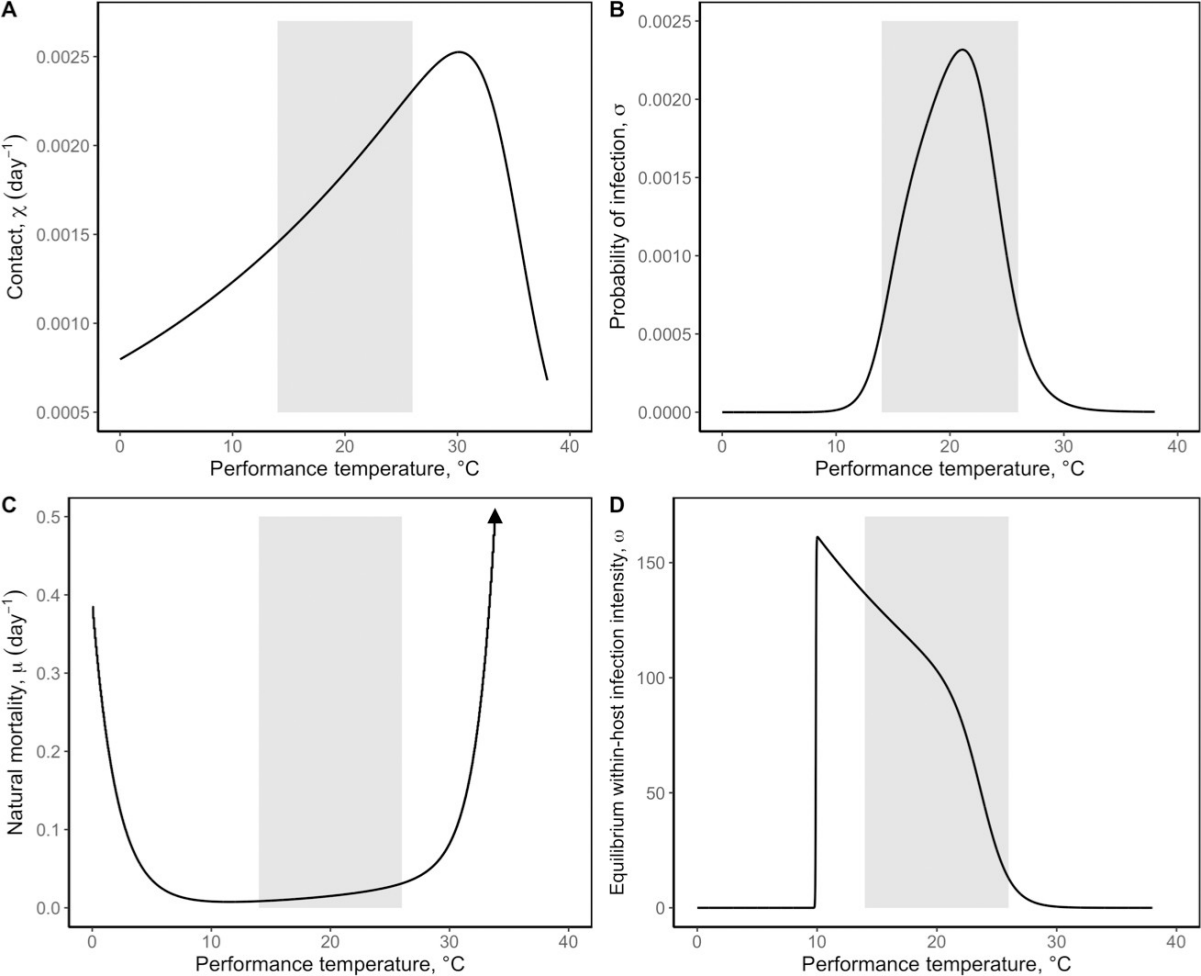
TPCs from MTE sub-models. TPCs were used to parameterize the model (Eqs 1.1–1.4). Rates are defined as daily rates. The **(A)** contact rate between hosts and environmental spores, *χ(T)*, is modeled in Eq S2.1. The **(B)** probability of infection, *σ(T)*, and the **(C)** natural mortality rate, *μ(T)*, are not directly modeled by MTE, but are derived from underlying MTE sub-models (Eq S2.2 and Eq S2.4–S2.6, respectively). **(D)** Equilibrium within-host infection intensity, *ω*(*T*), is estimated by MTE (Eq S2.3). Hosts that become infected are assumed to carry *ω*(*T*) spore clusters. *ω*(*T*) is used to derive the parasite-induced mortality rate, *α*(*T*), and the spore shedding rate of infected hosts, *λ*(*T*), across temperature. The shaded gray areas represent the +/− 6 range around T_opt_ = 20°C, where temperature fluctuations defined by Eq S1 could occur during the experiment. We accounted for this restriction in thermal fluctuations in theoretical predictions at the thermal optimum depicted in Fig 4. The data underlying this figure was generated by the MTE sub-models defined in Eq S2.1–S2.6 and parameterization of these equations is found in S2 Table. MTE, metabolic theory of ecology; TPC, thermal performance curve.

**Fig 3:**
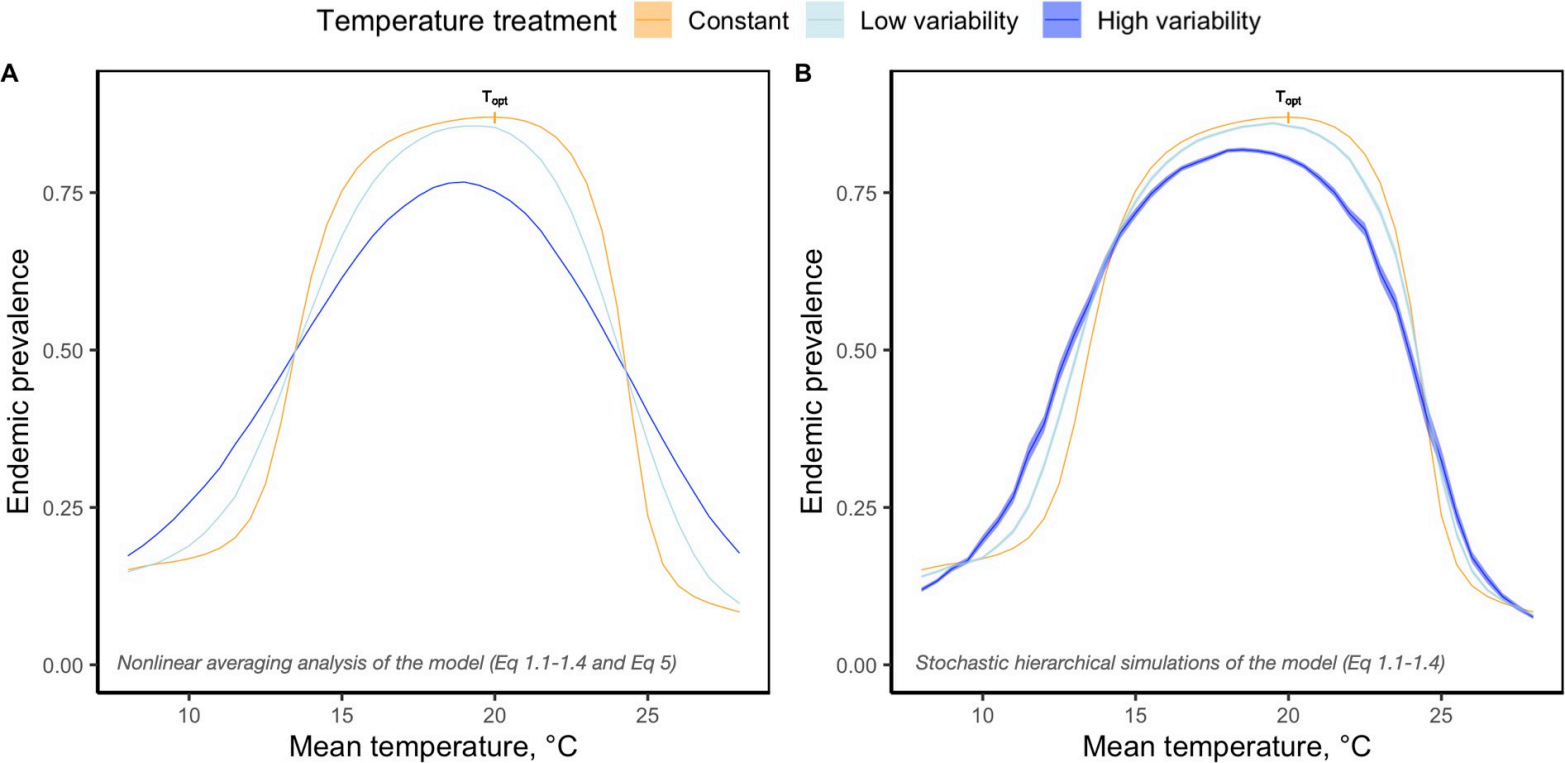
Predicted endemic prevalence thermal response curves in the *Daphnia*-parasite system. Panels **(A)** and **(B)** both compare endemic prevalence in constant (yellow curve) and variable (blue curves) environments along a range of mean temperatures (°C). The yellow curves in **(A)** and **(B)** are the same curves—they are numerically generated using deterministic simulations of the model (Eqs 1.1–1.4) for constant environmental conditions. The blue curves denote theoretical predictions generated under high temporal variance in temperature (dark blue, sd* = 3.*3*) and low temporal variance in temperature (light blue, sd* = 1.*7*). The variable temperature treatment thermal response curves in **(A)** are generated by nonlinear averaging (Eq 5) over the deterministic prediction of endemic prevalence from the model (Eqs 1.1–1.4). In panel **(B)**, the thermal response curves represent predictions numerically derived from stochastic hierarchical simulations of the model (Eqs 1.1–1.4). While **(A)** nonlinear averaging and **(B)** hierarchical stochastic simulations generate qualitatively similar predictions of endemic prevalence across temperatures, quantitative differences emerge in the predicted magnitude of effect of temperature variability across mean temperatures. The data underlying this figure is generated by Eqs S2.1–S2.6, 1.1–1.4, and Eq 5.

**Fig 4:**
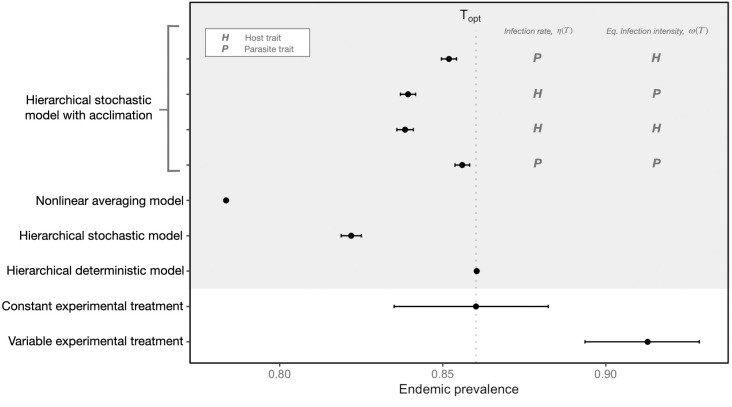
Comparison of model predictions (gray area) and experimental observations (white area) of endemic prevalence at the thermal optimum, T_opt_ = 20°C. Estimates under constant environmental conditions fall on the vertical dashed line, representing endemic prevalence at T_opt_. We used the high temperature variability sub-treatment (sd* = 3.3) to generate endemic prevalence predictions using nonlinear averaging (Eq 5), stochastic simulations of the model (Eqs 1.1–1.4), and stochastic simulations with acclimation (Eqs 1.1–1.4 and 2–4). Temperature fluctuations were modeled using an autocorrelated temperature model (S1) and were restricted to occur within a +/− 6 range around 20°C. The acclimation models represent conditions where time to host acclimation is fast (*ψ*_*H*_ = 6 days) and the effect of beneficial acclimation is strong (slope = 0.4, Eq 4). Additionally, the results of the acclimation models depicted in this figure differ in whether they assume the infection rate, *η*(*T*) (Eq S2.2 and Fig 2B), and equilibrium within-host parasite intensity, *ω*(*T*) (Eq S2.3 and Fig 2D), acclimate as a host trait, *H*, or as a parasite trait, *P* (S2 Table). All versions of our model simulations predict that temperature variability should suppress endemic prevalence and therefore shift left of the dashed line. Our endemic phase experimental observations illustrate the opposite effect and show that temperature variability leads to higher endemic disease prevalence. Uncertainty around model predictions and experimental results represent 95% credible intervals. The data in the gray portion of this figure is generated by Eqs S2.1–S2.6 and 1–5. Parameterization of these models is found in Tables 1, S2, and S3. The experimental data depicted in the white portion of this figure can be found in S1 Data.

We begin this paper with a mechanistic transmission model (Eqs 1.1–1.4, [33]) parameterized with TPCs from MTE sub-models (Eq S2.1–S2.6, Fig 2) for this host–parasite system [33,36,37]. The structure of this model is hierarchical because the temperature model (Eq S1) and the MTE models (Eq S2.1–S2.6) are nested within the transmission model (Fig 1). To numerically generate predictions of endemic infection prevalence in constant thermal conditions, the model was analyzed using deterministic simulations across a broad temperature range. For variable environmental conditions, the model was first analyzed by nonlinear averaging the deterministic prediction of endemic prevalence from the model over a probability distribution of temperatures (Eqs 1.1–1.4 and 5, Fig 1). We also analyzed the mechanistic transmission model using hierarchical stochastic simulations of the model where temperature fluctuations directly affect the interaction among TPCs parameterizing the model (Eqs 1.1–1.4, Fig 1). Both frameworks predict that short-term temperature variability on a daily timescale should suppress endemic infection prevalence at the pathogen’s thermal optimum, T_opt_ = 20°C (Figs 3 and 4). We experimentally tested that prediction using replicated epidemics in populations of the host–parasite system from which we could obtain estimates of endemic infection prevalence (S4 Fig). Replicated *Daphnia* populations were held at a mean temperature of T_opt_ = 20°C and were assigned to a constant or variable temperature treatment, the latter of which each received a unique sequence of daily temperature changes defined by an autocorrelated temperature model (Eq S1). All populations were initially susceptible, and the disease was introduced at a low rate over the course of the experiment. Prevalence and infection burdens were tracked every third day over 228 days of the experiment.

**Fig 5:**
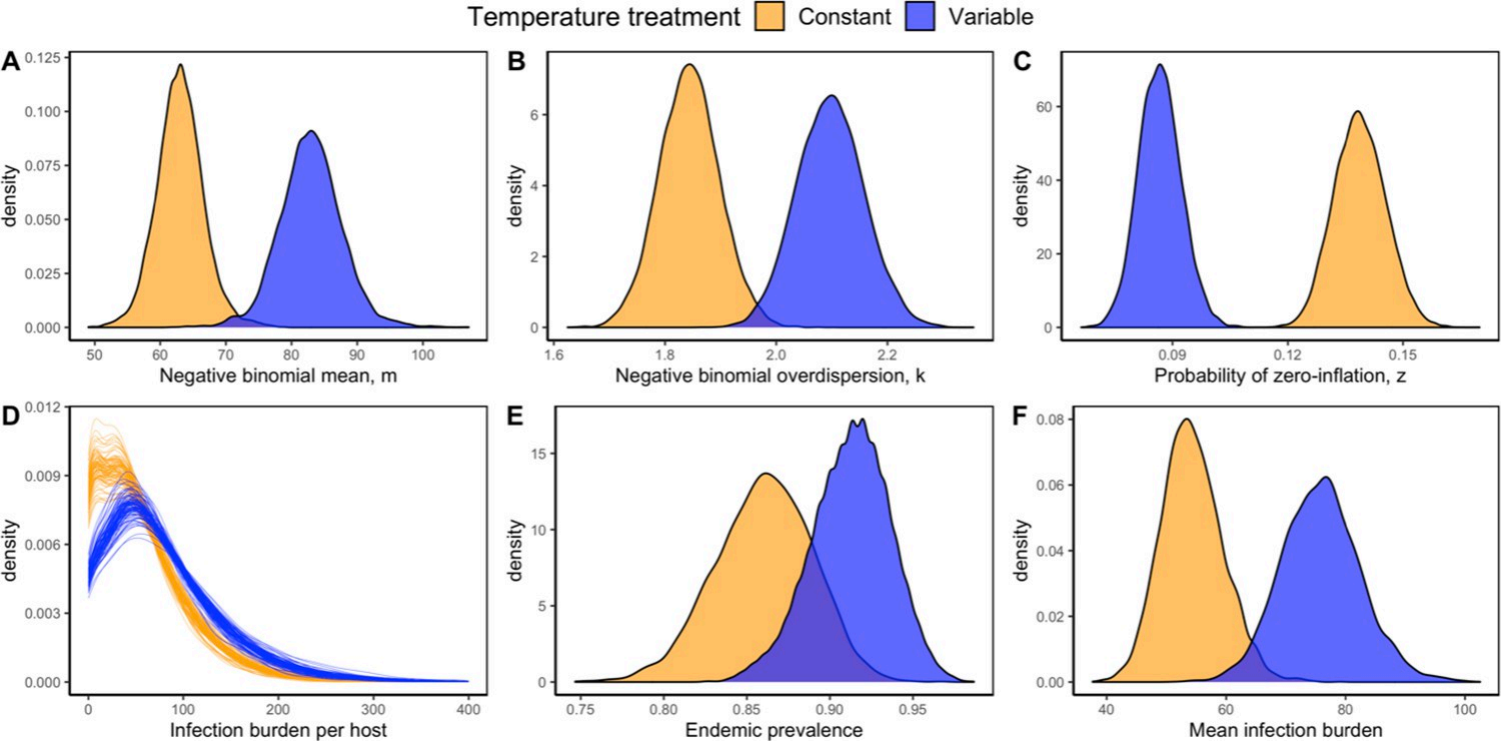
ZINB model fit to experimental infection burden data in constant (yellow) and variable (blue) temperature treatments. **(A)** and **(B)** illustrate the posteriors for the negative binomial component of the model fit, where the former is its mean, *m*, and the latter is the overdispersion parameter, *k*. Lower values of *k* indicate more variance in infection burden through a negative binomial process. **(C)** Illustrates the posteriors for the probability of zero-inflation, *z*, which describes the zero-inflated component of the model fit. Higher values of *z* indicate more variance in infection burden through a zero-inflated process. The ZINB model fit indicates that through both *k* and *z* temperature variability reduces the variance in infection burden across hosts. This can be seen in the overall ZINB model fit to infection burden **(D)** which illustrates that temperature variability flattens the ZINB distribution by shifting hosts towards the tail. **(E)** and **(F)** show distributions of endemic prevalence and mean infection burden, respectively, derived from the ZINB model fit. The shift in the distribution of infection burden as captured by the ZINB model is associated with an increase in **(E)** endemic prevalence and **(F)** mean infection burden in thermally variable environments. The data underlying this figure can be found in S1 Data. ZINB, zero-inflated negative binomial.

Our experimental observations were opposite our initial theoretical predictions; rather than decreasing endemic prevalence, short-term temperature variability increased endemic prevalence (Fig 4). The experimental data are qualitatively consistent with previously published work that suggest that short-term thermal fluctuations can increase disease due to the effects of delayed host acclimation (i.e., temperature variability hypothesis, [2,3]). However, incorporating acclimation into the mechanistic transmission model suggests that while it could be important in this system, additional empirical research on host–parasite acclimation responses to thermal fluctuations is required to adequately model its effect on disease dynamics (S3 Fig). Further analysis of the experimental data indicates that a shift in the distribution of parasites across the host population is associated with this positive effect of temperature variability on endemic prevalence (Fig 5). This implies that the effects of temperature variability on infectious disease systems may be mediated by biological mechanisms scaling from within- to among-host transmission dynamics.

## Results

### Theoretical predictions

#### Constant environmental conditions

*Deterministic hierarchical simulations of the model.* To evaluate how endemic infection prevalence changes across temperature in constant thermal environments, we used deterministic simulations of the *Daphnia-*parasite model (Eqs 1.1–1.4, [33]) parameterized with TPCs from MTE sub-models (S2.1–S2.6, Fig 2, [36,37]). At each constant mean temperature (8°C to 28°C), we simulated Eqs 1.1–1.4 forward in time and numerically calculated endemic infection prevalence as the proportion of infected hosts in the total population under equilibrium conditions. This allowed us to obtain an endemic prevalence thermal response curve across a range of constant temperatures and allowed us to identify the position of the thermal optimum (T_opt_ = 20°C) in constant environments (Fig 3, yellow curves).

#### Variable environmental conditions

*Nonlinear averaging.* We used nonlinear averaging (Eq 5) to analyze the model (Eqs 1.1–1.4) under variable environmental conditions. Nonlinear averaging is a mathematical calculation applied to nonlinear functions and has been used in ecology to predict the effect of temperature variability on thermal performance [18]. To obtain endemic prevalence predictions from nonlinear averaging, we first obtained a thermal response curve for endemic prevalence in constant environmental conditions using deterministic simulations of the model (see section above, Eqs 1.1–1.4, Fig 3, yellow curve). At each mean temperature (8°C to 28°C), we averaged over this curve using a probability distribution of temperatures obtained from the temperature model (Eq S1) under low (Eq S1, sd* = 1.*7*) and high (Eq S1, sd* = 3.*3*) temperature variability conditions (Eq 5).

The results of the model analyzed using nonlinear averaging (Eq 5) indicate that temperature variability affects endemic infection prevalence differently along different regions of the endemic prevalence thermal response curve. Our theoretical results are consistent with predictions expected under Jensen’s inequality (Fig 3A). Since the function is concave-down at T_opt_, nonlinear averaging and temperature variability predictably reduces endemic prevalence and shifts the peak of the curve towards lower temperatures. At low and high mean temperatures where the function is concave-up, temperature variability instead increases endemic prevalence and broadens the width of the curve. Both effects are larger when environments are more variable and combine to alter the overall shape of the endemic prevalence thermal response curve (Fig 3A).

*Stochastic hierarchical simulations of the model.* We also analyzed the model (Eqs 1.1–1.4) using hierarchical stochastic simulations to analyze how daily short-term thermal fluctuations affect endemic infection prevalence. This permutation of the model is different from analyzing the model using nonlinear averaging because the mechanistic interaction between the host and the parasite in thermally variable conditions is being explicitly modeled. Here, the model’s temperature-dependent parameters are allowed to vary with time, such that temperature fluctuations are hierarchically propagated through the mechanistic transmission model’s MTE-defined parameters (Fig 1). Replicate temperature sequences were generated for low and high temperature variability conditions across a range of mean temperatures (8°C to 28°C). At each mean temperature, the model was then simulated forward in time to numerically estimate endemic infection prevalence as the proportion of infected hosts in the total population under equilibrium conditions.

The results from the hierarchical stochastic model (Fig 3B) were qualitatively the same as those obtained from nonlinear averaging (Fig 3A). However, along the temperature range, applying nonlinear averaging to the deterministic equilibrium of the model predicts more extreme effects of temperature variability compared to the stochastic model. This result indicates that under thermally variable conditions, quantitative differences between nonlinear averaging methods (Eq 5) and mechanism-based modeling methods (Eqs 1.1–1.4) can emerge because of how temperature variability is assumed to operate on the host–parasite system.

*Stochastic hierarchical simulations of the model with acclimation.* Our theoretical predictions using the methods described above do not account for acclimation in the host–parasite system (Fig 1). We elaborated the model (Eqs 1.1–1.4) to test if acclimation responses to short-term thermal fluctuations could alter theoretical predictions of endemic prevalence and resolve the discrepancy with the empirical results (see next section, Fig 4). We conducted simulations of the model (Eqs 1.1–1.4) under a range of acclimation scenarios using a framework proposed by Rohr and colleagues ([11], Eqs 2–4). This framework assumes that acclimation responses of the host and the parasite scale with their body masses (Eq 2); short-term thermal fluctuations therefore provide parasites with an advantage over their hosts as they are capable of more rapidly acclimating to temperature changes [2,3,11]. We incorporated hypothetical acclimation responses by assuming that they occurred in the upper and lower thermal inactivation thresholds of trait TPCs defined by MTE sub-models (Eqs S2.1–S2.6).

In response to temperature variability, acclimation generally had the effect of increasing endemic prevalence relative to model predictions that do not explicitly account for acclimation in the host–parasite system (Figs 4 and S3). However, the magnitude of effect was never large enough to flip the direction of effect and produce a positive effect of temperature variability. This result implies that while host and parasite differences in acclimation ability can matter for transmission dynamics and endemic prevalence, it does not fully explain the positive effect of temperature variability we observed during the experiment (next section). Consequently, even when the hierarchical model (Eqs 1.1–1.4) accounts for hypothetical host and parasite acclimation responses, it is likely missing biological mechanisms that would correctly mediate the effects of short-term temperature fluctuations in this system.

### Experimental observations

To test our initial theoretical prediction that temperature variability suppresses endemic prevalence at the thermal optimum of the disease (Figs 3 and 4), we collected time series data for prevalence from 6 replicate populations in each of the constant and variable temperature treatments (S5 Fig). We truncated these data so that only observations of the endemic phase (the last 26 time points, corresponding to the last 78 days of the experiment) were retained. This cut-off was determined by conducting a sensitivity analysis to determine when the *Daphnia*-parasite system entered its stationary distribution representing equilibrium conditions (S10 Fig). To estimate endemic prevalence, we fit a logistic regression model using treatment as a fixed effect and replicate population as a random effect (Eq 6.1–6.2). Contrary to theoretical expectations, the experimental data clearly show that infection prevalence increased in response to the variable temperature treatment (Fig 4). Thus, the nonlinear averaging and hierarchical stochastic permutations of the model were both inadequate for capturing empirical transmission dynamics at the endemic phase. Furthermore, the addition of differences in host and parasite acclimation abilities still did not explain the direction and magnitude of effect we experimentally observed (Figs 4 and S3). These results suggest that while theory based on mechanistic disease transmission models and metabolic scaling theory are highly effective for capturing the effects of changes in mean temperature or slowly changing temperature [33], they are missing one or more key mechanisms for how temperature variability affects this model host–parasite system.

**Fig 6:**
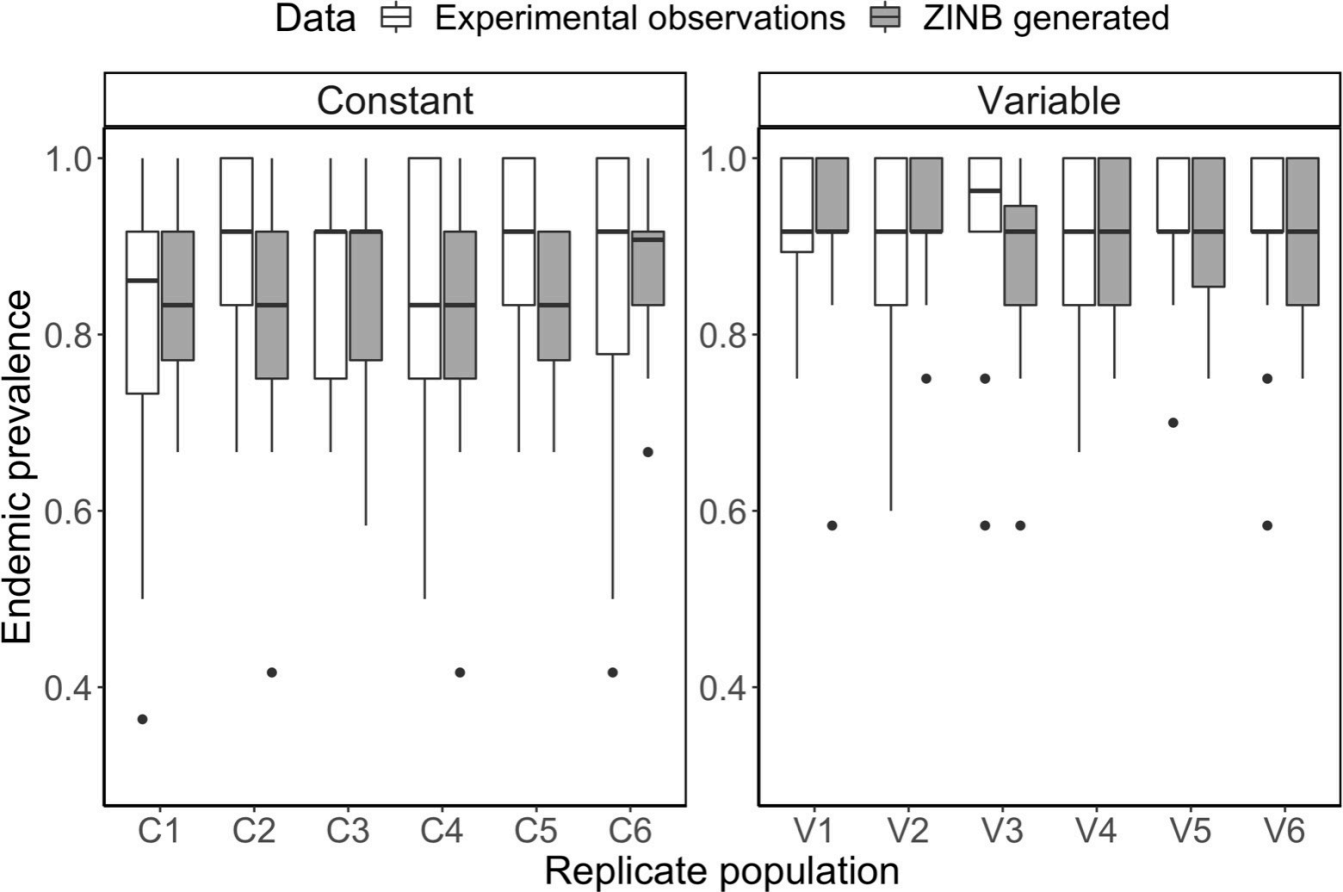
Comparison of ZINB model predictions of endemic prevalence (gray) and experimental observations of endemic prevalence (white) in each replicate population. ZINB model predictions of endemic prevalence were generated for each replicate population in the constant and variable temperature treatment. On the x-axis, each replicate population is indicated by a “C” or “V” denoting that it is from the constant or variable temperature treatment and a number that identifies the replicate population. The ZINB model predictions of endemic prevalence relatively match experimental observations of endemic prevalence in each replicate population thus indicating that the model well describes the data. The data underlying this figure can be found in S1 Data. ZINB, zero-inflated negative binomial.

We also collected infection burden data during the experiment, defined here as the number of parasite spore clusters infecting an individual host during the endemic phase (S6 Fig). We found that the distribution of infection burden was well described by a zero-inflated negative binomial (ZINB) model (Eq 7.1–7.2; Figs 5 and 6), which is an overdispersed distribution that distinguishes between 2 statistical processes generating infection burden in the host population. This is consistent with other research by Raffel and colleagues [2,26] who similarly found that ZINB distributions captured infection levels in host-*Bd* systems. Uninfected hosts with zero spore clusters arise first as a Bernoulli trial with probability of zero-inflation *z*. With probability *1 –z*, host infection burdens follow a negative binomial process with mean, *m*, and overdispersion parameter, *k*, and may also produce uninfected hosts with zero spore clusters. Lower values of *k* and higher values of *z* act to increase variation in infection burden by aggregating parasites to a subset of hosts. Together, *z*, *m*, and *k* represent statistical processes that produce variation in infection burden across hosts and can be estimated via maximum Bayesian methods (Eq 7.1–7.2).

Results from the ZINB model fit indicate that temperature variability alters the distribution of infection burden across hosts by inducing shifts in the parameters underlying the ZINB distribution (Fig 5A–5C). In particular, increased temperature variability acts to reduce the variance in host infection burden through both the overdispersion parameter, *k*, and the probability of zero-inflation, *z* (Fig 5B and 5C). This effect, caused by temperature variability, is associated with both an increase in the prevalence of infection (Fig 5E) and mean infection burden (Fig 5F). Furthermore, distributions of endemic prevalence derived from the ZINB model fit captured empirical observations of prevalence in each replicate population (Fig 6). This result implies that temperature variability’s effect on endemic prevalence is mediated by mechanisms that flatten the distribution of infection burden among hosts but does not identify the mechanisms.

## Discussion

Our experimental results are qualitatively consistent with the temperature variability hypothesis [3] and show that temperature variability increased disease both through elevated infection burdens and endemic prevalence (Figs 4 and 5). In contrast, analyzing a standard transmission model parameterized with MTE using hierarchical stochastic simulations and nonlinear averaging strongly predicts the opposite result, instead showing that temperature variability should suppress endemic prevalence at the thermal optimum (Fig 4). Incorporating hypothetical delayed host thermal acclimation responses in the model had the effect of increasing endemic prevalence relative to a model with no acclimation (Figs 4 and S3). The infection rate (or host susceptibility to infection) was particularly influential as it had the greatest effect on endemic prevalence when it was defined to acclimate according to the parasite (Figs 4 and S3). However, the effects of acclimation were not sufficient to flip the direction of effect of temperature variability on endemic prevalence, as temperature variability is shown to do in the experimental data (Figs 4 and S3). Consequently, unmodeled processes such as changes to within-host dynamics [2] that account for changes in the mean and variance of the distribution of infection burden (Fig 5) are likely needed to resolve the discrepancy between model predictions and empirical observations.

The hierarchical stochastic simulation of the model and nonlinear averaging, while producing the same qualitative predictions across temperature, generated quantitative differences in the predicted magnitude of effect of temperature variability (Fig 3). This may be partially explained by the timescale on which thermal fluctuations versus biological processes occur. Nonlinear averaging is typically used when temperature fluctuations occur on a shorter timescale compared to biological processes. In contrast, in our study, the timescale of daily thermal fluctuations is larger than that of some biological processes in the *Daphnia*-parasite system (e.g., the contact rate, the infection rate), and therefore could represent one source of divergence in the nonlinear averaging approximation relative to the stochastic model prediction. However, there are large timescale differences between daily thermal fluctuations and other biological processes in this *Daphnia*-parasite system. For example, at T_opt_ = 20°C, it takes 150 days to reach endemic prevalence (S5 Fig), at least 60 days for the parasite to reach equilibrium within the host [36], and as long as 100 days for *Daphnia* hosts to die [36]. In addition to timescale considerations, the nonlinear averaging approximation does not model the mechanistic interactions among temperature-dependent parameters in variable environments. It is simply a mathematical tool used to average over thermal response curves [18,19]—in our case, an endemic prevalence thermal response curve generated from an underlying mechanistic transmission model simulated under constant thermal conditions (Fig 3A). Nonlinear averaging therefore does not capture endemic prevalence as an emergent property of disease transmission dynamics in variable environments, as indicated by the mismatch between the endemic prevalence thermal response curves generated by the model analyzed using hierarchical stochastic simulations versus nonlinear averaging (Fig 3).

Rohr and Raffel’s [3] temperature variability hypothesis posits that while acclimation allows hosts and parasites to maintain their performance over time, parasites have an advantage because the ability to acclimate is driven by mass-specific differences in the metabolic rates of host and parasites (Eq 2, [11]). Our experimental results are consistent with this hypothesis as they demonstrate that unpredictable temperature fluctuations benefit the parasite by increasing both endemic prevalence and mean infection burden (Fig 5E and 5F). Our model analyzed under different acclimation scenarios (Eqs 1.1–1.4 and 2–4) suggests that changes in host susceptibility (i.e., the infectivity parameter) may be an important mechanism driving this response. Other experimental data support this explanation [2,26]—in the Cuban treefrog-*Bd* system, Raffel and colleagues [2] demonstrate that unacclimated frogs were less resistant to infection by *Bd* relative to acclimated frogs, and hypothesize that this is because of reduced efficacy of host immunity when the host is unable to anticipate temperature fluctuations in unpredictable thermal environments.

However, incorporating acclimation into the model (Eqs 1.1–1.4 and 2–4) only partially explains our experimental results (Figs 4 and S3). One reason this may occur is if the effect of temperature variability is mediated by acclimation effects scaling from within- to among-host dynamics. For example, as demonstrated in Raffel and colleagues [2], unpredictable temperature variability can increase host susceptibility at the within-host scale by raising parasite growth rates on frogs through lags in host immunity. This has the potential to influence disease transmission at the population-level (e.g., elevated shedding or infection rates) but would not be captured in classical mechanistic transmission models that do not explicitly define the interdependence of within- and among-host disease dynamics. A model [51] that defines this interdependence across scales is likely needed to evaluate the role of acclimation in increasing endemic prevalence in our experimental *Daphnia*-parasite system, particularly through shifts in within-host infection burden distributions (Fig 5).

Our initial models do not model acclimation responses to fluctuating temperatures such that the shape of TPCs is preserved over time (Figs 1 and 2). In contrast, modeling acclimation assumes that the shape of TPCs is dynamic because the upper and lower thermal thresholds of TPCs are linear functions of the host or parasite acclimation temperature ([11], Eqs 2–4, S2 Fig). Other research shows that acclimation may also manifest in other key metabolic traits, including the activation energy, *E*_*a*_, and may nonlinearly vary with acclimation temperature [48]. Importantly, defining acclimation in such traits points to different biological mechanisms shaping TPCs in fluctuating environments (e.g., acclimation in *E*_*a*_ could reflect changes in the amount of energy required to mount a reaction, whereas acclimation in thermal thresholds may reflect the degree of tolerance to temperature changes). However, acclimation within the framework of an among-host disease transmission model is unlikely to resolve the discrepancy between theory and experimental results because it does not address the distributional shifts in infection burden that underlie the shifts in prevalence (Fig 5).

We found that temperature variability flattens infection burden distributions by producing more hosts with high infection burdens (Fig 5D) and is associated with increasing endemic infection prevalence (Fig 5E). This raises 2 key questions: (1) how do hosts with high infection burdens emerge; and (2) what role do they play in driving disease transmission dynamics? To answer these questions, it is necessary to first understand how variation in infection burden, or parasite aggregation, emerges. Parasite aggregation is a fundamental property of host–macroparasite systems and is characterized by a subset of host individuals harboring a disproportionate number of parasites [52]. For host–microparasite systems, intraspecific variation in host traits (e.g., body size, age, behavior, immunity) can produce heterogeneity in host exposure and susceptibility to infection and can therefore generate variation in the number of parasites infecting a host. For example, relative to small hosts, hosts with large body sizes not only forage more (and thus face greater infection risks due to increased contact with the parasite) but also harbor more resource reserves that parasites can use for within-host replication. In this way, variation in host body size may underlie the emergence of microparasite aggregation by producing heterogeneity in among- and within-host susceptibility to infection. Demographic and environmental stochasticity are also important for generating variation in infection burden as the expression of disease traits (e.g., parasite replication, transmission) can be described as a draw from an underlying probability distribution [53].

Underpinning the distribution of infection burden (Fig 5D) is the balance between within-host processes that govern parasite growth and among-host processes that govern parasite transmission [54]. As a result, both parasite aggregation and prevalence at the host population-level emerge due to the interdependence of dynamics across scales. Our results show that the mechanisms that mediate the positive effect of temperature variability on endemic prevalence should increase the mean infection burden (Fig 5F), reduce the variance in infection burden (Fig 5B–5D), and increase the frequency of highly infected hosts (Fig 5D). The precise mechanisms by which temperature variability generates these patterns are unknown, but likely includes a combination of cross-scale factors that make hosts more prone to infection, such as, increased parasite replication rates [2] and increased host susceptibility to infection [2,3,25]. These effects cannot be captured by mechanistic transmission models because they typically only describe transmission dynamics at the among-host scale. A model that explicitly defines the biological interdependence of within-host processes (e.g., parasite replication, host resistance) and among-host processes (e.g., contact, infection, shedding rates) is therefore required to evaluate the role of short-term thermal fluctuations on disease dynamics [51]. Further, as many ecological characteristics result from the interdependence of processes across scales (e.g., diversity, coexistence, abundance, range [55–58]), our results indicate that applying nonlinear averaging directly to these characteristics without nesting temperature variability in an underlying cross-scale dynamical model can produce erroneous predictions (Fig 4).

As already discussed, acclimation effects can increase host susceptibility to infection due to the combined effects of lags in adjustments to host immunity and the ability of parasites to better maintain performance over time and over larger temperature ranges [2,3,11,26,59]. In our model, representing host susceptibility to infection are the component parts of the transmission rate (Eq 1.1–1.2) [37]. This is comprised of the contact rate between hosts and environmental spores, *χ(T)* (Fig 2A), and the probability of infection given host contact with an environmental spore, *σ(T)* (Fig 2B). *σ(T)* can be further broken down into the per-spore infection rate (*η*(*T*), Eq S2.2) and the spore gut residence time, the latter being the amount of time spent by a spore in a host before being passed back into the environment. The transmission rate also scales with the abundance of environmental spores, where higher environmental spore abundances create more opportunities for the host to encounter the parasite and possibly acquire an infection [37,60]. In variable environments, the product of these components elevates the transmission rate to increase endemic infection prevalence (Fig 5E) and reduce zero-inflation in the data (Fig 5C).

However, the transmission rate is composed of many different processes—the contact rate, the infection rate, and the gut residence time in this system—that have distinct nonlinear relationships with host body size and temperature [37] and therefore may interact differently with thermal variability to determine the overall biology of transmission and susceptibility. In a *Daphnia laevis*-*Metschnikowia bicuspidata* experimental system, short-term thermal fluctuations decreased transmission success of the disease by reducing contact rates with environmental parasites [61]. However, this effect can be overridden by other mechanisms that instead facilitate new infections such as effects of temperature variability on infectious environmental spores [17,26,62], within-host resistance or parasite growth [2,3,25,26], or their combination. Thus, precise experimental and theoretical work is required to disentangle which mechanisms are likely responsible for increasing susceptibility in variable environments through modifications to within- and among-host dynamics.

Another possible process that could increase the mean and reduce the variance in infection burden in variable environments is that unpredictable thermal variation makes hosts less resistant to infection by raising parasite growth rates on unacclimated hosts [2,26]. For chronic diseases, increasing the parasite replication rate across all hosts may reduce among-host variance (Fig 5B–5D) and increase mean abundance (Fig 5F) by allowing within-host parasite populations to reach equilibrium abundance more quickly. At the within-host scale, research has suggested that infection burdens emerge from the host immune response and the parasite competing for the same host resources [63,64]. Here, theoretical models have shown that infection burdens can increase if the parasite suppresses the ability of the host to mount an immune response through resource pre-emption, which assumes that parasites steal resources directly from the host’s energy reserves to then reduce the growth rate of immune cells and therefore undermine host resistance [63]. For *Daphnia*-parasite systems, infection loads tend to increase with the availability of food supplies to the host [64]. Because all replicate populations in our experiment received the same amount of food, this implies that temperature variability is possibly changing the interplay between the host immune response and the parasite to increase infection burdens, possibly through increased parasite replication rates or elevated within-host equilibrium abundances. This could occur, for example, if temperature variability provides parasites with a metabolic advantage over the host [2,11], increases host energy reserves by changing the host’s foraging ecology or shifting host population age or body size structures, or changes the way hosts allocate their resources among biological functions (e.g., towards dealing with thermal stress or acclimation rather than infection).

Heterogeneity in within-host dynamics produces variation in infection burden and may produce a subset of hosts that are more proficient, relative to the rest of the population, at spreading disease among hosts. For directly transmitted diseases like SARS and smallpox, research by Lloyd-Smith and colleagues [65] shows that models accounting for individual-level heterogeneity in infectiousness can generate superspreading individuals that produce rare but more explosive outbreaks and outperformed population-level models that typically average infectiousness and assume homogeneity. In our experimental system, the elevated frequency of highly infected individuals observed in the thermally variable treatment likely increases shedding rates and density-dependent transmission [60]. In this way, within-host dynamics that generate more highly infected hosts in the tail of the ZINB distribution may scale to enhance among-host transmission of the disease in thermally variable environments. Additionally, because *Ordospora* is relatively benign, hosts can tolerate the accumulation of high infection loads over their lifetime, which suggests a disproportionately important role for older, even dead (Eq 1.3–1.4), infected individuals in driving disease spread.

Overall, cross-scale interactions between hosts and parasites are likely important for shaping the thermal performance of a system. Previous research has shown that the temperature-dependence of host and parasite traits at within- and among-host scales can be described by TPCs from metabolic scaling theory [33,34,36,37]. Indeed, parasite replication and transmission are processes that depend on the interaction between parasite and host metabolism, body size, and temperature, the product of which can limit or enhance the impact of disease in host populations [2,25,66]. However, it remains unclear the extent to which species interactions, let alone temperature variability, alter metabolic scaling rules and whether system properties, like parasite aggregation and endemic prevalence, arise because of them [34].

A recent study by Kunze and colleagues [23], using the same *Daphnia*-parasite system, empirically illustrated that different patterns of variability (i.e., heatwaves, predictable diurnal fluctuations) can alter the shape of TPCs characterizing host and parasite traits in divergent ways. Near the thermal optimum, Kunze and colleagues [23] found that temperature fluctuations decreased mean infection burden in individual-level experiments of the *Daphnia*-parasite system. In contrast, mean infection burden increased in response to temperature variability around the thermal optimum in our population-level experiment (Fig 5F). This likely indicates that the type of short-term temperature variation can have unique effects on hosts and parasites and is essential for understanding how temperature variability should alter the net outcome of host–parasite systems. For example, at the within-host scale, predictable diurnal fluctuations may allow the host to better resist the disease through anticipation of a temperature change [2,23]—however, unpredictable temperature variation may allow greater exploitation of the host by the parasite given the parasite’s greater ability to acclimate to short-term temperature changes. This indicates that the impacts of temperature variability on host and parasite traits are context dependent and further experimental and theoretical work is needed to identify generalizable rules regarding how host and parasite traits respond to different types of short-term thermal fluctuations, how this translates to the emergence of TPCs, and how these effects scale to affect population-level properties like endemic prevalence.

Our experiment indicates that at the thermal optimum of the disease, trait TPCs are combining to increase, rather than decrease, endemic infection prevalence in variable environments. The results suggest that overlooking the effect of short-term thermal fluctuations on individual-level responses, particularly at the within-host scale, can lead to incorrect model predictions (Fig 4). Still, mechanistic models that incorporate the temperature-dependence of species’ physiology are invaluable for understanding how climate change will alter species interactions [11,12,33]. Together, the effects of plasticity, metabolic demands, nonlinearities, and the timescales and patterns of thermal change can produce complex ecological dynamics that are only revealed in the presence of climate variability.

## Materials and methods

### *Daphnia magna-Ordospora colligata* model system

*Daphnia magna* is a common zooplankton that is globally distributed in freshwater lakes and ponds. They are frequently infected with microsporidian parasites, including *Ordospora colligata*, used in this study. *Ordospora* is an endemic microparasite of *Daphnia* that is environmentally transmitted via spore stages suspended in the water column. Spores can be passively ingested by grazing *Daphnia*, where they can infect gut epithelial cells and replicate in the form of spore clusters. Each spore cluster can contain up to 64 individual spores that are released when infected epithelial cells burst. Spores released from a cluster can go on to infect nearby cells in the same host or they can be released back into the water column to infect new hosts. *Ordospora* is not a particularly virulent parasite and infections have been shown to be highly prevalent in natural populations of *Daphnia* [50]. Because *Daphnia* predominantly reproduce asexually, we focus on adult females throughout our modeling and experimental work, as juveniles and males are not the primary drivers of disease transmission [33]. The host and the parasite originate from Tvärminne Archipelago, Finland. *Daphnia* individuals in this study originate from the same clone (FI-OER3-3), from which the parasite strain (OC3) was isolated.

The *Daphnia*-*Ordospora* system is well suited for addressing the effect of temperature on diseases. This system fits the definition of disease models for microparasitic species. *Ordospora* is a small unicellular parasite that reproduces directly in the host and is directly transmitted among hosts. *Daphnia* and their microparasites also have short generation times and can be easily experimentally manipulated. This model host–parasite system is characterized across temperature [33,36,37]; this makes it possible to explore the theoretical implications of temperature variability on population-level disease responses in this system (e.g., endemic prevalence) and to experimentally test those predictions in a laboratory setting.

### Model

The main model (Eq 1.1–1.4) being analyzed in this paper is a mechanistic disease transmission model describing the among-host disease transmission dynamics of a *Daphnia*-parasite system [33]. It is defined with a set of ordinary differential equations tracking susceptible hosts (S), infected hosts (I), dead infected hosts that can still release environmental spores (D), and environmental spore stages (E).


$$\frac{dS}{dt}=\zeta_{S}+b(S+I)\left(1-\frac{S+I}{K}\right)-c\chi (T_{t})\sigma (T_{t})SE-\mu (T_{t})S-hS$$ (1.1)

$$\frac{dI}{dt}=\zeta_{I}+c\chi (T_{t})\sigma (T_{t})SE-(\mu (T_{t})+\alpha (T_{t}))I-hI$$ (1.2)

$$\frac{dD}{dt}=(\mu (T_{t})+\alpha (T_{t}))I-\theta D$$ (1.3)

$$\frac{dE}{dt}=\lambda (T_{t})I+\omega (T_{t})n\theta D-\gamma E$$ (1.4)

This model is parameterized with TPCs defined by MTE sub-models (Eq S2.1–S2.6, Fig 2, [36,37]). This enabled us to define temperature-dependent parameters as functions of the temperature at time step *t*. Throughout this paper, we refer to this model as a hierarchical model because of its nested structure—the temperature of the environment changes the numerical value of TPCs to directly affect disease transmission dynamics (Fig 1).

Predictions of endemic prevalence were obtained by analyzing the model in 4 different ways (Fig 1). First, for constant thermal environments, we used hierarchical deterministic simulations of the model where the numerical value of temperature-dependent parameters remains constant over time. Second, for variable thermal environments, we analyzed the model using nonlinear averaging, which averages the deterministic prediction of endemic prevalence from the hierarchical model (Eq 1.1–1.4) over a probability distribution of temperatures (Eq 5). The third way the model was analyzed used stochastic hierarchical simulations where temperature-dependent parameters vary with fluctuating temperatures. Finally, the model was analyzed using stochastic hierarchical simulations under different delayed host acclimation scenarios (S3 Table). For the hierarchical models, the model is simulated forward in time and endemic prevalence is numerically estimated as the proportion of infected hosts in the total host population under equilibrium conditions. The hierarchical structure of this model is relevant because it mediates the effect of temperature through mechanistic interactions among trait TPCs (Eq 1.1–1.4). Nonlinear averaging, on the other hand, uses an underlying hierarchical deterministic model to obtain an endemic prevalence thermal response curve, but ultimately assumes that the effect of temperature variability results from averaging over that nonlinear endemic prevalence thermal response function (see Nonlinear averaging section below).

In the model, the overall density of hosts is given by the sum of susceptible and infected adult females, which contribute to density-dependent logistic growth of the susceptible population (Eq 1.1). Susceptible individuals can also enter the population through an experimental immigration term, *ξ*_*S*_. Individuals can leave the susceptible state by natural mortality, *μ(T)*, an experimental harvesting term, *h*, or they can move into the infected state by transmission given that contact, *χ(T)*, with an environmental spore causes an infection with probability *σ(T)* (Eq 1.1). Infected individuals enter the infected state through disease transmission or an experimental immigration term, *ξ*_*I*_, and may leave the infected state through natural mortality, *μ*(*T*), parasite-induced mortality, *α*(*T*), or experimental harvesting, *h* (Eq 1.2). Once a host becomes infected, it is assumed to harbor the within-host equilibrium infection intensity, defined as the number of spore clusters infecting a host when the parasite is at equilibrium, *ω*(*T*) (Fig 2D and S2.3). Each spore cluster is assumed to contain 64 individual infectious spores, represented in the model by *n*.

Dead infected hosts also contribute to transmission dynamics by releasing environmental spore stages, the transmission stage of the parasite, until their corpse degrades. Eq 1.3 tracks the abundance of dead infected hosts over time as the sum of infected individuals who die due to natural mortality, *μ(T)*, and parasite-induced mortality, *α(T)*, minus the corpse degradation rate, *θ*, of dead infected hosts. In Eq 1.4, dead infected hosts shed environmental spores at rate *ω*(*T*)*nθ*. Infected dead hosts contain the equilibrium intensity of parasites, *ω*(*T*), defined as the equilibrium number of spore clusters. *n* converts *ω*(*T*) into the number of environmental spores shed from each spore cluster. Dead infected hosts shed environmental spores until their corpse degrades at rate *θ*. The change in the abundance of environmental spores at each time step is given in Eq 1.4 and is equal to the sum of spores shed infected hosts, *λ*(*T*), and dead infected hosts, *ω*(*T*)*nθ*, and spores lost through experimental removal, *γ*. We chose to model *γ* as the experimental removal of spores because natural environmental spore mortality is assumed to be negligible for this system.

The term *c* that appears in the disease transmission component of the model scales the predictions of endemic prevalence to observations of endemic prevalence at the constant treatment thermal optimum, T_opt_ = 20°C (Fig 4, vertical dashed line). The estimation of other model parameters was done using individual-level experiments in *Daphnia* and the model was analyzed using population-level quantities [33,36,37]. However, without *c*, this overestimates predictions of endemic prevalence coming from the model, perhaps due to effects of skewed among-host distribution of infection burden or other scaling processes.

### Temperature model

A temperature model (Eq S1), defined by a temporal autoregressive model, described daily temperature changes that were propagated through the model’s temperature-dependent parameters (Table 1). For each day the temperature changed according to Eq S1, the model’s (Eq 1.1–1.4) temperature-dependent parameters were assigned new numerical values from the MTE sub-models (Eq S2.1–S2.6), thus allowing temperature-dependent parameters to be functions of time. Temperature changes were centered on a mean temperature (8°C to 28°C) and occurred as an autocorrelated process, where the temperature at the current time step is linearly dependent on the temperature at the previous time step and a normally distributed error term, *ϵ*_*t*_~*N*(0, *sd*). Temperature variability was simulated under conditions of low and high temporal variance in temperature by changing the standard deviation of Eq S1’s error term. For the low temperature variability treatment, the error term was equal to *ϵ*_*t*_~*N*(0, *sd* = 1), with the standard deviation over the entirety of the temperature time series equal to sd* = 1.*7*. For the high temperature variability treatment, the error term was equal to *ϵ*_*t*_~*N*(0, *sd* = 2), with the overall standard deviation equal to sd* = 3.*3*. At each mean temperature (8°C to 28°C), we generated 100 unique temperature sequences using Eq S1 to model the effect of temperature variability on disease spread (Eq 1.1–1.4).

**Table 1:** Parameter definitions for the model. Temperature-dependent parameters are defined across temperature using Scharpe–Schoolfield (SS) MTE sub-models (Fig 2) that were previously empirically and theoretically quantified by Kirk and colleagues [36,37] for the *Daphnia-Ordospora* host–parasite system we used in this study (Eq S2.1-S2.6).

Symbol	Definition	Value	Unit
**Temperature-independent parameters**			
ζ_S_	Input of susceptible hosts	3.14	day^-1^
ζ_I_	Input of infected hosts	0.86	day^-1^
*b*	Adult recruitment	1	day^-1^
*c*	Scaling coefficient	0.00935	
*h*	Harvesting rate	0.0266	day^-1^
*n*	Spores released from a burst spore cluster	64	
θ	Degradation rate	0.01	day^-1^
γ	Environmental spore mortality	0.025	day^-1^
*K*	Carrying capacity	150	
**Temperature-dependent parameters**			
χ	Contact rate	SS	day^-1^
σ	Probability of infection given contact	SS	
μ	Host natural mortality rate	SS	day^-1^
α	Parasite-induced mortality rate	SS	day^-1^
λ	Spore shedding rate from infected hosts	SS	day^-1^
ω	Equilibrium within-host infection intensity	SS	

### Temperature-dependency of the model

We used 5 SS MTE sub-models (Eq S2.1–S2.6) to capture the temperature-dependence of our model (Eq 1.1–1.4, Fig 2). These were previously empirically and theoretically validated for this *Daphnia*-parasite system by Kirk and colleagues [36,37]. SS MTE models offer a useful extension to MTE as they link the temperature-dependence of biochemical reaction rates to ecological rates [34,67]. This produces unimodal curves characteristic of TPCs, which are typically optimized at an intermediate temperature and minimized at the ends of an organism’s thermal breadth, where rate-controlling enzymes are deactivated. Nesting SS MTE sub-models in mechanistic transmission models therefore provides a more realistic examination of disease transmission dynamics because the interaction among nonlinear thermal relationships in host and parasite traits are captured in model outcomes. MTE sub-models thus allowed us to parameterize the host–parasite system across temperature (Fig 2) and enabled us to capture the effects of constant and variable environmental conditions on disease transmission dynamics. An in-depth discussion of the parameterization of these sub-models is found in Kirk and colleagues [36,37], though we provide a broad overview of how they fit into our modeling framework in the supplementary materials.

### Acclimation model

To explore the effects of thermal acclimation on endemic prevalence, we used the framework proposed by Rohr and colleagues [11]. This framework accounts for differences in the ability of hosts and parasites to acclimate to temperature changes using principles from MTE. MTE predicts that parasites should more rapidly acclimate to changing temperatures relative to their hosts due to being small in size [35], and according to the temperature variability hypothesis, this should confer an advantage to the parasite when there are short-term thermal fluctuations [2,3,26]. Differences in host and parasite acclimation rates are therefore assumed to be driven by mass-specific differences between the interacting organisms. This was accounted for in the model (Eq 1.1–1.4) by allowing the upper and lower thermal thresholds of trait TPCs (Eq S2.1–S2.6) to be functions of the acclimation temperature of the host or the parasite [11,48]. In this way, thermal acclimation alters the shape of host and parasite TPCs (S2 Fig) that underlie disease transmission dynamics in the model (Eq 1.1–1.4) and can alter the net dynamics of infectious disease systems.

We assumed that the number of days it takes for the host, *ψ*_*H*_, and the parasite, *ψ*_*P*_, to acclimate to a novel temperature scaled with mass according to Eq 2. Eq 2 predicts that parasites should acclimate faster than their hosts with a ratio proportional to the parasite-to-host mass ratio.


$$\psi \propto Mass^{\frac{1}{4}}e^{\frac{E}{kT}}$$ (2)

When temperatures vary over time, the acclimation temperature of the host, $T_{acc}^{H}$, and the parasite, $T_{acc}^{P}$, at each time point can be described as the weighted average of temperature over *ψ*_*H*_ and *ψ*_*P*_ days backwards in time (Eq 3.1–3.2).


$$T_{acc}^{H}(t)=\frac{2}{\psi_{H}}\int_{x=0}^{\psi_{H}}[1-\frac{x}{\psi_{H}}]\cdot T(t-x)dx$$ (3.1)

$$T_{acc}^{P}(t)=\frac{2}{\psi_{P}}\int_{x=0}^{\psi_{P}}[1-\frac{x}{\psi_{P}}]\cdot T(t-x)dx$$ (3.2)

For a given trait, we assumed that the upper and lower thermal thresholds of each TPC varied linearly with the acclimation temperature of the host, $T_{acc}^{H}$, and the parasite, $T_{acc}^{P}$. For example, the upper and lower thermal thresholds for the host mortality rate, $\upsilon ,{T_{H}}_{\upsilon }$ and ${T_{L}}_{\upsilon }$, can be described as a linear function of the host acclimation temperature, $T_{acc}^{H}$ (Eq 4.1 and 4.2, respectively). Eq 4.1–4.2 can be modified to define how all other temperature-dependent parameters vary with either the host or parasite acclimation temperatures.


$$T_{H_{\upsilon }}(T_{acc}^{H})=\frac{T_{H_{\upsilon }}(T_{1})-T_{H_{\upsilon }}(T_{2})}{T_{1}-T_{2}}\cdot [T_{acc}^{H}-T_{2}]+T_{H_{\upsilon }}(T_{2})$$ (4.1)

$$T_{L_{\upsilon }}(T_{acc}^{H})=\frac{T_{L_{\upsilon }}(T_{1})-T_{L_{\upsilon }}(T_{2})}{T_{1}-T_{2}}\cdot [T_{acc}^{H}-T_{2}]+T_{L_{\upsilon }}(T_{2})$$ (4.2)

Eq 4.1–4.2 can be interpreted as a sort of beneficial acclimation response wherein acclimated organisms have increased performance relative to unacclimated organisms [2,3,26,49]. Here, cold acclimated hosts and parasites will perform better at cool temperatures relative to warm acclimated hosts and parasites, while the converse is true for warm acclimated hosts and parasites (S2 Fig, [11,48,49]). Parameterizing Eq 4.1 and 4.2 requires measuring each parameter at 2 different acclimation temperature points, T1 and T2, and provides the slope of the linear relationship. Since we do not possess this data, we arbitrarily chose different values for the slope (0.1 and 0.4, S3 Table). The larger slope has the effect of enhancing hypothetical beneficial acclimation responses in the temperature thresholds of trait TPCs (S2 Fig). We nested Eqs 4.1 and 4.2 into each metabolic sub-model to allow the effects of beneficial acclimation to shift trait TPCs (S2 Fig) and therefore allow TPCs to be dynamic over time.

Eq 4 requires assigning temperature-dependent traits as a host trait or a parasite trait. This distinction, however, is not always clear cut. For example, it is evident that the host contact rate (i.e., the filtration rate) and the host natural mortality rate are host traits and therefore acclimate according to host metabolic processes. But traits like the infection rate, *η*(*T*), and within-host equilibrium infection intensity, *ω*(*T*), emerge from host and parasite biological processes combining and therefore do not distinctly belong to either the host or the parasite. It is therefore unclear when such traits should acclimate according to the host, to the parasite, or both. We addressed this issue by running versions of the model that allowed such traits to vary according to one of the host or the parasite (Figs 4 and S3).

### Model analysis

#### Constant environmental conditions

*Deterministic hierarchical simulations of the model.* At each mean temperature (8°C to 28°C), the model (Eq 1.1–1.4) was simulated under constant environmental conditions for *t =* 600 time steps (days). Since this is a deterministic model, the system eventually reaches an asymptote representing equilibrium conditions. At each mean temperature, we calculated endemic prevalence as the proportion of infected hosts in the total population from *t = 300* to *t = 600* to obtain the predicted endemic prevalence thermal response curve in constant thermal conditions (Fig 3, yellow curves). In Fig 4, we wanted to ensure that our theoretical predictions of endemic prevalence were being compared over the same time period as our experimental observations of endemic prevalence. We conducted a sensitivity analysis on the experimental data to determine the time point at which the endemic phase begins (S10 Fig, see Sensitivity analysis section). The results from the sensitivity analysis suggest that our experimental populations entered the endemic phase on day 150 of the experiment. We therefore used the subset of the time series data from Eq 1.1–1.4 corresponding to days 150 to 228 (the last day of the experiment) and calculated endemic infection prevalence in constant thermal conditions in that time period. The model was simulated in R using the *deSolve* package [68,69].

#### Variable environmental conditions

*Nonlinear averaging.* The first method we used to model the effect of short-term thermal fluctuations on endemic prevalence was to analyze the model (Eq 1.1–1.4) using nonlinear averaging. Because nonlinear thermal responses are common across many ecological systems, there has been increasing interest in understanding the extent to which the impacts of temperature variability may be explained by the effects of nonlinear averaging (see [18,21,41] for a breakdown of the role of variability, nonlinear averaging, and Jensen’s inequality on nonlinear ecological responses). Predictions of endemic prevalence from nonlinear averaging were compared to those from stochastic hierarchical model simulations (see next section), where predictions under variable environmental conditions arise instead because of the mechanistic interactions characterizing disease transmission dynamics.

To analyze Eq 1.1–1.4 using nonlinear averaging, we first used deterministic simulations of the hierarchical model to generate an endemic prevalence thermal response curve under constant environmental conditions (Fig 3, yellow curves). We refer to this curve as $w(\bar{T})$, which represents endemic prevalence across a range of constant mean temperatures, $\bar{T}$. When temperatures vary over time, mean endemic prevalence, $\bar{w(T)}$, can be calculated by averaging $w(\bar{T})$ over a probability distribution of environmental temperatures, *P* (Eq 5) [18,21].


$$\bar{w(T)}=\int [w(\bar{T})\cdot P(T=t)]dT$$ (5)

Notably, the effect of temperature variability on $w(\bar{T})$ is predictable based on this function’s local curvature. When endemic prevalence is an accelerating function of constant mean temperature (i.e., $w(\bar{T})$ is concave-up), temperature variability should increase endemic prevalence, such that $w(\bar{T})<\bar{w(T)}$. Conversely, when $w(\bar{T})$ is decelerating (i.e., concave-down), $w(\bar{T})>\bar{w(T)}$, such that temperature variability decreases endemic prevalence. At each mean temperature (8°C to 28°C), Eq 5 was numerically implemented by discretizing the probability distribution of temperatures for low and high temperature variability sub-treatments (Eq S3.1–S3.2). Around the thermal optimum of the disease, since the constant endemic prevalence thermal response curve, $w(\bar{T})$, is concave-down, the effect of temperature variability predictably decreases endemic infection prevalence (Fig 3A).

*Stochastic hierarchical simulations of the model.* The second method we used to analyze Eq 1.1–1.4 with temperature variability was to simulate this model as a stochastic hierarchical model. At each mean temperature (8°C to 28°C), we generated 100 replicate temperature time series where daily temperatures fluctuations (*t = 600* days) could occur according to low and high temperature variability sub-treatments (Eq S1). These temperature time series were nested in the model, so that for each day *t* the temperature changed, temperature-dependent parameters were assigned a new numerical value computed by MTE sub-models (Eq S2.1–S2.6). Note that the model (Eq 1.1–1.4) is evaluated without acclimation—therefore, TPCs derived from the MTE sub-models are fixed over time. The model was then simulated forward in time, and endemic prevalence was calculated as the proportion of infected hosts in the host population using the simulation data from *t = 300* to *t = 600*, with uncertainty across replicate simulations represented as 95% credible intervals (Fig 3B). For the model predictions in Fig 4, we followed the same steps, only this time calculating endemic prevalence between *t = 150* to *t = 228* (the last day of the experiment). This was done to ensure that predictions of endemic prevalence were being compared over the same time period as experimental observations of endemic prevalence. Additionally, the temperature time series we used for the experimental populations limited temperature fluctuations to occur between +/−6 range around T_opt_ = 20°C; this rule was also applied to the model’s prediction of endemic prevalence at T_opt_ (Figs 2 and 4).

*Stochastic hierarchical simulations of the model with acclimation.* The final method we used to analyze the model (Eq 1.1–1.4) incorporated host and parasite acclimation responses [11]. Our first 2 methods (nonlinear averaging and stochastic hierarchical model) predicted that temperature variability should reduce endemic prevalence (Figs 3 and 4). But this prediction was opposite to what we experimentally observed (Fig 4). Thus, we chose to analyze the model under a range of acclimation scenarios to determine whether acclimation could explain the discrepancy between experimental observations of endemic prevalence and theoretical predictions of endemic prevalence derived from the model analyzed without acclimation (Fig 4). The acclimation scenarios are explained in-depth in the supplementary (S3 Fig and S3 Table). In general, we analyzed the model under various assumptions. First, we imposed one of 3 host acclimation time treatments: fast, medium, slow. This introduces delayed host acclimation responses that correspond to the host taking *ψ*_*H*_ = 6, 12, or 18 days to acclimate relative to the parasite’s *ψ*_*P*_ = 1 day. Since the parasite only takes a single day to acclimate, it acclimates instantaneously to temperature changes, such that the parasites acclimation temperature is always equal to the environmental temperature at time *t*. Second, we assumed the slope of Eq 4.1–4.2 could be equal to 0.1 or 0.4, which corresponds to weak or strong beneficial acclimation responses. Finally, we allowed the infection rate, *η*(*T*), and the within-host equilibrium infection intensity, *ω*(*T*) (Fig 2D), to vary according to the number of days it takes for the host to acclimate, *ψ*_*H*_, or the number of days it takes for the parasite to acclimate, *ψ*_*P*_. Following these assumptions, the stochastic hierarchical model with acclimation was simulated forward in time for *t = 228* days. We then used the subset of the data corresponding to days 150 to 228 to compare theoretical predictions against the empirical observations of endemic prevalence.

### Experimental methods

The experiment was designed to validate our initial model predictions at T_opt_ = 20°C, generated by nonlinear averaging (Eqs 1.1–1.4 and 5) and stochastic hierarchical simulations of the model (Eq 1.1–1.4). Here, theory predicts temperature variability should reduce endemic prevalence (Figs 3 and 4). Two weeks prior to the beginning of the experiment, asexual *Daphnia* adults were acclimated in environmental chambers set at 20°C, then randomly assigned to one of 12 replicate populations in either the constant (*n* = 6) or variable (*n* = 6) temperature treatment. Each replicate population contained approximately 150 *Daphnia* hosts. In the variable treatment, each replicate received a unique sequence of daily temperature changes according to the high temperature variability sub-treatment (Eq S1 and S1 Fig). Here, we limited temperature fluctuations to occur within a range of +/−6 around 20°C due to experimental constraints. Note that theoretical predictions at the thermal optimum depicted in Fig 4 account for this experimentally imposed restriction in temperature fluctuations.

The epidemic was initiated by introducing 3 *Daphnia* from infected lab stocks to each replicate population. Every third day, we obtained a sample of 12 *Daphnia* from each replicate population and used a microscope to destructively check whether each sampled host was (1) infected and (2) how many spore clusters they were infected with. For each replicate population, prevalence at each time point was calculated as the proportion of infected *Daphnia* in a sample, while mean infection burden was calculated as the average number of spore clusters infecting the *Daphnia* sample. Repeating the sampling process over time allowed us to obtain experimental time series for infection prevalence and mean infection burden over the course of the epidemic (S5 Fig). On the same day, the 12 sampled hosts from each replicate population were replaced with 9 individuals from acclimated susceptible lab stocks and 3 individuals from acclimated infected lab stocks. This allowed us to minimize the effects of disturbance from harvesting on the population and allowed us to repeatedly introduce the disease at a low rate over the course of the experiment. The introduction of new susceptible and infected individuals in each replicate population is represented in the model (Eq 1.1–1.4) by immigration terms *ξ*_*S*_ and *ξ*_*I*_, respectively.

### Model fits to experimental data

The analysis of our experimental data was conducted at the endemic phase of the epidemic, where all replicate populations were assumed to be at their stationary distribution representing equilibrium conditions. We determined the beginning of the stationary distribution, or the day at which all replicate populations have entered the endemic phase, by conducting a sensitivity analysis (S10 Fig). The sensitivity analysis was performed by truncating the data at a series of candidate days for the beginning of the stationary distribution and fitting the logistic regression (Eqs 6.1–6.2) and ZINB (Eqs 7.1–7.2) models to each resulting dataset. We used the Markov chain Monte Carlo (MCMC) software JAGS to fit the models within a Bayesian framework via the *rjags* and *dclone* packages [69–72] and visually determined when the resulting posterior distributions were relatively steady in their estimations over time (S10 Fig). Based on the results of the sensitivity analysis, we chose day 150 as the beginning of the endemic phase for all replicate populations and analyzed the data for all subsequent time points. Trace plots for the logistic regression (Eqs 6.1–6.2) and ZINB (Eq 7.1–7.2) models are provided in the supplementary and indicate that the model is well fit (S7 and S8 Figs).

To estimate endemic prevalence, we fitted a logistic regression model to binary infection status data during the endemic phase (Fig 4). Infection status data refers to data that identifies individual hosts observed during the experiment as infected or uninfected. The likelihood function can be written as follows:

$$y_{nij}∼Bernoulli(\tau_{ij})$$ (6.1)

$$logit(\tau_{ij})=\beta_{T}T_{i}+\alpha_{P}P_{j}$$ (6.2)

where the probability individual *n*, from treatment *i*, and replicate population *j* is infected is a Bernoulli trial characterized by *τ*_*ij*_. *τ*_*ij*_ may also be interpreted as the probability of infection at the endemic phase. Estimating *τ*_*ij*_ requires a logit link function and is expressed using temperature treatment as a fixed effect, *β*_*T*_, and replicate population as a random effect, *α*_*P*_. Priors for the logistic regression model can be found in S4 Table.

Similarly, we fitted a ZINB model to host infection burden data during the endemic phase. Infection burden data is count data that counts the number of spore clusters infecting an individual host observed during the experiment. Uninfected hosts are infected with 0 spore clusters, whereas infected hosts are infected with more than 0 spore clusters. The ZINB model describes the emergence of infection burden in the data as a mixture between zero-inflated and negative binomial processes. The likelihood function takes the form

$$y_{nij}∼ZINB(z_{i},m_{ij},k_{i})$$ (7.1)

$$\log\left(m_{ij}\right)=\beta_{T}T_{i}+\alpha_{P}P_{j}$$ (7.2)

where the infection burden, *y*, of individual *n* from treatment *i* and replicate population *j* is a random realization from a ZINB distribution with parameters *z*_*i*_, *m*_*ij*_, and *k*_*i*_. *z* is the probability of zero-inflation of the zero-inflated component of the model, *k* is the overdispersion parameter of the negative binomial component of the model, and *m* is the mean of the negative binomial component of the model. *z* and *k* represent distinct statistical processes that produce variation in infection burden. *m* is expressed as a log-linear combination of the fixed effect, temperature treatment, and the random effect, replicate population. Priors can be found in S4 Table. The ZINB model fit was used to derive estimates of treatment- and population-level estimates of mean infection burden (Figs 5F and S9) and endemic prevalence (Figs 5E, 6, and S9).

## Supporting information

S1 Fig Experimental temperature time series for each replicate population in the temperature variability treatment. “V” indicates the population is from the variable temperature treatment and the number identifies the replicate population. The red horizontal line is the mean temperature over the course of the experiment for a given replicate population. Using Eq S1, experimental populations could experience temperature fluctuations between 14°C and 26°C around the thermal optimum, T_opt_ = 20°C. This rule was also implemented for theoretical predictions simulating experimental conditions at the thermal optimum (Fig 4). The temperature data underlying this figure is found in S2 Data. Summary statistics for this data is found in S1 Table.
(TIF)

S2 FigThermal performance curves incorporating acclimation in the thermal thresholds of temperature-dependent host and parasite traits.Hypothetical acclimation responses in TPCs for the **(A)** contact rate, **(B)** probability of infection, **(C)** host mortality rate, and **(D)** within-host equilibrium infection intensity were incorporated by allowing the upper and lower thermal thresholds of each to vary with the acclimation temperature of the host, $T_{acc}^{H}$, or the parasite, $T_{acc}^{P}$. Depicted in this figure are TPCs for when species are fully cold acclimated, intermediate temperature acclimated, or warm acclimated. The slope of Eq 4 indicates the strength of the effect of beneficial acclimation on the thermal thresholds, where a slope of 0.4 indicates strong beneficial acclimation effects and a slope of 0 is no acclimation effects. The interaction between the slope and the acclimation temperature shifts trait TPCs. Note that these TPCs are dynamic through time because the acclimation temperature fluctuates over time. Additionally, since hosts exhibit delayed acclimation, hosts will have thermal performance curves that are partially acclimated to the environmental temperature at time *t*.(TIF)

S3 Fig Acclimation model results at the thermal optimum. We analyzed the model with acclimation (Eqs 1.1–1.4 and 2–4) to assess whether delays in host acclimation could explain the discrepancy between our theoretical predictions and experimental observations of endemic prevalence. This figure is an extension of Fig 4 in the main text and includes the model results under all combinations of acclimation scenarios. These scenarios vary the model conditions for the time to host acclimation, *ψ*_*H*_, the strength of beneficial acclimation (slope), acclimation in the infection rate, *η*(*T*), and acclimation in within-host infection intensity, *ω*(*T*). Model outcomes in the same set (S3 Table) model the same time to host acclimation and beneficial acclimation treatments but differ in whether they assume the infection rate and within-host infection intensity acclimate according to the number of days it takes for the host or the parasite to acclimate. This is indicated in the figure by an *H* for host and a *P* for parasite. Results from the experiment, from the hierarchical stochastic model without acclimation, and from nonlinear averaging are also included. These results indicate that acclimation, while potentially important in this system, cannot explain the direction and magnitude of effect of temperature variability that we experimentally observed. The data in the gray portion of this figure is generated by Eqs S2.1–S2.6 and 1–5. Parameterization of these models is found in Tables 1, S2, and S3. The experimental data depicted in the white portion of this figure can be found in S1 Data.
(TIF)

S4 Fig Illustration of experimental design at thermal optimum of the disease. We followed the spread of disease in 12 *Daphnia* populations held at a mean temperature of 20°C for 228 days. Six replicate populations were assigned to the constant temperature treatment and 6 replicate populations were assigned to the variable temperature treatment. Individuals across replicate populations started out as susceptible (green). The epidemic was initiated by introducing 3 individuals from infected lab stocks (red) into each population. The same sampling protocol was repeated for all replicate populations every 3 days. This allowed us to collect time series for prevalence and mean infection burden over the course of the epidemic. Gray *Daphnia* indicates sampled individuals with an unknown infection status. The infection status of these individuals was verified in the lab under a microscope where we determined whether the individual was infected and how many spore clusters they were infected with. Variable temperature treatment populations were each placed in their own water bath (blue border) that allowed us to change the water temperature based on temperature fluctuations from Eq S1 in the high temperature variability sub-treatment. *Daphnia* silhouettes were obtained from phylopic.org.
(TIF)

S5 Fig Experimental time series data for infection prevalence and mean infection burden in constant and variable temperature treatments. Replicate time series of **(A)** infection prevalence and **(B)** mean infection burden across sampled hosts in constant (*n* = 6) and variable (*n* = 6) temperature treatments over time (days). Each constant treatment replicate time series is indicated by a yellow line, a “C”, and a number that identifies the population. Each variable treatment replicate time series is indicated by a blue line, a “V”, and a number that identifies the population. The gray shaded region represents the endemic phase (*n* = 26, corresponding to 78 days at the endemic phase), which was assumed to begin on day 150 of the experiment. The beginning of the endemic phase was determined by conducting a sensitivity analysis. The data underlying this figure can be found in S1 Data. Prevalence was calculated as the proportion of infected individuals in a sample on each observation day. Mean infection burden was calculated as the average number of spore clusters infecting an individual on each observation day.
(TIF)

S6 Fig Histograms of host infection burden during the endemic phase. Experimental data of host infection burden during the endemic phase in the constant (yellow) and variable (blue) temperature treatments. For each replicate population, observations of host infection burden at the endemic phase are pooled across days. Each constant treatment histogram is indicated in yellow, and populations are indicated by a “C” and a number that identifies the population. Each variable treatment histogram is indicated in blue, and populations are indicated by a “V” and a number that identifies the population. The data underlying this figure can be found in S1 Data.
(TIF)

S7 Fig Trace plots for zero-inflated negative binomial model fit. For each parameter being estimated in the ZINB model, plots in the left-hand column depict trace plots and plots in the right-hand column depict density plots for the fitted model. For all estimated parameters, the chains in the trace plot are well-mixed and suggest convergence. The mean of the negative binomial component of the model was estimated using treatment as a fixed effect (represented by *β*_*T*_ in Eq 7.2, b[T] (Constant) and b[T] (Variable)), and replicate population as a random effect, (represented as *α*_*P*_ in Eq 7.2. a[1]-a[6] are constant treatment replicates and a[7]-a[12] are variable treatment replicates). The overdispersion parameter, *k* ([k [1] constant, k [2] variable), and the probability of zero-inflation, *z* (theta [1] constant, theta [2] variable), were both estimated at the treatment level. The data underlying this figure can be found in S1 Data.
(PDF)

S8 Fig Trace plots for logistic regression model fit. For each parameter estimated in the logistic regression model, plots in the left-hand column depict trace plots and plots in the right-hand column depict density plots for the fitted model. The probability of being infected, given by *τ*, was estimated using temperature treatment as a fixed effect (bT [1] constant, bT [2] variable) and replicate population as a random effect (aP[1]-aP[6] constant, a[7]-a[12] variable). All estimated parameters converge as indicated by well-mixed chains in the model trace plots. The data underlying this figure can be found in S1 Data.
(PDF)

S9 Fig Comparison of endemic prevalence and mean infection burden for experimental observations, zero-inflated negative binomial model fit, and negative binomial model fit. Gray boxes are observed estimates from our experiment. White and black boxes are predicted data from the zero-inflated negative binomial (ZINB) and the negative binomial (NB) model fits to the experimental data, respectively. On the x-axis, each replicate population is indicated by a “C” or “V” denoting that it is from the constant or variable temperature treatment and a number that identifies the population. Notably, the negative binomial model fit overestimated endemic prevalence. The data underlying this figure can be found in S1 Data.
(TIF)

S10 Fig Sensitivity analysis. The ZINB and logistic regression models were re-fit to a series of candidate days for the first day of the endemic phase. We define the first day of the endemic phase as the first day that all replicate populations across constant and variable temperature treatments enter the stationary distribution representing equilibrium conditions of the disease. This was done to ensure that estimated posteriors were robust and that our results were not contingent on the chosen day for the first day of the endemic phase. The vertical gray line is the posterior estimate when the endemic phase starts at day 150, as we have assumed in the main text. The results of the sensitivity analysis confirm that our results are not contingent on when the endemic phase is assumed to begin. The data underlying this figure can be found in S1 Data.
(TIF)

S1 Table Mean and standard deviation of experimental temperatures. The mean and standard deviation of experimental temperatures were calculated for each replicate population over the entirety of the experiment and over the endemic phase. All populations were assumed to have entered the endemic phase by day 150 of the experiment. The data underlying this figure can be found in S2 Data.
(XLSX)

S2 Table Parameter definitions for MTE sub-models. The MTE sub-models were previously empirically and theoretically parameterized by Kirk and colleagues [36,37] for the *Daphnia*-parasite system. Note that the reference temperature is not the same for all parameters. Temperatures are reported in degrees Kelvin, K. Parameterization of these models can be found in S2 Table.
(XLSX)

S3 Table Summary of acclimation scenarios. We analyzed the transmission model (Eqs 1.1–1.4) under several acclimation scenarios (Eqs 2–4). These scenarios include various combinations of the time to host acclimation, *ψ*_*H*_, the strength of beneficial acclimation, and whether the infection rate, *η*(*T*), and equilibrium within-host infection intensity, *ω*(*T*), acclimates as a host trait or parasite trait. Each model version reflects different combinations of model assumptions. The table is organized such that predictions of endemic prevalence from the same set of models are simulated under the same model assumptions, with exception to whether the infection rate or the equilibrium within-host infection intensity acclimates according to the host or the parasite.
(XLSX)

S4 Table Priors for the logistic regression model, the zero-inflated negative binomial model, and the negative binomial model. (XLSX)

S5 Table Summary of model fits for the logistic regression model, the zero-inflated negative binomial model, and the negative binomial model. (XLSX)

S1 Data Experimental data for infection prevalence and host infection burden. (CSV)

S2 Data Temperature time series for experimental temperature variability treatment. (CSV)

S1 Text Supporting information. (DOCX)

## Abbreviations

MCMC: Markov chain Monte Carlo
MTE: metabolic theory of ecology
SS: Scharpe–Schoolfield
TPC: thermal performance curve
ZINB: zero-inflated negative binomial
