# Supplementary figures and images for: Short-term temperature fluctuations increase disease in a Daphnia-parasite infectious disease system
Source: PLoS Biol. 2023 Sep 8;21(9):e3002260. doi: 10.1371/journal.pbio.3002260 (PMC10491407; doi:10.1371/journal.pbio.3002260)

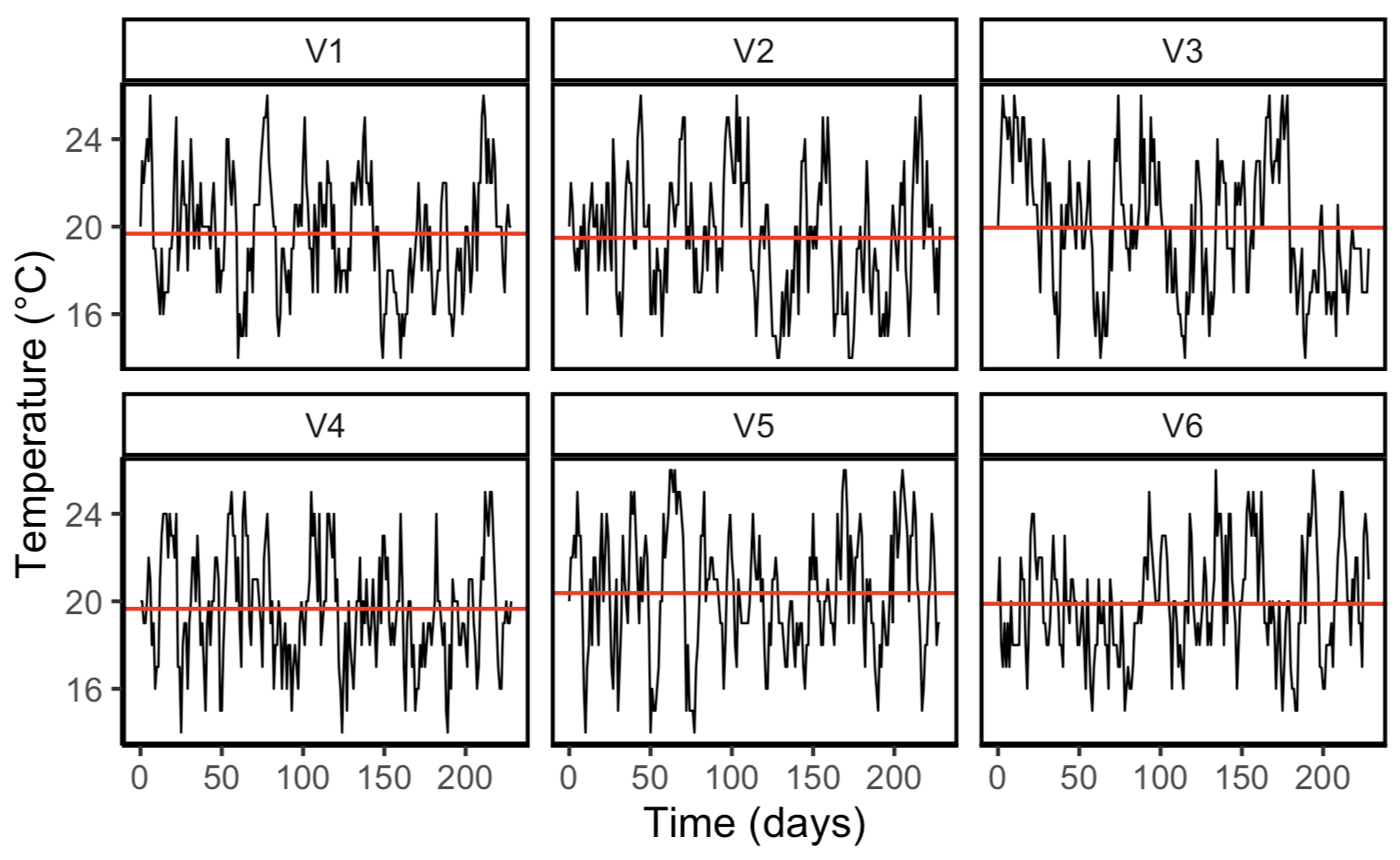

Supplement: S1 Fig — “V” indicates the population is from the variable temperature treatment and the number identifies the replicate population. The red horizontal line is the mean temperature over the course of the experiment for a given replicate population. Using Eq S1, experimental populations could experience temperature fluctuations between 14°C and 26°C around the thermal optimum, Topt = 20°C. This rule was also implemented for theoretical predictions simulating experimental conditions at the thermal optimum (Fig 4). The temperature data underlying this figure is found in S2 Data. Summary statistics for this data is found in S1 Table. (TIF) [file pbio.3002260.s001.tif]

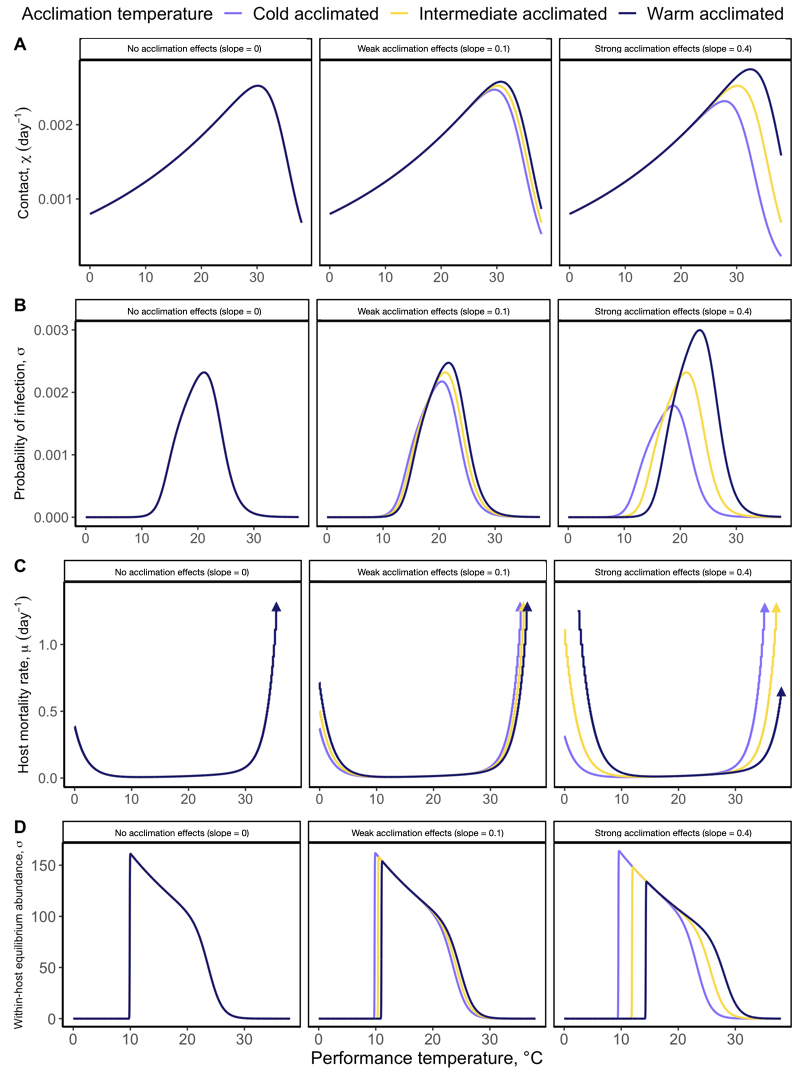

Supplement: S2 Fig — Hypothetical acclimation responses in TPCs for the (A) contact rate, (B) probability of infection, (C) host mortality rate, and (D) within-host equilibrium infection intensity were incorporated by allowing the upper and lower thermal thresholds of each to vary with the acclimation temperature of the host, TaccH, or the parasite, TaccP. Depicted in this figure are TPCs for when species are fully cold acclimated, intermediate temperature acclimated, or warm acclimated. The slope of Eq 4 indicates the strength of the effect of beneficial acclimation on the thermal thresholds, where a slope of 0.4 indicates strong beneficial acclimation effects and a slope of 0 is no acclimation effects. The interaction between the slope and the acclimation temperature shifts trait TPCs. Note that these TPCs are dynamic through time because the acclimation temperature fluctuates over time. Additionally, since hosts exhibit delayed acclimation, hosts will have thermal performance curves that are partially acclimated to the environmental temperature at time t. (TIF) [file pbio.3002260.s002.tif]

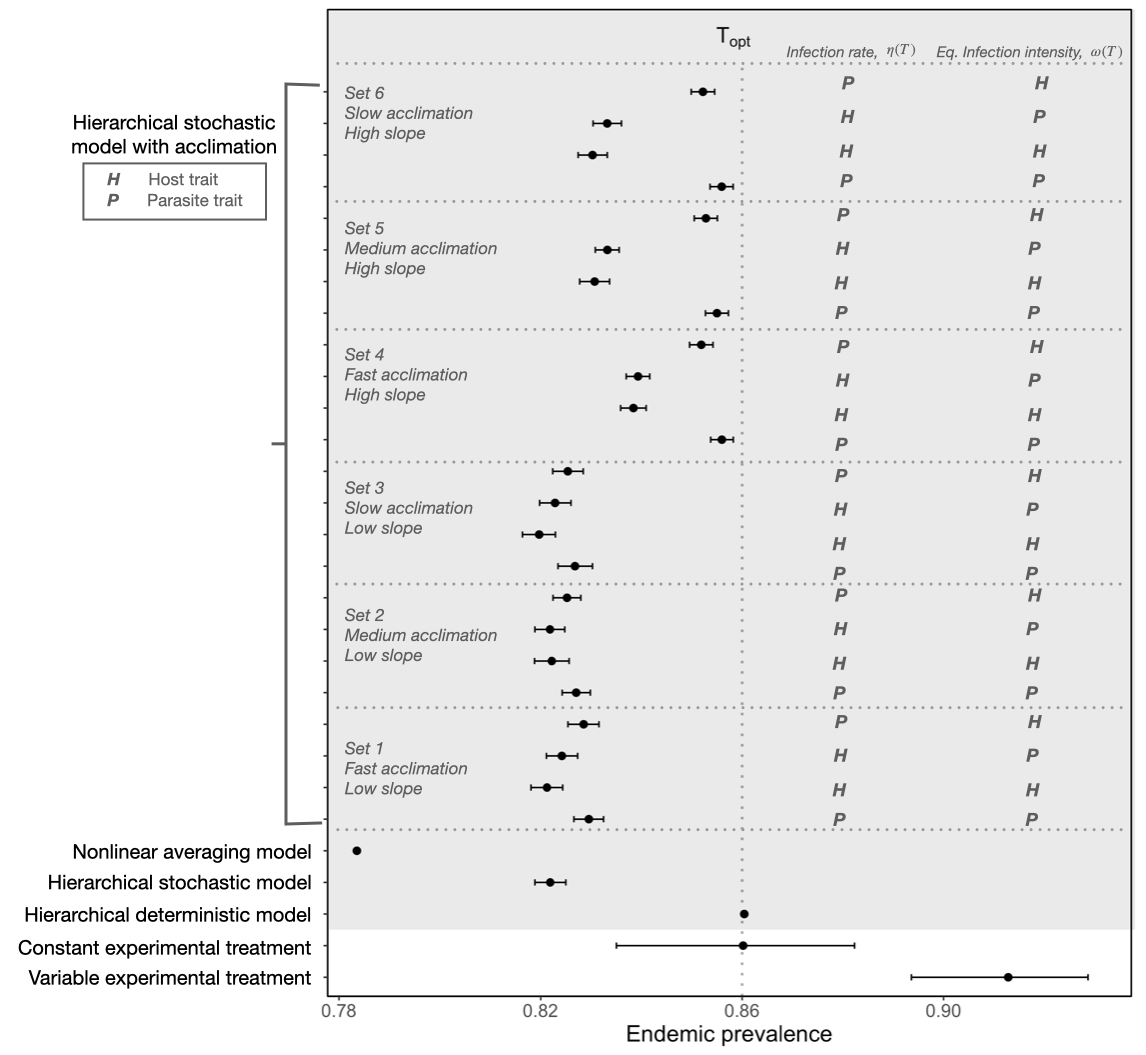

Supplement: S3 Fig — We analyzed the model with acclimation (Eqs 1.1–1.4 and 2–4) to assess whether delays in host acclimation could explain the discrepancy between our theoretical predictions and experimental observations of endemic prevalence. This figure is an extension of Fig 4 in the main text and includes the model results under all combinations of acclimation scenarios. These scenarios vary the model conditions for the time to host acclimation, ψH, the strength of beneficial acclimation (slope), acclimation in the infection rate, η(T), and acclimation in within-host infection intensity, ω(T). Model outcomes in the same set (S3 Table) model the same time to host acclimation and beneficial acclimation treatments but differ in whether they assume the infection rate and within-host infection intensity acclimate according to the number of days it takes for the host or the parasite to acclimate. This is indicated in the figure by an H for host and a P for parasite. Results from the experiment, from the hierarchical stochastic model without acclimation, and from nonlinear averaging are also included. These results indicate that acclimation, while potentially important in this system, cannot explain the direction and magnitude of effect of temperature variability that we experimentally observed. The data in the gray portion of this figure is generated by Eqs S2.1–S2.6 and 1–5. Parameterization of these models is found in Tables 1, S2, and S3. The experimental data depicted in the white portion of this figure can be found in S1 Data. (TIF) [file pbio.3002260.s003.tif]

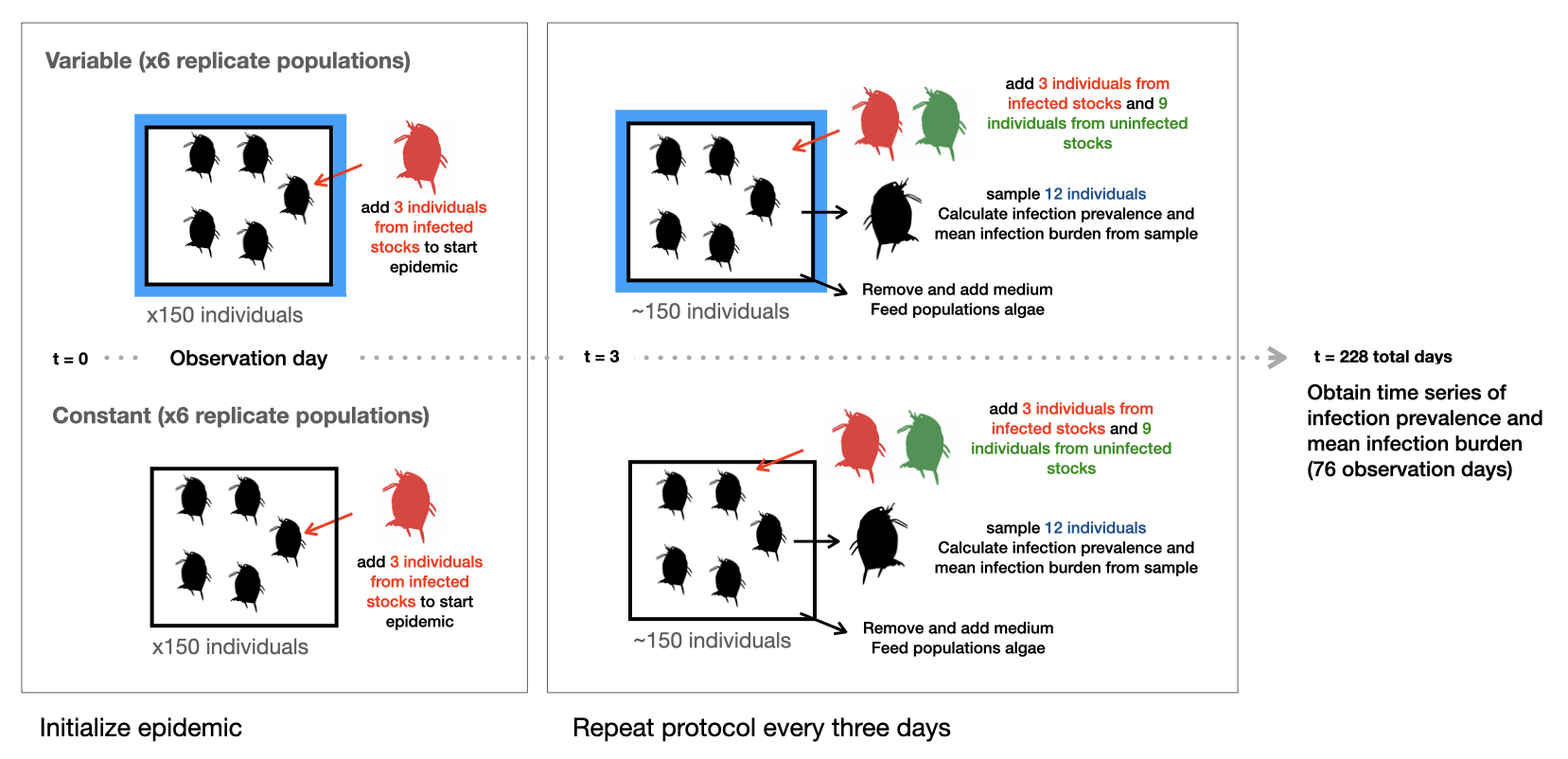

Supplement: S4 Fig — We followed the spread of disease in 12 Daphnia populations held at a mean temperature of 20°C for 228 days. Six replicate populations were assigned to the constant temperature treatment and 6 replicate populations were assigned to the variable temperature treatment. Individuals across replicate populations started out as susceptible (green). The epidemic was initiated by introducing 3 individuals from infected lab stocks (red) into each population. The same sampling protocol was repeated for all replicate populations every 3 days. This allowed us to collect time series for prevalence and mean infection burden over the course of the epidemic. Gray Daphnia indicates sampled individuals with an unknown infection status. The infection status of these individuals was verified in the lab under a microscope where we determined whether the individual was infected and how many spore clusters they were infected with. Variable temperature treatment populations were each placed in their own water bath (blue border) that allowed us to change the water temperature based on temperature fluctuations from Eq S1 in the high temperature variability sub-treatment. Daphnia silhouettes were obtained from phylopic.org. (TIF) [file pbio.3002260.s004.tif]

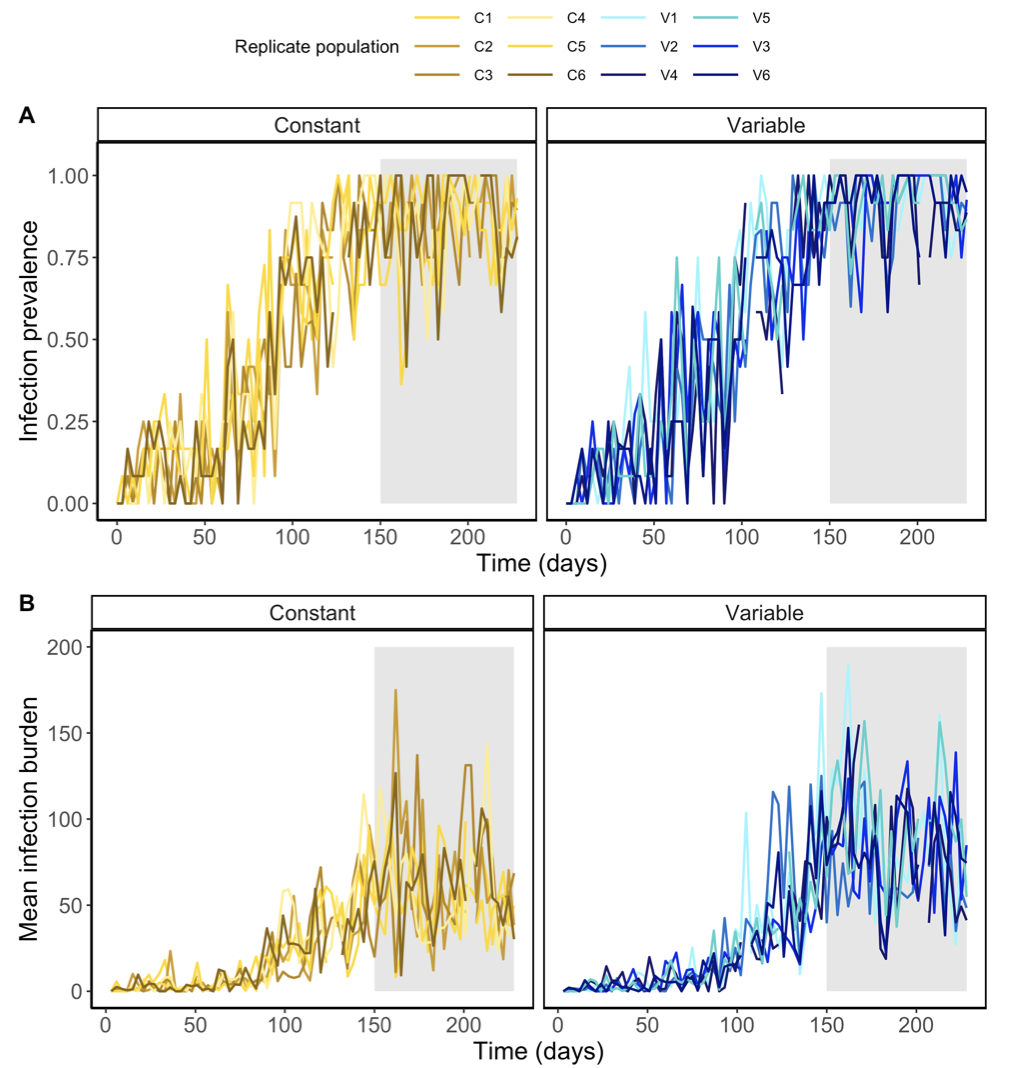

Supplement: S5 Fig — Replicate time series of (A) infection prevalence and (B) mean infection burden across sampled hosts in constant (n = 6) and variable (n = 6) temperature treatments over time (days). Each constant treatment replicate time series is indicated by a yellow line, a “C”, and a number that identifies the population. Each variable treatment replicate time series is indicated by a blue line, a “V”, and a number that identifies the population. The gray shaded region represents the endemic phase (n = 26, corresponding to 78 days at the endemic phase), which was assumed to begin on day 150 of the experiment. The beginning of the endemic phase was determined by conducting a sensitivity analysis. The data underlying this figure can be found in S1 Data. Prevalence was calculated as the proportion of infected individuals in a sample on each observation day. Mean infection burden was calculated as the average number of spore clusters infecting an individual on each observation day. (TIF) [file pbio.3002260.s005.tif]

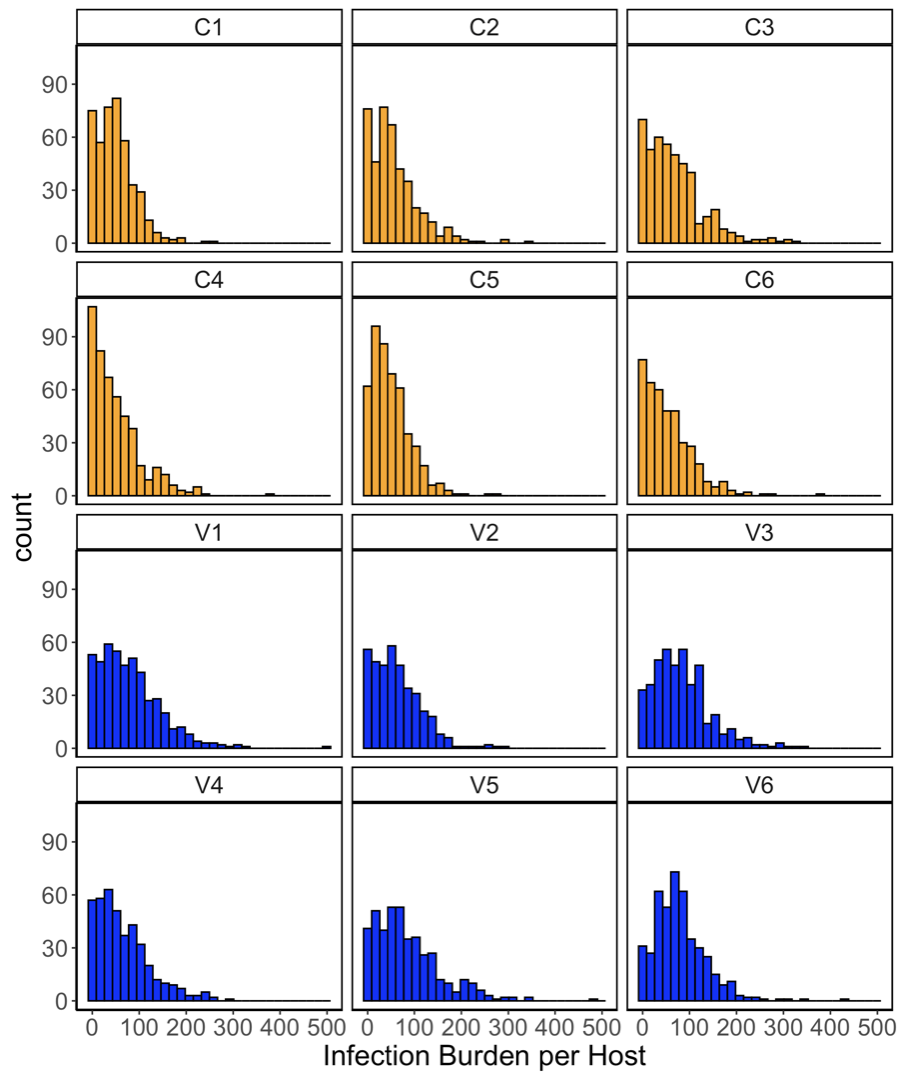

Supplement: S6 Fig — Experimental data of host infection burden during the endemic phase in the constant (yellow) and variable (blue) temperature treatments. For each replicate population, observations of host infection burden at the endemic phase are pooled across days. Each constant treatment histogram is indicated in yellow, and populations are indicated by a “C” and a number that identifies the population. Each variable treatment histogram is indicated in blue, and populations are indicated by a “V” and a number that identifies the population. The data underlying this figure can be found in S1 Data. (TIF) [file pbio.3002260.s006.tif]

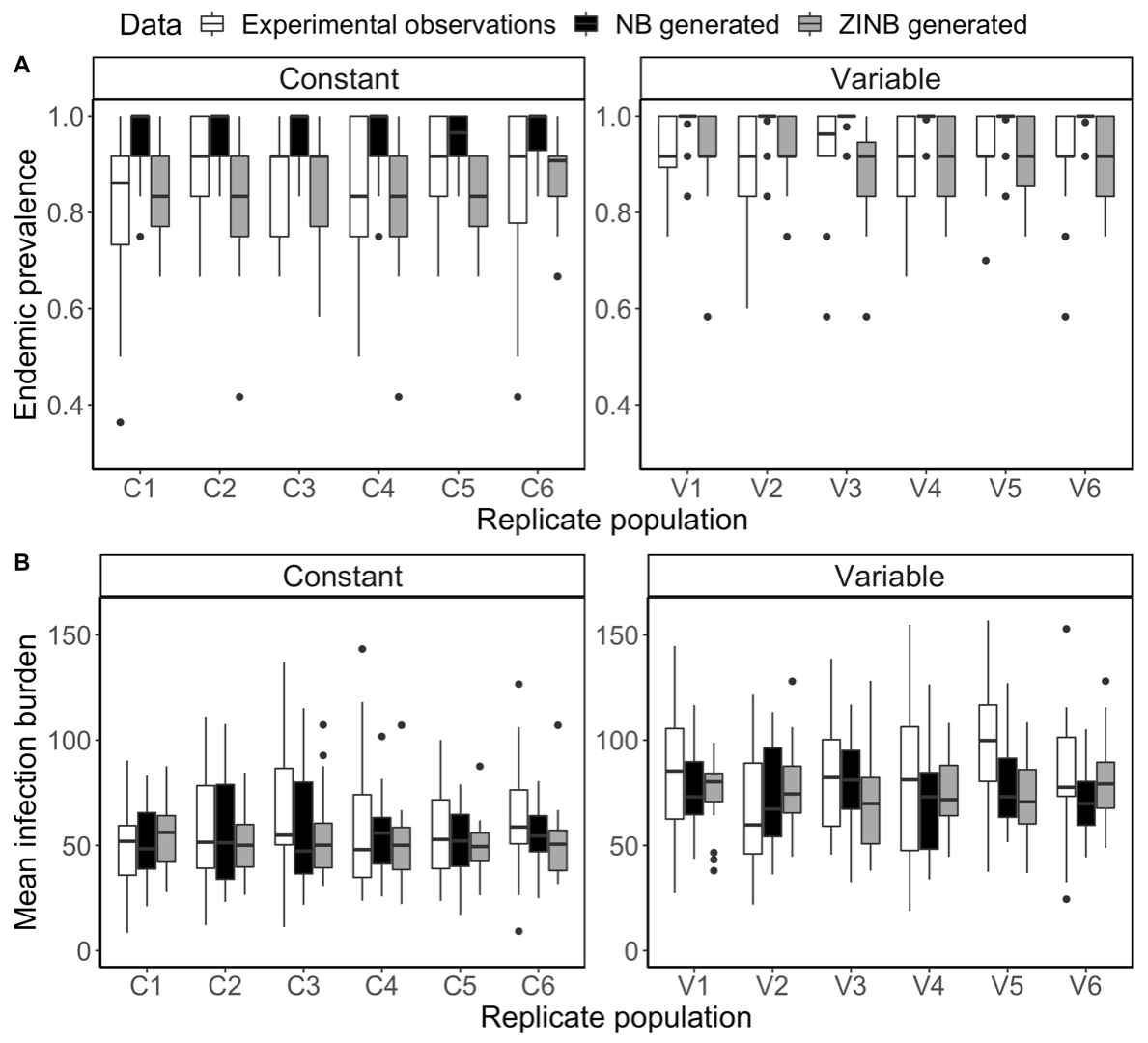

Supplement: S9 Fig — Gray boxes are observed estimates from our experiment. White and black boxes are predicted data from the zero-inflated negative binomial (ZINB) and the negative binomial (NB) model fits to the experimental data, respectively. On the x-axis, each replicate population is indicated by a “C” or “V” denoting that it is from the constant or variable temperature treatment and a number that identifies the population. Notably, the negative binomial model fit overestimated endemic prevalence. The data underlying this figure can be found in S1 Data. (TIF) [file pbio.3002260.s009.tif]

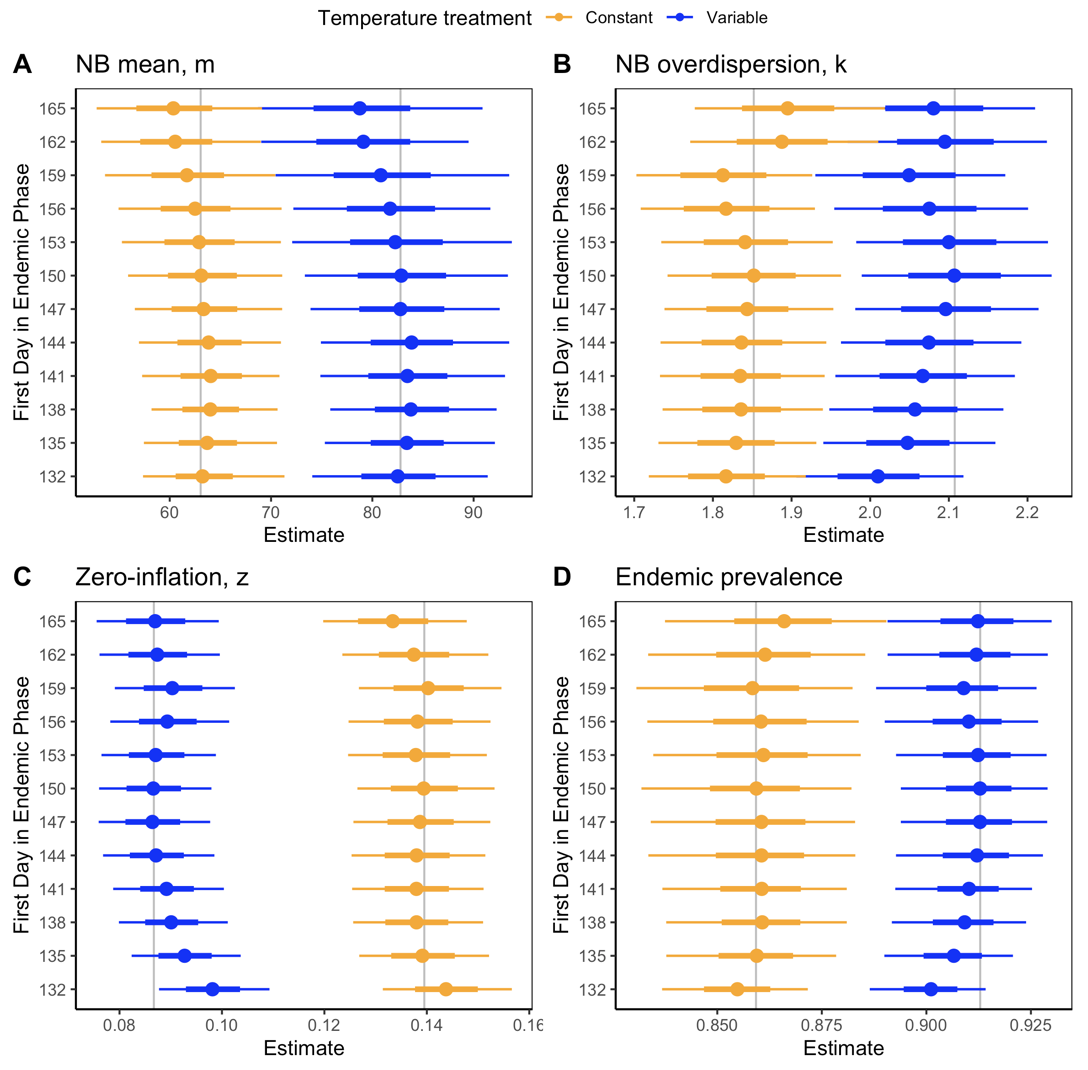

Supplement: S10 Fig — The ZINB and logistic regression models were re-fit to a series of candidate days for the first day of the endemic phase. We define the first day of the endemic phase as the first day that all replicate populations across constant and variable temperature treatments enter the stationary distribution representing equilibrium conditions of the disease. This was done to ensure that estimated posteriors were robust and that our results were not contingent on the chosen day for the first day of the endemic phase. The vertical gray line is the posterior estimate when the endemic phase starts at day 150, as we have assumed in the main text. The results of the sensitivity analysis confirm that our results are not contingent on when the endemic phase is assumed to begin. The data underlying this figure can be found in S1 Data. (TIF) [file pbio.3002260.s010.tif]
