# Supplementary material for: Short-term temperature fluctuations increase disease in a Daphnia-parasite infectious disease system
Source: PLoS Biol. 2023 Sep 8;21(9):e3002260. doi: 10.1371/journal.pbio.3002260 (PMC10491407; doi:10.1371/journal.pbio.3002260)
Supplement: S7 Fig — For each parameter being estimated in the ZINB model, plots in the left-hand column depict trace plots and plots in the right-hand column depict density plots for the fitted model. For all estimated parameters, the chains in the trace plot are well-mixed and suggest convergence. The mean of the negative binomial component of the model was estimated using treatment as a fixed effect (represented by βT in Eq 7.2, b[T] (Constant) and b[T] (Variable)), and replicate population as a random effect, (represented as αP in Eq 7.2. a[1]-a[6] are constant treatment replicates and a[7]-a[12] are variable treatment replicates). The overdispersion parameter, k ([k [1] constant, k [2] variable), and the probability of zero-inflation, z (theta [1] constant, theta [2] variable), were both estimated at the treatment level. The data underlying this figure can be found in S1 Data. (PDF) [file pbio.3002260.s007.pdf]

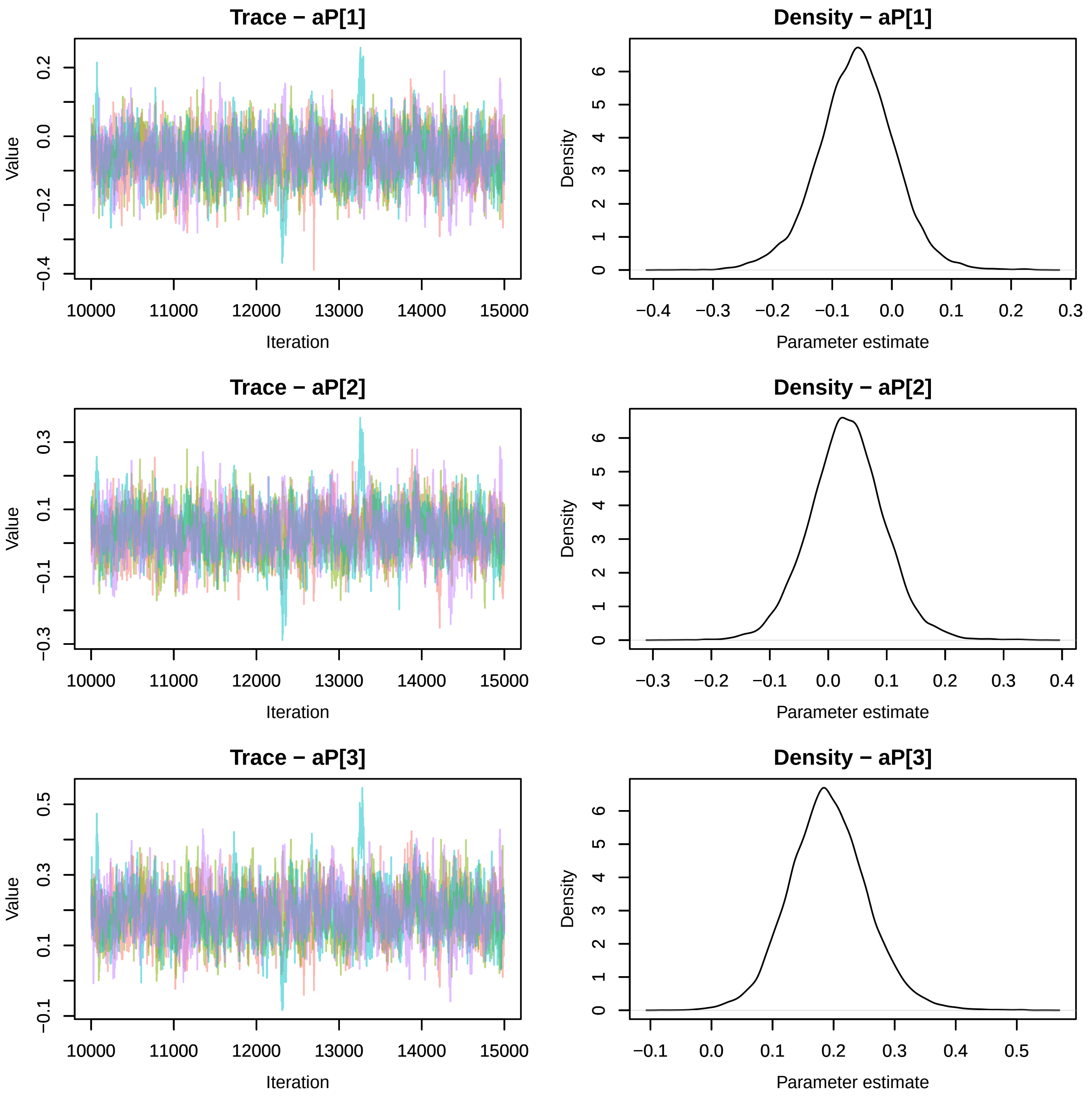

Trace − aP[1]
Value
Iteration
Density − aP[1]
Density
Parameter estimate
Trace − aP[2]
Value
Iteration
Density − aP[2]
Density
Parameter estimate
Trace − aP[3]
Value
Iteration
Density − aP[3]
Density
Parameter estimate

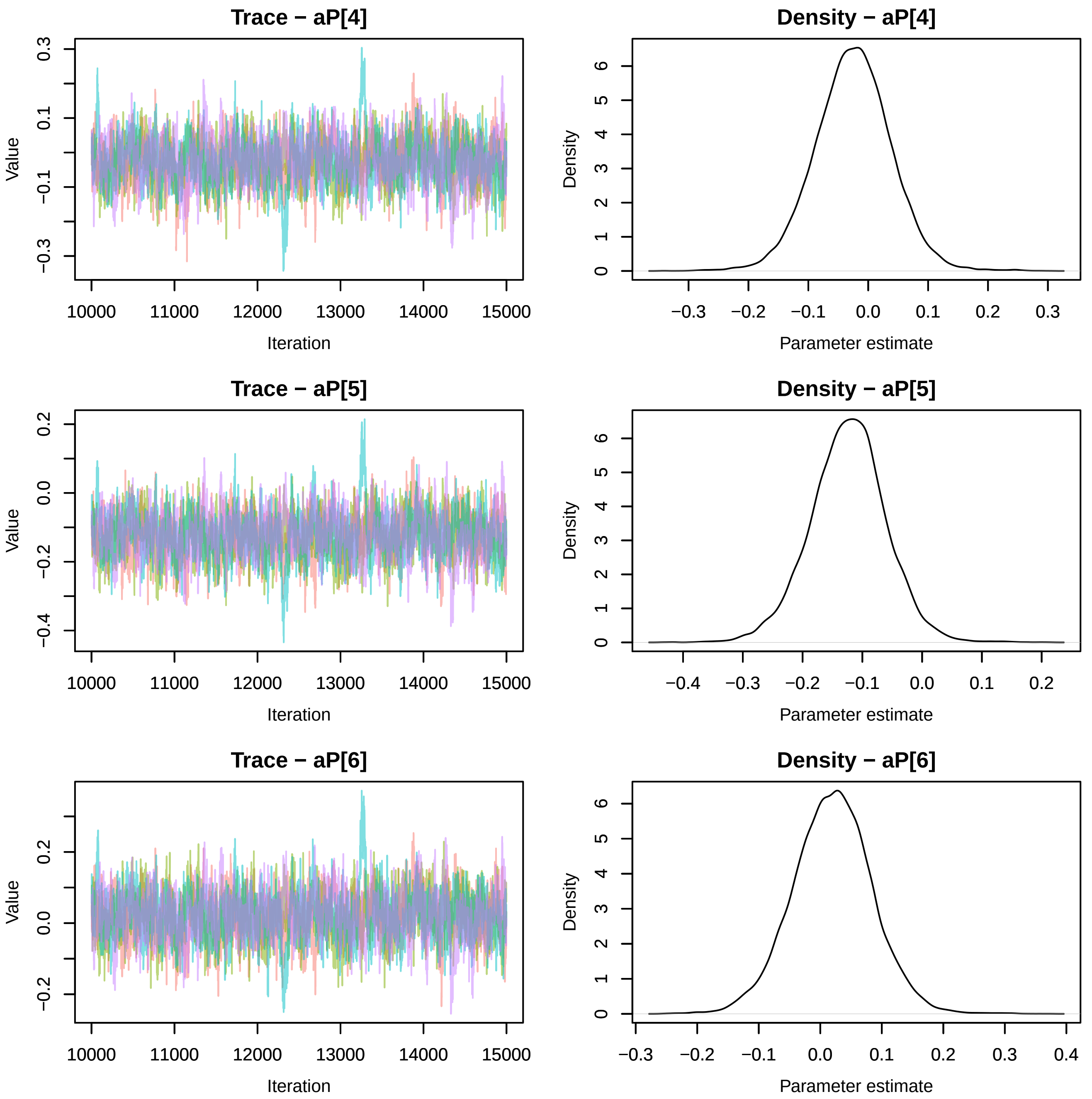
Trace − aP[4]
Value
Iteration
10000
11000
12000
13000
14000
15000
0.3
0.1
−0.1
−0.3
Density − aP[4]
Density
Parameter estimate
−0.3
−0.2
−0.1
0.0
0.1
0.2
0.3
0
1
2
3
4
5
6
Trace − aP[5]
Value
Iteration
0.2
0.0
−0.2
−0.4
Density − aP[5]
Density
Parameter estimate
−0.4
−0.3
−0.2
−0.1
0.0
0.1
0.2
Trace − aP[6]
Value
Iteration
0.2
0.0
−0.2
Density − aP[6]
Density
Parameter estimate
−0.3
−0.2
−0.1
0.0
0.1
0.2
0.3
0.4

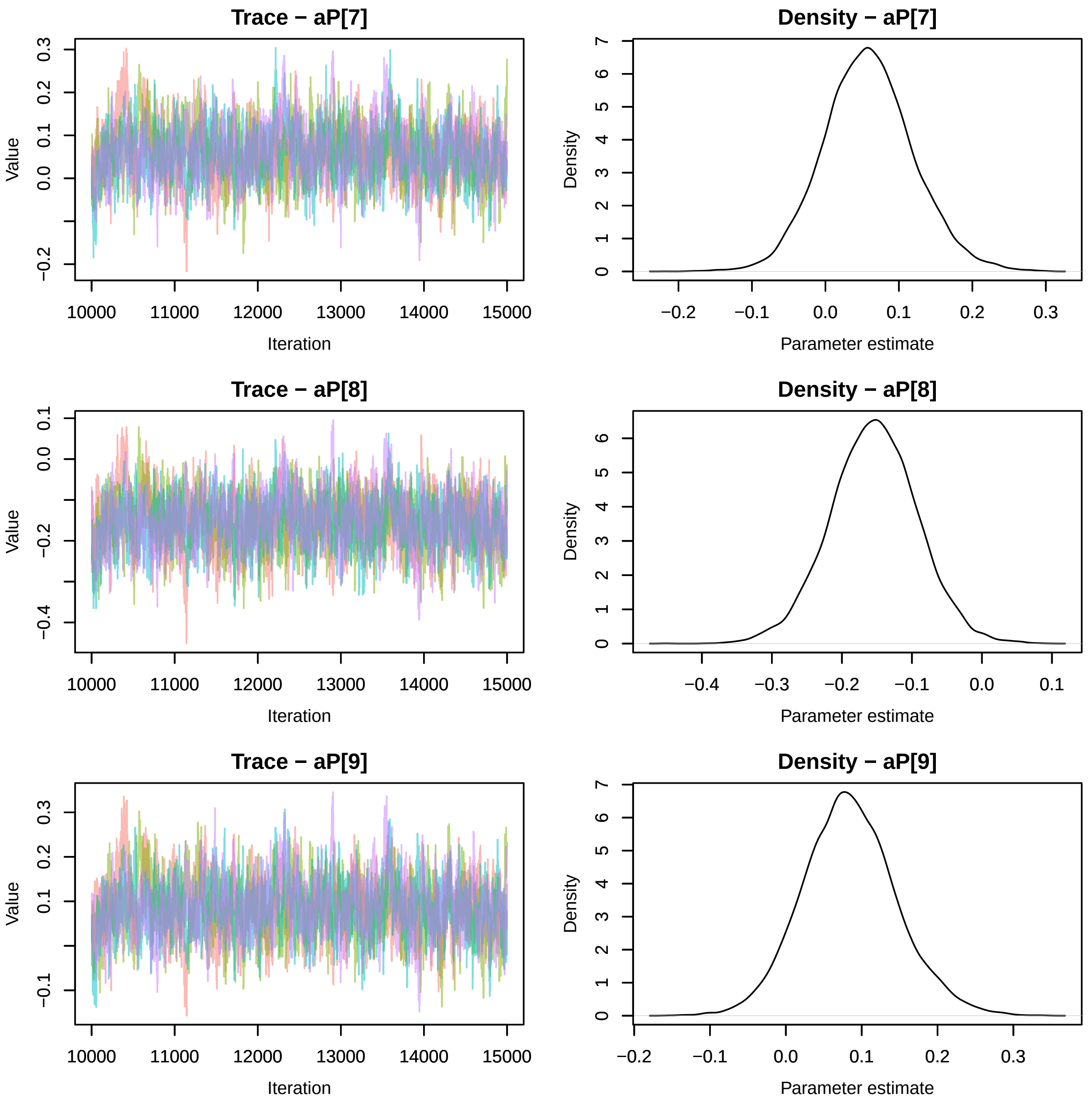
Trace − aP[7]
Value
Iteration
Density − aP[7]
Density
Parameter estimate
Trace − aP[8]
Value
Iteration
Density − aP[8]
Density
Parameter estimate
Trace − aP[9]
Value
Iteration
Density − aP[9]
Density
Parameter estimate

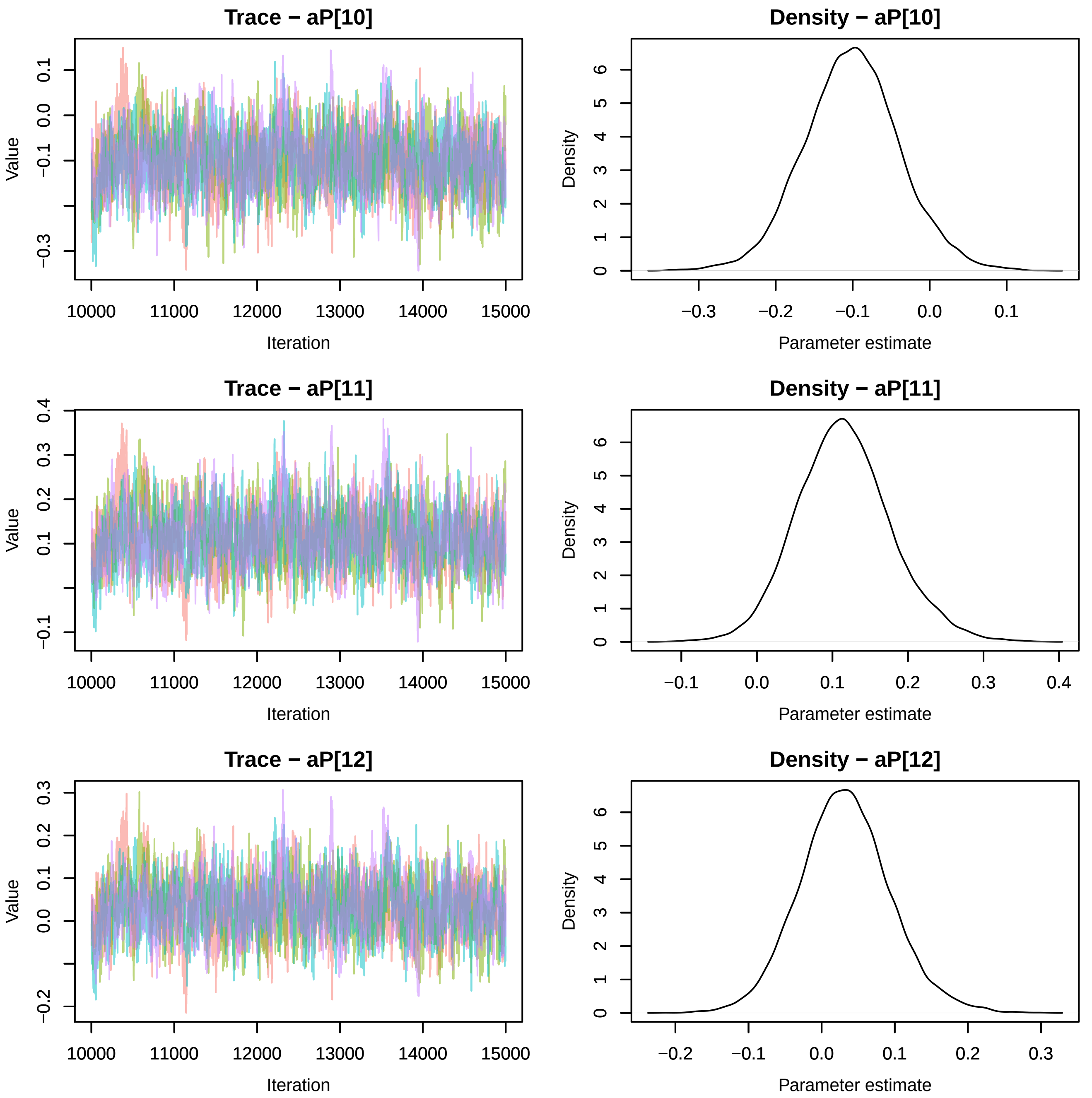

Trace − aP[10]
Value
Iteration
10000
11000
12000
13000
14000
15000
0.1
0.0
−0.1
−0.3
Density − aP[10]
Density
Parameter estimate
−0.3
−0.2
−0.1
0.0
0.1
0
1
2
3
4
5
6
Trace − aP[11]
Value
Iteration
0.4
0.3
0.2
0.1
−0.1
Density − aP[11]
Density
Parameter estimate
−0.1
0.0
0.1
0.2
0.3
0.4
Trace − aP[12]
Value
Iteration
0.3
0.2
0.1
0.0
−0.2
Density − aP[12]
Density
Parameter estimate
−0.2
−0.1
0.0
0.1
0.2
0.3

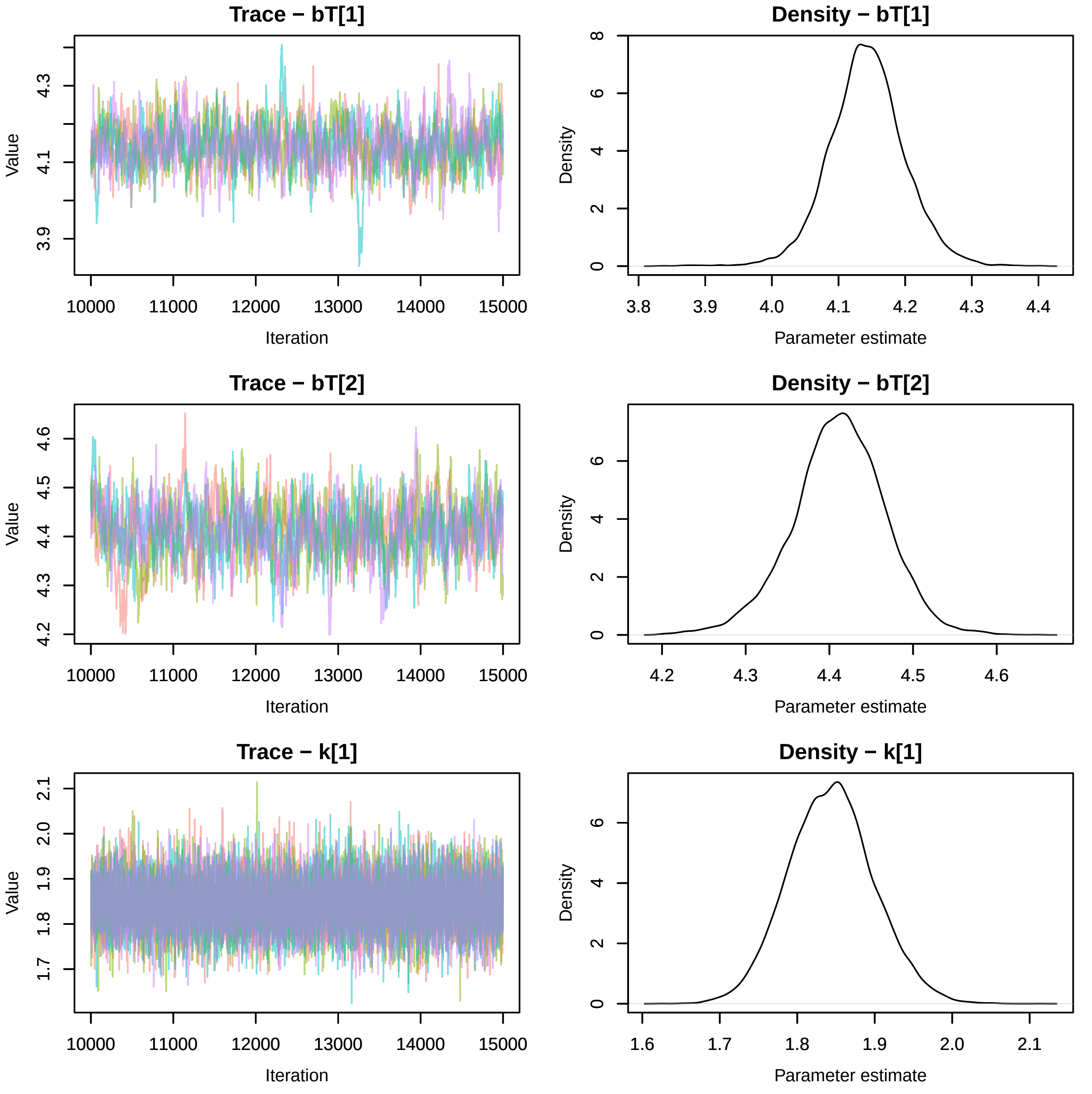
Trace − bT[1]
Value
3.9
4.1
4.3
10000
11000
12000
13000
14000
15000
Iteration
Density − bT[1]
Density
0
2
4
6
8
3.8
3.9
4.0
4.1
4.2
4.3
4.4
Parameter estimate
Trace − bT[2]
Value
4.2
4.3
4.4
4.5
4.6
10000
11000
12000
13000
14000
15000
Iteration
Density − bT[2]
Density
0
2
4
6
4.2
4.3
4.4
4.5
4.6
Parameter estimate
Trace − k[1]
Value
1.7
1.8
1.9
2.0
2.1
10000
11000
12000
13000
14000
15000
Iteration
Density − k[1]
Density
0
2
4
6
1.6
1.7
1.8
1.9
2.0
2.1
Parameter estimate

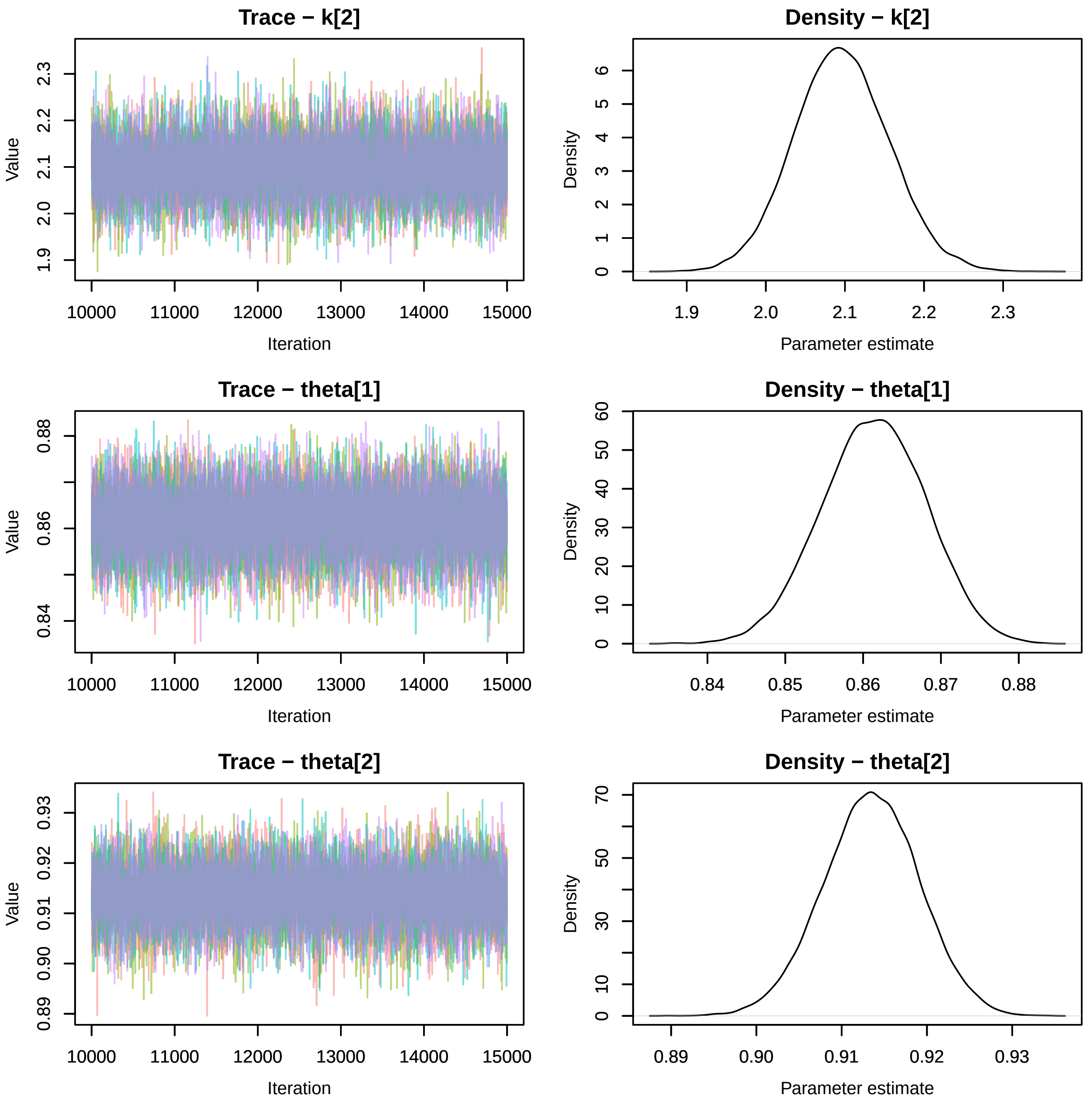
Trace − k[2]
Value
Iteration
10000
11000
12000
13000
14000
15000
1.9
2.0
2.1
2.2
2.3
Density − k[2]
Density
Parameter estimate
0
1
2
3
4
5
6
1.9
2.0
2.1
2.2
2.3
Trace − theta[1]
Value
Iteration
0.84
0.86
0.88
Density − theta[1]
Density
Parameter estimate
0
10
20
30
40
50
60
0.84
0.85
0.86
0.87
0.88
Trace − theta[2]
Value
Iteration
0.89
0.90
0.91
0.92
0.93
Density − theta[2]
Density
Parameter estimate
0
10
30
50
70
0.89
0.90
0.91
0.92
0.93
