# Supplementary material for: Short-term temperature fluctuations increase disease in a Daphnia-parasite infectious disease system
Source: PLoS Biol. 2023 Sep 8;21(9):e3002260. doi: 10.1371/journal.pbio.3002260 (PMC10491407; doi:10.1371/journal.pbio.3002260)
Supplement: S8 Fig — For each parameter estimated in the logistic regression model, plots in the left-hand column depict trace plots and plots in the right-hand column depict density plots for the fitted model. The probability of being infected, given by τ, was estimated using temperature treatment as a fixed effect (bT [1] constant, bT [2] variable) and replicate population as a random effect (aP[1]-aP[6] constant, a[7]-a[12] variable). All estimated parameters converge as indicated by well-mixed chains in the model trace plots. The data underlying this figure can be found in S1 Data. (PDF) [file pbio.3002260.s008.pdf]

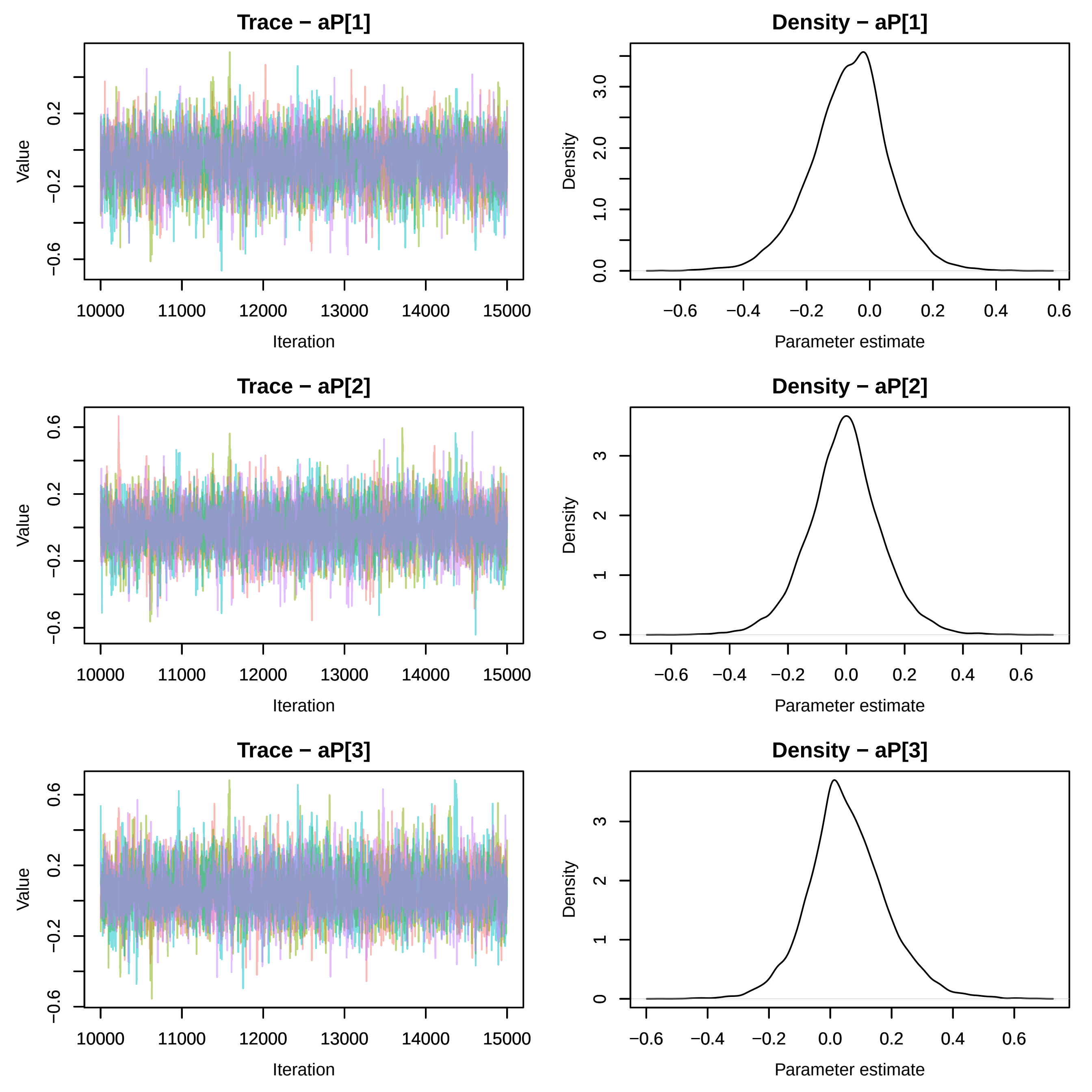

Trace − aP[1]
Density − aP[1]
Trace − aP[2]
Density − aP[2]
Trace − aP[3]
Density − aP[3]
Value
Iteration
Density
Parameter estimate
10000
11000
12000
13000
14000
15000
−0.6
−0.4
−0.2
0.0
0.2
0.4
0.6
1.0
2.0
3.0
0
1
2
3

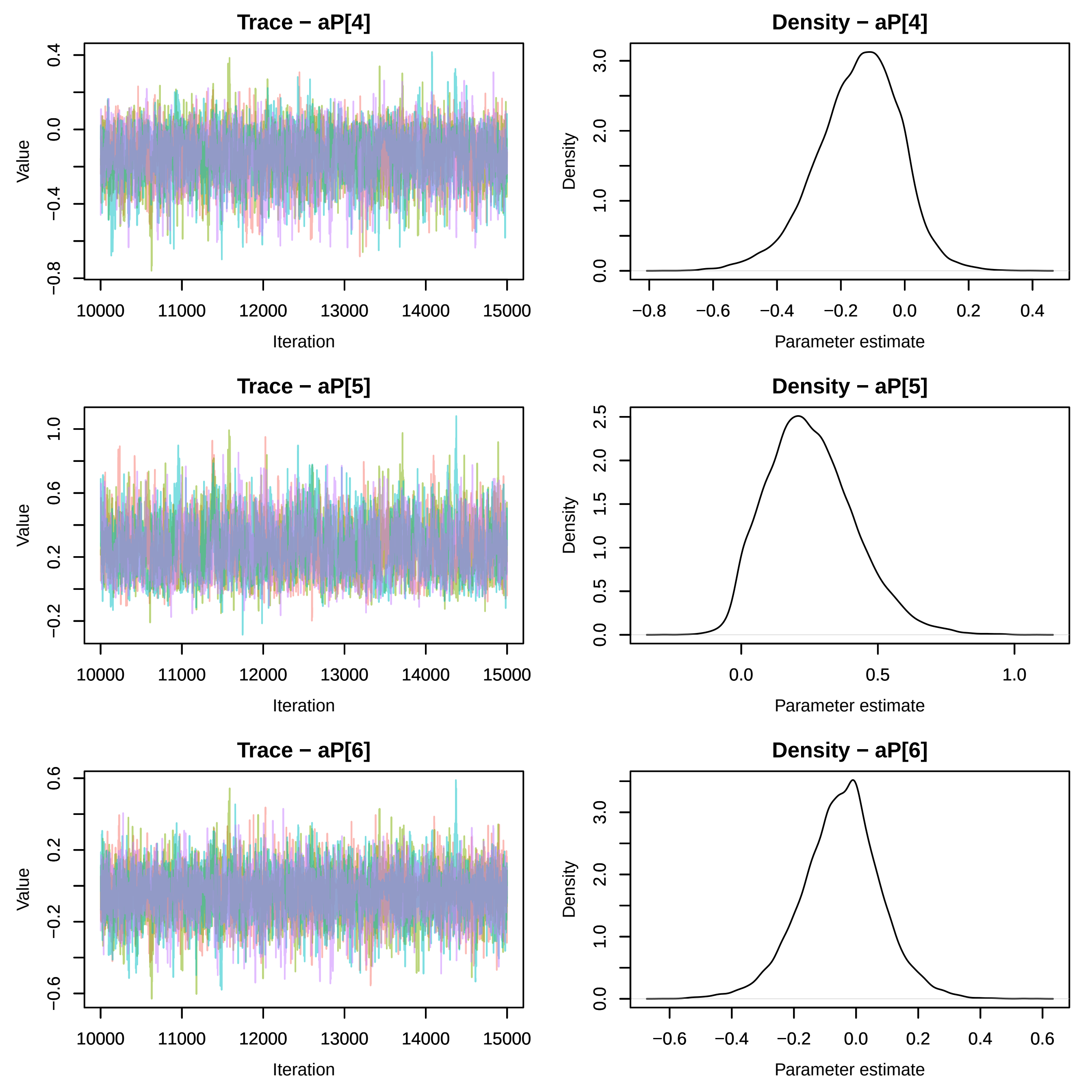
Trace − aP[4]
Value
Iteration
10000 11000 12000 13000 14000 15000
0.4 0.0 −0.4 −0.8
Density − aP[4]
Density
Parameter estimate
3.0 2.0 1.0 0.0
−0.8 −0.6 −0.4 −0.2 0.0 0.2 0.4
Trace − aP[5]
Value
Iteration
10000 11000 12000 13000 14000 15000
1.0 0.6 0.2 −0.2
Density − aP[5]
Density
Parameter estimate
2.5 2.0 1.5 1.0 0.5 0.0
0.0 0.5 1.0
Trace − aP[6]
Value
Iteration
10000 11000 12000 13000 14000 15000
0.6 0.2 −0.2 −0.6
Density − aP[6]
Density
Parameter estimate
3.0 2.0 1.0 0.0
−0.6 −0.4 −0.2 0.0 0.2 0.4 0.6

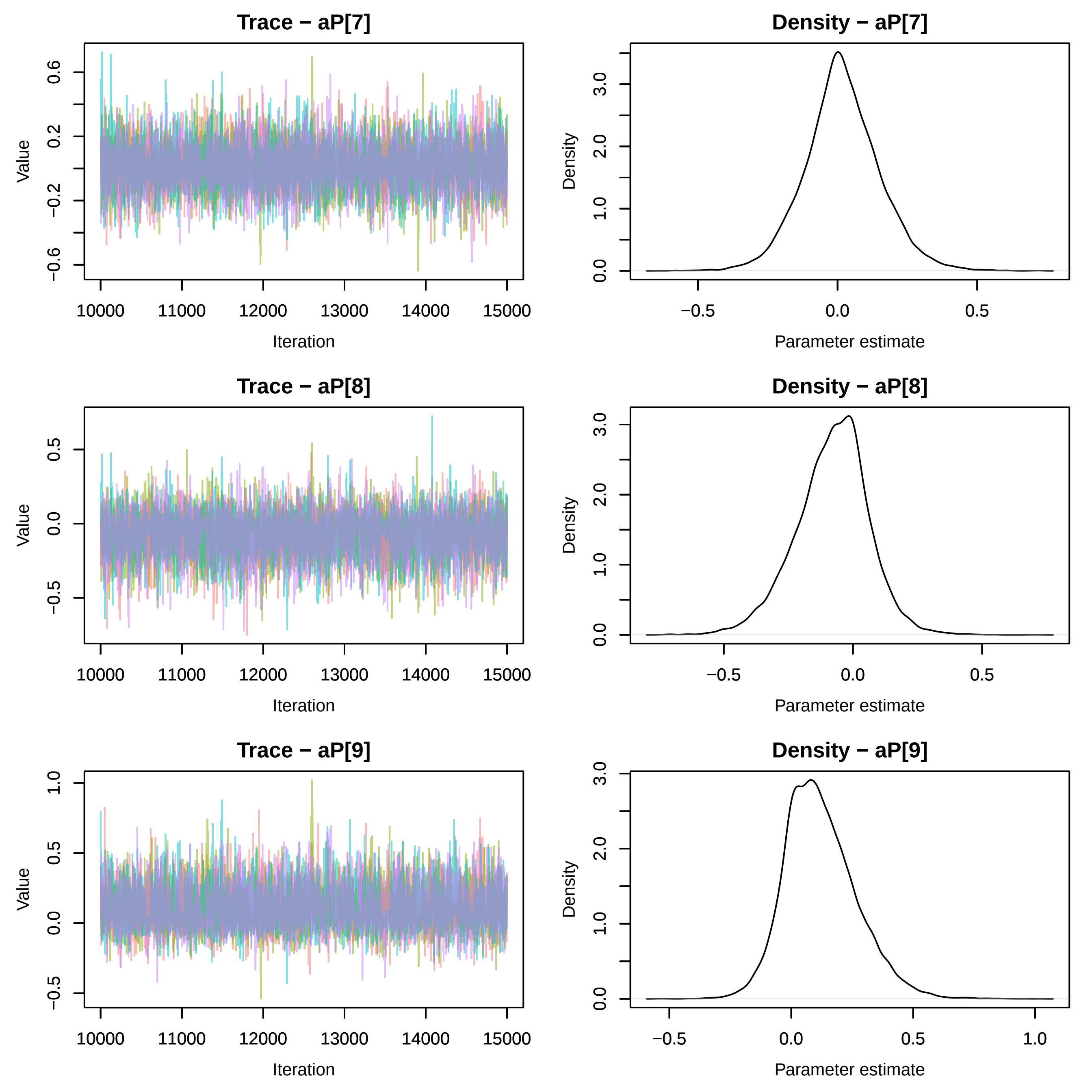

Trace − aP[7]
Value
Iteration
10000 11000 12000 13000 14000 15000
−0.6 −0.2 0.2 0.6
Density − aP[7]
Density
Parameter estimate
−0.5 0.0 0.5
0.0 1.0 2.0 3.0
Trace − aP[8]
Value
Iteration
10000 11000 12000 13000 14000 15000
−0.5 0.0 0.5
Density − aP[8]
Density
Parameter estimate
−0.5 0.0 0.5
0.0 1.0 2.0 3.0
Trace − aP[9]
Value
Iteration
10000 11000 12000 13000 14000 15000
−0.5 0.0 0.5 1.0
Density − aP[9]
Density
Parameter estimate
−0.5 0.0 0.5 1.0
0.0 1.0 2.0 3.0

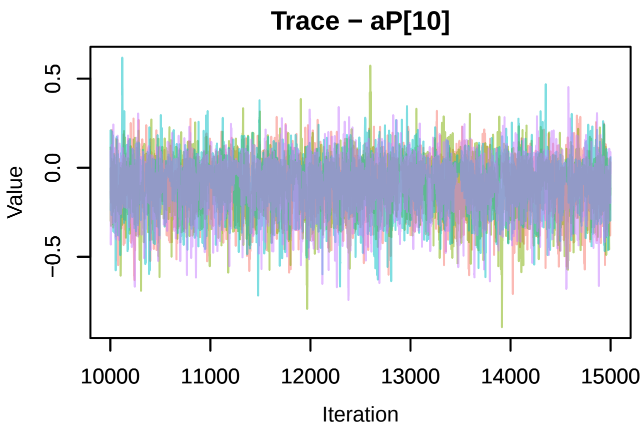
Trace − aP[10]
Value
0.5
0.0
−0.5
10000
11000
12000
13000
14000
15000
Iteration

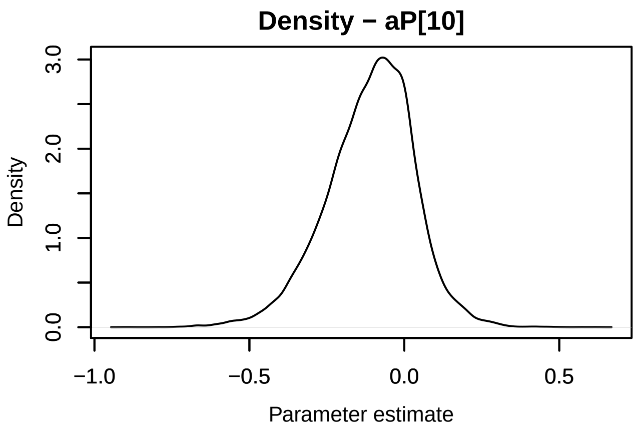
Density − aP[10]
Density
3.0
2.0
1.0
0.0
−1.0
−0.5
0.0
0.5
Parameter estimate

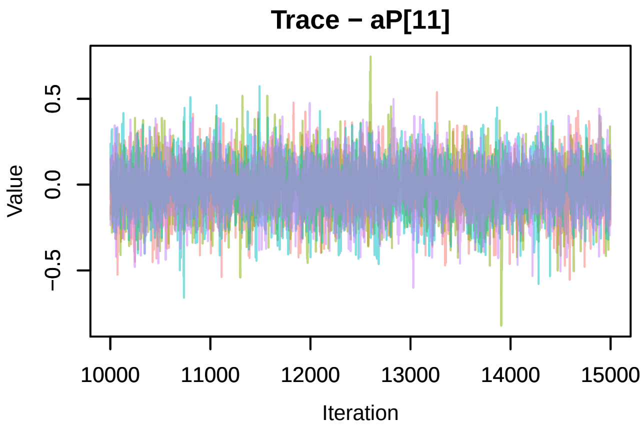
Trace − aP[11]
Value
0.5
0.0
−0.5
10000
11000
12000
13000
14000
15000
Iteration

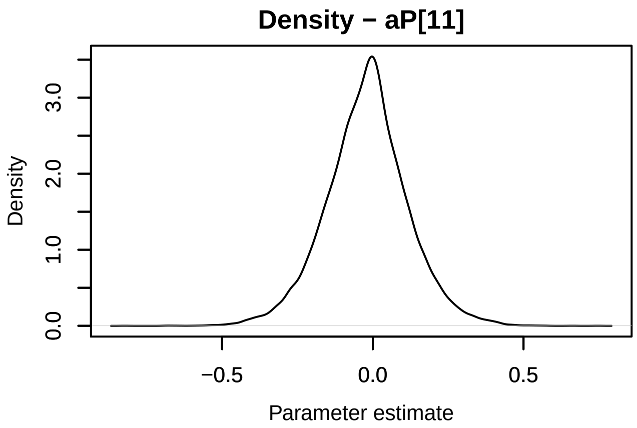
Density − aP[11]
Density
3.0
2.0
1.0
0.0
−0.5
0.0
0.5
Parameter estimate

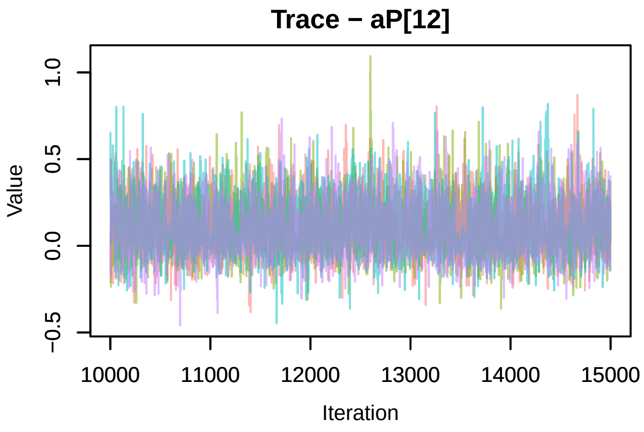
Trace − aP[12]
Value
1.0
0.5
0.0
−0.5
10000
11000
12000
13000
14000
15000
Iteration

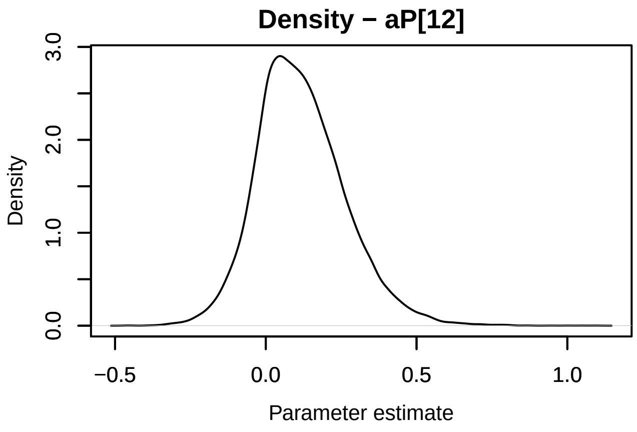
Density − aP[12]
Density
3.0
2.0
1.0
0.0
−0.5
0.0
0.5
1.0
Parameter estimate

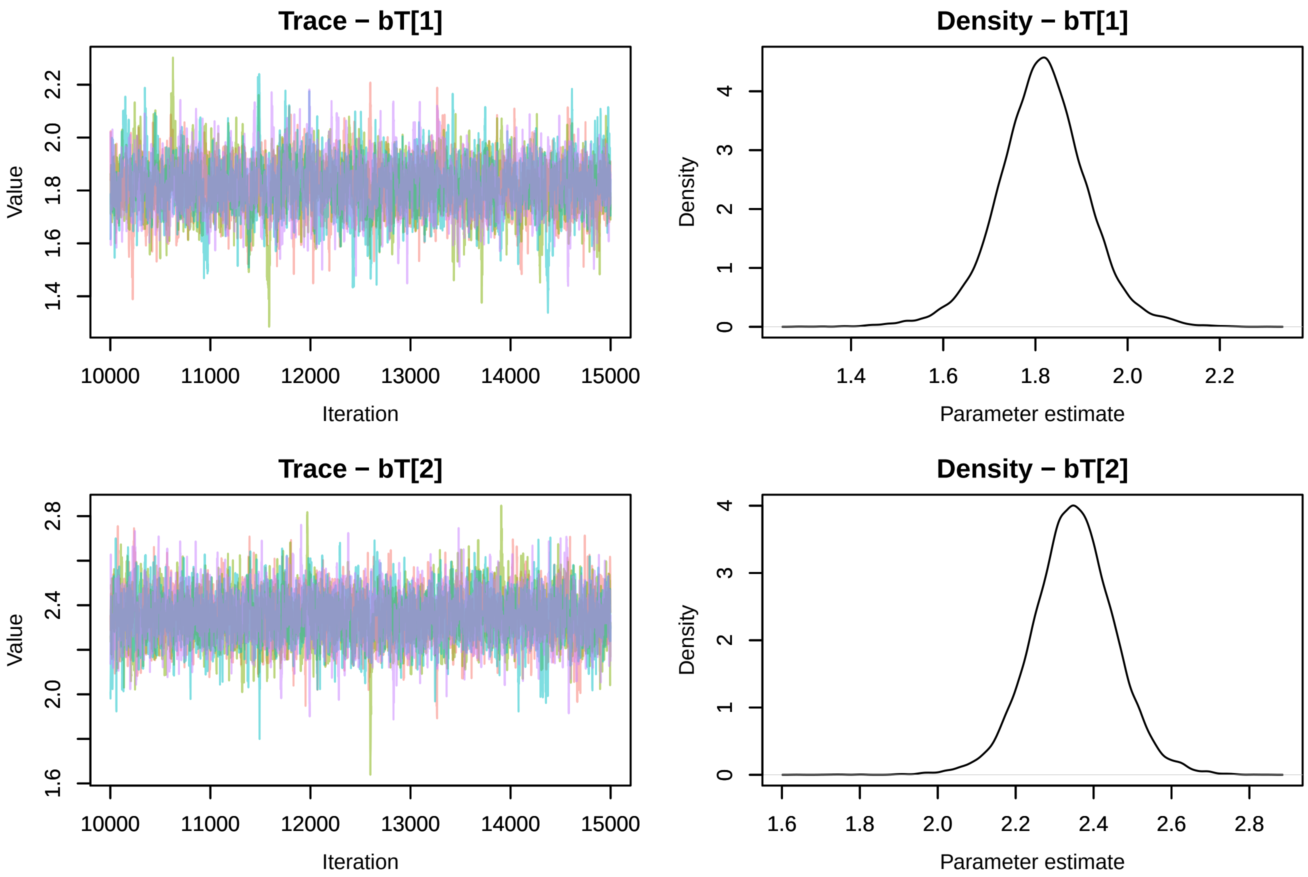
Trace − bT[1]
Density − bT[1]
Trace − bT[2]
Density − bT[2]
Value
Density
Iteration
Parameter estimate
10000
11000
12000
13000
14000
15000
