# Supplementary material for: Short-term temperature fluctuations increase disease in a Daphnia-parasite infectious disease system
Source: PLoS Biol. 2023 Sep 8;21(9):e3002260. doi: 10.1371/journal.pbio.3002260 (PMC10491407; doi:10.1371/journal.pbio.3002260)
Supplement: S1 Text — (DOCX) [file pbio.3002260.s018.docx]

**Full title:** Short-term temperature fluctuations increase disease in a *Daphnia*-parasite infectious disease system

**Short title:** Short-term temperature fluctuations increase disease

Leila Krichel^1*^, Devin Kirk^2^, Clara Pencer^1^, Madison Hönig^1,3^, Kiran Wadhawan^1,4^, Martin Krkošek^1^

1. Department of Ecology and Evolutionary Biology, University of Toronto, Toronto, Canada.
2. Department of Biology, Stanford University, Stanford, USA.
3. Department of Anthropology, Washington State University, Pullman, USA.
4. School of Biological Sciences, University of Edinburgh, Edinburgh, UK.

* Corresponding author

Email: [leila.krichel@mail.utoronto.ca](mailto:leila.krichel@mail.utoronto.ca) (LK)

**Supporting information**

**Temperature model**

Replicate populations in the variable temperature treatment each received a unique timeseries of daily temperature changes generated by a first-order temporal autoregressive model. Eq S1 describes temperature change as an autocorrelated process, where *T_t_* depends on the temperature at the previous time step, *T_t-1,_* and a normally distributed error term, *ε_t_ ~ Ν(0, sd)*. Centered around a mean of 0, this can be written as,

$T_{t}=\rho T_{t-1}+\epsilon_{t}$ Eq S1

ρ*­ =* 0.8 is the autocorrelation coefficient, which describes the degree of dependency between temperatures from one time step to the next. When ρ approaches 1, temperatures are more correlated through time, producing transient periods where the environment can enhance or preclude disease transmission. For the model (Eq 1.1-1.4), we used Eq S1 to generate 100 unique temperature time-series for each mean temperature (8°C-28°C) and level of the standard deviation of the error term, *ε_t_* (*ε_t_ ~ Ν(0, sd = 1)* or *ε_t_ ~ Ν(0, sd = 2)*). These error term levels correspond to low variability and high variability sub-treatments. Each temperature time-series comprised of a new temperature for each day the temperature changed (600 days). The standard deviation over the entirety of the time-series we denote as *sd**. On average, the low temperature variability sub-treatment *sd** = 1.7, and for the high temperature variability sub-treatment *sd** = 3.3.

For the experiment, temperature fluctuations occurred around the thermal optimum, T_opt_ = 20°C according to the high variability sub-treatment (i.e., where the error term of Eq S1 is *ε_t_ ~ N(0, sd = 2)*)*.* Centered around a mean temperature of 20°C, Eq S1 can generate temperatures that are very high which can elevate host mortality and make it difficult to maintain populations in the long-term. Since we were interested in the long-term dynamics of the epidemic, we limited experimental temperature fluctuations defined by Eq S1 to occur within a +/-6 range around 20°C (i.e., experimental populations experienced temperatures within a range of temperatures spanning 14°C to 26°C). We used this rule to simulate 6 unique temperature time series for each of the 6 replicate populations in the experimental variable temperature treatment (S1 Fig). The overall mean and standard deviation for each experimental temperature time series over the course of 228 days of the experiment is shown in S1 Table. The overall mean and standard deviation for each temperature time-series during the endemic phase (days 150-228) are also shown in S1 Table.

We also applied this rule generate 100 unique temperature variability sequences for theoretical predictions at the thermal optimum, as we wanted to simulate transmission dynamics under the experimental thermal conditions (Fig 4). This restriction on temperature fluctuations did not alter our qualitative predictions in terms of how the model is predicted to affect endemic prevalence at the thermal optimum (Figs 3 and 4).

**Temperature-dependency of the model**

The temperature-dependence of parameters for this *Daphnia*-parasite system (Table 1) can be described by TPCs obtained from Sharpe-Schoolfield MTE sub-models (Eq S2.1-S2.6 and Fig 2) (1,2). The MTE sub-models we used are composed of a Boltzmann-Arrhenius component that describes the exponential rising phase of TPCs and a Sharpe-Schoolfield component that describes the inactivation of rates at the ends of the temperature range. A detailed explanation of the structure of these models can be found in Molnár et al. (3). In general, *y_0_* is the rate of a given parameter at the reference temperature, *T_0_*. E_y_ is the activation energy of rate-limiting enzymes, E_Ly_ and E_Hy_ are inactivation energies at low and high temperatures, respectively, and *k* is Boltzmann’s constant (S2 Table). Temperatures are modelled in degrees Kelvin. We used five MTE sub-models, previously parameterized for this *Daphnia*-parasite system under constant environmental conditions, to capture the temperature-dependence of parameters in our model (Eqs 1.1-1.4). For each day the temperature changed according to Eq S1, the numerical value of a given temperature-dependent parameter could be obtained from its corresponding MTE sub-model (Eq S2.1-S2.6). An in-depth discussion of the parameterization of these sub-models is found in Kirk et al. (1,2), though we will provide a broad overview of how they fit into our modelling framework.

The contact rate between hosts and environmental spores at each temperature, *χ(T)*, is standardized for the volume of a 10L container and adult female *Daphnia* that are 2700um in length (Eq S2.1 and Fig 2A).

$\chi\left( T \right)=\chi_{0}\mathrm{mas}s^{\phi}e^{-\frac{E_{\chi}}{k}\left( \frac{1}{T}-\frac{1}{T_{0_{\chi}}} \right)} \cdot\left( 1 + e^{\frac{E_{H_{\chi}}}{k}\left( -\frac{1}{T}+\frac{1}{T_{H_{\chi}}} \right)} \right)^{-1}$ Eq S2.1

φ = 0.257 is the allometric scaling coefficient and captures how the contact rate at the reference temperature, *χ_0_*, scales with host body mass and the Boltzmann factor for the contact rate, $-\frac{E_{\chi}}{k}$, which describes the exponential rising phase of the function. In modelling the contact rate, we expected the contact rate to peak at some intermediate temperature, and therefore included an upper temperature threshold, T_Hχ_, where the contact rate is expected to drop-off. We did not, however, include a lower temperature threshold, as the filtration rate was not expected to sharply drop-off at low temperatures. Eq S2.1 can be modified to include the host acclimation response to changing temperatures as suggested by Rohr et al. (4). We did this by assuming the upper temperature threshold, T_Hχ_, linearly varies with the host acclimation temperature at time *t* (Eqs 3.1 and 4.1), such that $T_{H_{\chi}}\left( T_{acc}^{H}\left( t \right) \right)$.

We modelled the infection rate, $\eta\left( T \right),$ with upper and lower temperature thresholds, as infections are unexpected at the extreme ends of the temperature range (Eq S2.2).

$\eta\left( T \right)=\eta_{0}mass^{\psi}e^{-\frac{E_{\eta}}{k}\left( \frac{1}{T}-\frac{1}{T_{0_{\eta}}} \right)}\cdot\left( 1+e^{\frac{E_{L_{\eta}}}{k}\left( \frac{1}{T}-\frac{1}{T_{L_{\eta}}} \right)}+e^{\frac{E_{H_{\eta}}}{k}\left( -\frac{1}{T}+\frac{1}{T_{H_{\eta}}} \right)} \right)^{-1}$ Eq S2.2

ψ = -1.182 is the allometric scaling coefficient for the infection rate, which scales with host body mass and the Boltzmann factor of the infection rate, $-\frac{E_{\eta}}{k}$. Inactivation of the infection rate at each end of the temperature range is described by the term in brackets containing the inactivation energy at the lower temperature threshold, $T_{L_{\eta}}$, and the upper temperature threshold, $T_{H_{\eta}}$. Our model requires $\eta\left( T \right)$ to be converted from the infection rate to the probability of infection. This is done by accounting for how long a parasite remains in contact with a host (gut residence time of a parasite), the standardized filtration rate of *Daphnia*, and the size of a *Daphnia* individual (2). Acclimation in the infection rate (Eq S2.2) was accounted for in the lower and upper temperature thresholds. Because the infection rate is a result of host and parasite traits combining, acclimation responses were defined by allowing temperature thresholds to be either a function of the host acclimation temperature (Eq 3.1) or the parasite acclimation temperature (Eq 3.2) (S3 Fig, S3 Table).

Within-host infection intensity, *ω(T)*, is defined as equilibrium infection intensity, i.e., the equilibrium number of spore clusters in the gut epithelium of an infected *Daphnia* host (Eq S2.3 and Fig 2D). We assumed that once a host became infected, they were infected with the equilibrium number of spore clusters. According to Eq S2.3, this is equal to 103.1 at the T_opt_ = 20°C.

$\omega\left( T \right)=\omega_{0}e^{-\frac{E_{\omega}}{k}\left( \frac{1}{T}-\frac{1}{T_{0_{\omega}}} \right)}\cdot\left( 1+e^{\frac{E_{L_{\omega}}}{k}\left( \frac{1}{T}-\frac{1}{T_{L_{\omega}}} \right)}+e^{\frac{E_{H_{\omega}}}{k}\left( -\frac{1}{T}+\frac{1}{T_{H_{\omega}}} \right)} \right)^{-1}$ Eq S2.3

Eq S2.3 contains the Boltzmann factors describing the activation energy for within-host infection intensity and includes a lower, $T_{L_{\omega}}$, and upper, $T_{U_{\omega}}$, temperature threshold. We then used Eq S2.3 to derive TPCs for the infected host shedding rate, *λ(T),* and parasite-induced mortality, *α(T)*. Infection intensity at host death, which is equal to the equilibrium number of spore clusters, *ω(T),* was converted from the number of spore clusters to the number of individual spores. Here, we assumed that each spore cluster contained 64 spores, represented by *n* in the model (Eqs 1.1-1.4). Parasite-induced mortality was calculated as the product of spore cluster intensity at a given temperature and 5.12x10^-6^, the added per-parasite mortality on hosts (5). Like the infection rate, the within-host parasite equilibrium abundance emerges from host and parasite processes. Therefore, to account for acclimation in Eq S2.3, the upper and lower temperature thresholds could acclimate as a host or parasite trait (S3 Fig, S3 Table).

Finally, to calculate host natural mortality, *μ(Τ)*, we used a two-parameter Weibull distribution that allows for age-dependent changes in mortality (1). For *Daphnia* unexposed to the parasite, *U*, the Weibull distribution describes the survivorship of uninfected hosts over time. $\nu(T)$ is the scale parameter of the Weibull distribution and describes the host mortality rate (Eq S2.5). $\beta(T)$ is the shape parameter of the Weibull distribution and characterizes how the mortality rate changes over time (Eq S2.4). Both are temperature-dependent and have been previously described by MTE (1).

$\beta\left( T \right)=\beta_{0}e^{\frac{-E_{\beta}}{k}\left( \frac{1}{T}-\frac{1}{T_{0_{\beta}}} \right)}\cdot\left( 1+e^{\frac{E_{H_{\beta}}}{k}\left( -\frac{1}{T}+\frac{1}{T_{H_{\beta}}} \right)} \right)^{-1}$ Eq S2.4

$\upsilon\left( T \right)=\upsilon_{0}e^{-\frac{E_{\upsilon}}{k}\left( \frac{1}{T}-\frac{1}{T_{0_{\upsilon}}} \right)}\cdot\left( 1+e^{\frac{E_{L_{\upsilon}}}{k}\left( \frac{1}{T}-\frac{1}{T_{L_{\upsilon}}} \right)}+e^{\frac{E_{H_{\upsilon}}}{k}\left( -\frac{1}{T}+\frac{1}{T_{H_{\upsilon}}} \right)} \right)^{-1}$ Eq S2.5

Eq S2.4-S2.5 can then be used to describe age-dependent changes in the natural mortality rate of unexposed hosts, *U,* over time, *t*. At each temperature, this can be defined as

$\frac{dU}{dt}= -\beta\left( T \right)\cdot\upsilon\left( T \right)^{\beta\left( T \right)}\cdot t^{\beta\left( T \right)-1}\cdot U$ Eq S2.6

and describes survivorship of unexposed hosts over time. Eq S2.6 therefore describes how the mortality rate changes over time. Since the model requires $\upsilon$ to be expressed as a constant natural mortality rate across all hosts, we used Eq S2.6 to calculate the host’s expected lifespan, i.e., the timepoint at which the survival probability was 0.5. This allowed us to calculate a constant mortality rate, *μ,* as 1/expected lifespan at different temperatures. To model *μ(T)* in variable environments, we simulated Eq S2.6 and calculated the mortality rate for each day the temperature changed across replicate simulations. Eq S2.6 was implemented in R using the *deSolve* package (6,7).

All other parameters were modelled as temperature independent and reflect experimental conditions. We assumed the carrying capacity, *K*, to be 150, which is roughly consistent with our experimental observations in constant and variable treatments and observations of our lab stocks at 20°C. Note that the value of *K* used in the model did not alter our model predictions for endemic prevalence. To ensure that populations remained around the carrying capacity, we set the adult recruitment rate, *b,* to 1. Hosts were harvested from each replicate population at rate *h* = 0.02666 d^-1^. This is derived from the experimental protocol where 12 *Daphnia* individuals were sampled from each population of 150 individuals every third day (S4 Fig). For the experiment, the 12 hosts that were sampled out from each replicate population were replaced on the same day with 9 uninfected hosts from susceptible lab stocks and 3 hosts from infected lab stocks. The replacement of individuals sampled out at rate *h* is represented in the model by input terms for susceptible hosts, *ζ_S_*, and infected hosts, *ζ_I_*. To derive the rate of input for susceptible and infected hosts, we first assumed that infection prevalence in infected lab stocks is equal to the estimate of endemic infection prevalence, ~0.86, at 20°C in the constant experimental treatment. Then, the rate at which infected and susceptible hosts are introduced per day is *ζ_I_* = 0.86 (3 infected hosts / 3 days * 0.86) and *ζ_S_* = 3.14 (9 susceptible hosts / 3 days + (1 – 0.86)), respectively.

We assumed that adult *Daphnia* were 2700*um* in length, which reflects previous observations from our lab stocks. We assumed that spore clusters infecting the *Daphnia* host’s gut epithelium took 3 days to burst and we assumed that each spore cluster contained 64 individual spores. Once a cluster burst, we assumed that half the spores were shed into the environment by infected *Daphnia* at rate, *λ*, and that the other half reinfected the same host. Dead infected hosts degraded at a rate *θ* = 0.01 d^-1^, which was parameterized by measuring the time at which the *Daphnia* gut was completely degraded (5). The environmental spore mortality rate was set to *γ* = 0.025 d^-1^ (0.75 of 10L removed every 3 days) and reflects the experimental removal of medium. Because environmental spore stages are capable of enduring unfavourable conditions, the removal of experimental medium was assumed to overwhelm natural spore mortality (8).

Theoretical predictions of endemic prevalence were initially overestimated relative to our experimental observations in constant and variable temperature treatments. As a result, we introduced a scaling coefficient, *c* = 0.00935, to the transmission component of the simulation model (Eqs 1.1-1.4). We chose *c* by lining up theoretical predictions of endemic prevalence at T_opt_ = 20°C from the deterministic model for constant environmental conditions with our observations of endemic prevalence in the experimental constant treatment (Fig 4, dashed vertical line).

**Analysis of the model with nonlinear averaging**

For variable environmental conditions, analysis of the model using nonlinear averaging was done by averaging the deterministic prediction of the endemic prevalence thermal response curve (Eqs 1.1-1.4, Fig 3, yellow curve) over a probability distribution of temperatures (Eq 5). To numerically implement nonlinear averaging, we discretized the probability distribution of environmental temperatures (Eq S3.1).

$Q\left( T=\tau\right)=\frac{P\left( T=\tau\right)}{\sum_{j} P\left( T=\tau_{j} \right)}$ Eq S3.1

*Q* is the discretized probability distribution of environmental temperatures. Based on our temperature model (Eq S1), *Q* is a normal distribution, where the mean is equal to the mean temperature of the environment. The standard deviation of *Q* was found by finding the mean standard deviation across 100 replicate simulations of Eq S1 at a given mean temperature. Eq 5 can be re-written as a Riemann’s sum, where endemic prevalence across constant mean temperatures, $w\left( \overline{T_{j}} \right)$, is weighed by the probability distribution of temperatures, *Q*.

$\overline{w\left( T \right)}=\sum_{j} w\left( \overline{T_{j}} \right)\cdot Q\left( T=\tau_{j} \right)$ Eq S3.2

**Analysis of the model with acclimation**

To model the effects of acclimation on disease transmission dynamics, we used the framework proposed by Rohr et al. (4). Because parasites are smaller than their hosts, parasites can more rapidly acclimate to temperature changes, therefore providing parasites with an advantage over their hosts when there are short-term thermal fluctuations. We incorporated acclimation in the model (Eq 1.1-1.4) by allowing the upper and lower temperature thresholds of trait TPCs described by MTE sub-models (Eq S2.1-S2.5) to be functions of the acclimation temperature of the host or the parasite (Eq 3.1-3.2). Accounting for acclimation in the MTE sub-models shifts the shape of trait TPCs to affect disease transmission dynamics defined in the model (Eq 1.1-1.4). Our acclimation model makes several assumptions, summarized in our modelling design in S3 Table.

First, we assumed that parasites should acclimate faster than their hosts with a ratio proportional to the parasite-to-host mass ratio (Eq 2). Assuming Eq 2 holds for parasites, we assumed that parasites were 1000 times smaller in mass than their hosts to derive the corresponding parasite-to-host time to acclimation ratio, equal to 1:6. This is interpreted as the parasite taking a single day, $\psi_{P}$, to acclimate to a new temperature relative to the host’s 6 days, $\psi_{H}$. We accounted for uncertainty in the parasite-to-host mass ratio but doing so did not significantly shift the parasite-to-host time to acclimation ratio. Instead, we directly varied the number of days it takes for the host to acclimate to a new temperature, $\psi_{H},$and analyzed the model using three host acclimation time treatments: 6, 12, and 18 days. Respectively, these three treatments correspond to fast, medium, and slow time to acclimation of the host relative to the parasite, the latter of which is always assumed to acclimate within a single day, $\psi_{P}=1$ (S3 Table). For the parasite, $\psi_{P}=1$ can be interpreted as instantaneous acclimation, such that the parasite acclimation temperature perfectly tracks environmental temperature changes over time (Fig 1). Another assumption is that the acclimation time of the host, $\psi_{H}$, is the same for all host traits, and the acclimation time of the parasite, $\psi_{P},$ is the same for all parasite traits.

We also made several assumptions about the relationship between the thermal thresholds of temperature-dependent traits (Table 1 and Eq S2.1-2.5) and the acclimation temperature. First, like Rohr et al. (4), we assume that this relationship is linear (Eq 4.1-4.2). The strength of the linear relationship, represented by the slope of Eq 4.1-4.2, modifies the shape of TPCs—here, higher slopes more strongly influence the shape of TPCs such that the effects of beneficial acclimation responses are stronger (S2 Fig). Calculating the slope Eq 5.1-5.2 requires measuring the upper and lower thermal limits at different acclimation temperatures. Since we do not possess this data for our system, we arbitrarily chose two slopes, 0.1 and 0.4, corresponding to weak and strong beneficial acclimation effects. We also assume that these slopes are the same for all temperature-dependent parameters, regardless of whether the trait belongs to the host or the parasite.

Finally, we also assumed whether specific temperature-dependent traits acclimated according to the host or the parasite. The contact rate and the host mortality rate are clearly host traits, and therefore acclimate according to the number of days it takes for the host to acclimate to a new environmental temperature ($\psi_{H}$, Eq 2). However, the infection rate and the within-host parasite equilibrium abundance are emergent traits that come out of the way host and parasite processes combine. For example, the infection rate may be the product of the parasite’s propensity to infect a host and the host’s ability to resist infection. In our acclimation model, we therefore allowed the infection rate and within-host equilibrium infection intensity to acclimate according to the number it takes for the host, $\psi_{H}$, or the parasite, $\psi_{P}$, to acclimate (S3 Fig, S3 Table).

**Experimental design**

We tested our theoretical predictions of endemic prevalence at the thermal optimum, T_opt_ = 20°C, using experimental epidemics of the microsporidian parasite, *Ordospora*, in *Daphnia* populations. We followed the spread of the disease in 12 replicate populations of *Daphnia* held at a mean temperature of 20°C for 228 days. Replicate populations could be in either the constant temperature treatment, where temperatures remained at 20°C throughout the experiment, or in the variable temperature treatment, where temperatures fluctuated around an average of 20°C and within a range of temperatures spanning 14°C and 26°C (Eq S1 and S1 Fig). Two weeks prior to the experiment, susceptible and infected asexual female *Daphnia* were acclimated in environmental chambers set at a constant 20°C. After the acclimation period, we randomly assigned 150 *Daphnia* individuals to a replicate population, and replicate populations were then randomly assigned to the constant or variable temperature treatment. Each replicate population was placed in a container holding 10L of *Daphnia* growth medium (AdaM; (9)).

The experiment took place in two environmental chambers set at a constant 20°C. Each environmental chamber held 6 replicate populations (12 populations total). Within a single environmental chamber, 3 replicate populations were in the constant temperature treatment and 3 replicate populations were in the variable temperature treatment. Constant temperature treatment populations were placed directly on shelves as the ambient temperature of the chamber was already set to 20°C. Variable temperature treatment populations were each placed in their own water bath set up in the environmental chamber. We used temperature controllers attached to water heaters and chillers to change the temperature of each water bath, which then changed the temperature of containers holding each variable temperature treatment replicate population. Each variable treatment population received a unique sequence of temperature changes, which occurred at the same time daily (Eq S1 and S1 Fig). Temperature fluctuations simulated by Eq S1 were restricted to occur within a +/-6 range around 20°C.

The experiment lasted for 228 days, and the same sampling protocol was followed for constant and variable temperature treatments (S4 Fig). To initiate the epidemic in each replicate population, we introduced 3 *Daphnia* from infected lab stocks. Infected individuals introduced to the experiment were acclimated to 20°C in environmental chambers 2 weeks prior to their input. Every third day, we sampled 12 *Daphnia* individuals from each experimental replicate population. Individuals sampled out during the experiment is a process accounted for in the model with the harvesting term, *h*. From each sub-sample collected during the experiment, we destructively determined the infection status of *Daphnia* individuals under a microscope. This included (1) checking whether individuals were infected and (2) counting the number of spore clusters infecting their gut epithelium. This allowed us to obtain timeseries of infection prevalence and infection burden over time (S5 Fig).

On the same day that we sampled replicate populations, we replaced the 12 sampled individuals with 12 individuals from lab stocks acclimated to 20°C. We did this minimize disturbance on populations from harvesting and to continuously introduce the disease at a low rate throughout the experiment. Of the 12 replacement individuals, 9 were randomly sampled from susceptible lab stocks and 3 were randomly sampled from infected lab stocks. We assumed that infected lab stocks were in their endemic phase and therefore had an infection prevalence equal to 0.86 (this is based on our endemic phase experimental results at 20°C in the constant temperature treatment, Fig 4). Therefore, for every third day of the experiment that we replaced sampled individuals with 12 new individuals from acclimated lab stocks, 3 * 0.86 = 2.58 were infected and 9 + (3 – 2.58) = 9.42 were susceptible. This was accounted for in the model (Eq 1.1-1.4) by converting these numbers into daily input (i.e., immigration) rates, where $\xi_{S}$ = 3 + (1 – 0.86) = 3.14 and $\xi_{I}$ = 0.86. On each observation day, each population was fed 250 million algal cells and 0.75L of experimental medium was removed and replaced with fresh medium to prevent the overgrowth of algae (*Monoraphidium minutum*).

**Experimental data of endemic phase**

S5 Fig shows replicate timeseries for infection prevalence and mean infection burden in constant and variable temperature treatments over the course of the experiment (228 days). Based on our theoretical predictions at T_opt_ = 20°C (Figs 3 and 4), we set out to determine whether temperature variability suppressed endemic prevalence. We define the endemic phase as the stationary distribution of the disease, characterizing the disease’s equilibrium conditions. We truncated the experimental data so that only observations from the endemic phase were included in our analysis (day 150 - day 228; S5 Fig, shaded grey area). Truncating the timeseries left us 27 data points, though we note that missing data on observation day 192 reduces this to 26 data points. To determine when all replicate populations were in the endemic phase, we conducted a sensitivity analysis, the details of which are discussed in the last section.

We estimated endemic prevalence by fitting a logistic regression model to the data representing binary infection status data (Eq 6.1-6.2). This binary data was obtained in the experiment by determining whether a sampled host was infected or uninfected. The other data type we obtained was count data representing the number of spore clusters infecting a host. Hosts can be uninfected with 0 spore clusters or infected with more than 0 spore clusters. The data for host infection burden show that there is considerable variation in the number of spore clusters infecting *Daphnia* hosts at the endemic phase and that the distribution of infection burden is overdispersed (S6 Fig). This indicates that the distribution of parasites across the host population can be described by overdispersed distributions like the negative binomial or zero-inflated negative binomial model.

Using the Markov chain Monte Carlo (MCMC) software JAGS, we fit negative binomial (NB) and zero-inflated negative binomial (ZINB) models to host infection burden data during the endemic phase (S9 Fig) (7,10–12). Initially, we fitted the negative binomial distribution, as this is what is typically used to describe parasites that are overdispersed. The negative binomial likelihood function can be written as,

$y_{\mathrm{nij}}\sim NB\left( m_{ij},k_{i} \right)$ Eq S4.1
$\log\left( m_{ij} \right)=\beta_{T}T_{i}+a_{P}P_{j}$ Eq S4.2

Where, *m* is the mean, which varies by treatment, *i*, and population, *j*, and *k* is the overdispersion parameter, which varies by treatment, *i*. Estimating these two parameters fully characterizes the distribution of parasites across their hosts as an NB process in constant and variable temperature treatments. While the NB model captured our observations of mean infection burden (S9B Fig), it overestimated observations of endemic prevalence in constant and variable temperature treatments (S9A Fig). Relative to the NB model, it was clear that the ZINB model (Eq 7.1-7.2) better described experimental observations of endemic prevalence across replicate populations (S10 Fig). While the NB model is characterized by a single aggregating process, namely, the overdispersion parameter, *k*, the ZINB model is characterized by two aggregating processes: the overdispersion parameter, *k*, and the probability of zero-inflation, *z*. This implies that parasites in this *Daphnia*-parasite system are more aggregated across their hosts relative to an NB model.

The fitted ZINB model captured the distribution of infection burden per host during the endemic phase (Fig 5D). For each line in Fig 5D, we obtained 5000 random realizations from the ZINB characterized by a set of posterior estimates for *z_i_*, *m_i_*, and *k_i_,* where *i* is the treatment-specific posterior (Fig 5A-C). The set of posterior estimates characterizing the ZINB distribution for a particular permutation was chosen by randomly selecting a chain, then randomly selecting a position on that chain to obtain *z_i_*, *m_i_*, and *k_i_*. We then used that set of posterior estimates to characterize the fitted ZINB distribution using the *rzinbinom* function in R (*emdbook* package) and to randomly sample 5000 observations from that particular permutation of the ZINB model fit.

Each random realization from the ZINB model fit can be interpreted as the number of spore clusters infecting a randomly sampled host during the endemic phase. Thus, it can be used to derive population-level properties like endemic prevalence and mean infection burden (Fig 5E-F). From the ZINB distribution characterized by the set of posterior estimates *mi*, *ki*, and *z_i_,* we randomly sampled 150 times and calculated mean infection burden by averaging over the sample and endemic prevalence by finding the proportion of non-zero samples (i.e., infected individuals). We repeated this process 5000 times to get predicted distributions for endemic infection prevalence and mean infection burden in constant and variable temperature treatments (Fig 5E-F).

To get estimates of endemic prevalence and mean infection burden at the replicate population level we conducted the same protocol as above only this time accounting for population effects. This time, the fitted ZINB distribution is characterized by a set of posterior estimates for *mij*, *ki*, and *z_i_.* Subscript *i* indicates variation by treatment and *j* indicates variation by replicate population. From the fitted ZINB models for each replicate population, we first sampled from the distribution 12 times—this mimics the experiment wherein we sampled 12 hosts from each replicate population. Averaging over the sample calculates mean infection burden and finding the proportion of non-zero samples calculates endemic infection prevalence. We repeated this process 26 times to match the number of endemic phase data points from the experiment. This allowed us to compare ZINB-generated and experimental observations of endemic prevalence and mean infection burden in each population (Figs 6 and S9). This same protocol can be followed to describe endemic prevalence and infection burden as an NB process, characterized by *mij* and *ki* (S9 Fig).

**Sensitivity analysis**

The analysis of our experimental data assumes that all replicate populations in constant and variable temperature treatments have entered the endemic phase by day 150 of the experiment (S5 Fig, shaded grey area). The beginning of the endemic phase was determined with a sensitivity analysis, which defined where to truncate the timeseries data (S10 Fig) and determined whether our main results were contingent on where we defined the beginning of the endemic phase. The sensitivity analysis was performed by re-fitting the logistic regression (Eq 6.1-6.2) and ZINB models (Eq 7.1-7.2) to a series of candidate start days +/- 5 observation days from day 150 and was used to determine whether the posteriors for estimated parameters were robust (S10 Fig). Overall, the directionality of the posterior estimates across parameters in constant and variable treatments remained the same regardless of when the endemic phase is assumed to begin. The posteriors for the overdispersion parameter, *k*, exhibited wavering, though this may be expected since fewer data points were used for estimation later in the time-series. The results of this sensitivity analysis indicate that our conclusions are robust and that the observed patterns (Figs 5 and 6) are representative of processes occurring during the endemic phase of the disease.

**References**

1. Kirk D, Jones N, Peacock S, Phillips J, Molnár PK, Krkošek M, et al. Empirical evidence that metabolic theory describes the temperature dependency of within-host parasite dynamics. PLoS Biol. 2018 Feb;16(2):e2004608.

2. Kirk D, Luijckx P, Stanic A, Krkošek M. Predicting the Thermal and Allometric Dependencies of Disease Transmission via the Metabolic Theory of Ecology. Am Nat. 2019 May;193(5):661–76.

3. Molnár PK, Sckrabulis JP, Altman KA, Raffel TR. Thermal Performance Curves and the Metabolic Theory of Ecology—A Practical Guide to Models and Experiments for Parasitologists. J Parasitol. 2017 Oct 1;103(5):423–39.

4. Rohr JR, Raffel TR, Blaustein AR, Johnson PTJ, Paull SH, Young S. Using physiology to understand climate-driven changes in disease and their implications for conservation. Conserv Physiol. 2013 Aug 26;1(1):cot022.

5. Kirk D, Luijckx P, Jones N, Krichel L, Pencer C, Molnár P, et al. Experimental evidence of warming-induced disease emergence and its prediction by a trait-based mechanistic model. Proc Biol Sci. 2020 Oct 14;287(1936):20201526.

6. Soetaert K, Petzoldt T, Woodrow Setzer R. Solving Differential Equations in R: Package deSolve. J Stat Softw. 2010 Feb 23;33:1–25.

7. R Core Team. R: A language and environment for statistical computing [Internet]. Vienna, Austria; 2021. Available from: https://www.R-project.org/

8. Ebert D. Ecology, epidemiology and evolution of parasitism in Daphnia [Internet]. 2005 [cited 2022 Feb 22]. Available from: https://edoc.unibas.ch/13505/1/Ebert-Parasitism_in_Daphnia-2005-A4-print.pdf

9. Klüttgen B, Dülmer U, Engels M, Ratte HT. ADaM, an artificial freshwater for the culture of zooplankton. Water Res. 1994 Mar;28(3):743–6.

10. Plummer M, Stukalov A, Denwood M, Plummer MM. Package ‘rjags.’ Update [Internet]. 2019;1. Available from: ftp://freebsd.yz.yamagata-u.ac.jp/pub/cran/web/packages/rjags/rjags.pdf

11. Plummer M, Others. JAGS: A program for analysis of Bayesian graphical models using Gibbs sampling. In: Proceedings of the 3rd international workshop on distributed statistical computing. Vienna, Austria.; 2003. p. 1–10.

12. Sólymos P. Dclone: Data cloning in R. R J. 2010;2(2):29.
